# Copper-Catalyzed Amino-alkoxycarbonylation of Unactivated Alkenes: Synthesis of β‑Amino Esters via Primary Tosyl Amidyl Radical

**DOI:** 10.1021/acs.orglett.6c00774

**Published:** 2026-03-11

**Authors:** Xudong Mao, Ming Hou, Yuanrui Wang, Ren-Guan Miao, Xiao-Feng Wu

**Affiliations:** † Dalian National Laboratory for Clean Energy, 58279 Dalian Institute of Chemical Physics, Chinese Academy of Sciences, Dalian 116023 China; § 28392 Leibniz-Institut für Katalyse e. V., Albert-Einstein-Straβe 29a, 18059 Rostock, Germany

## Abstract

β-Amino acids serve as essential motifs in a wide array of bioactive compounds, pharmaceuticals, and natural products, playing a crucial role in the development of peptides and peptidomimetics. Transition-metal-catalyzed carbonylative functionalization represents an ideal strategy for synthesizing carbonyl-containing compounds. In this context, we disclose a primary tosyl amidyl radical initiated 1,2-amino-alkoxycarbonylation of unactivated alkenes, which offers a straightforward and efficient approach to synthesize β-amino acid derivatives.

## Graphical abstract

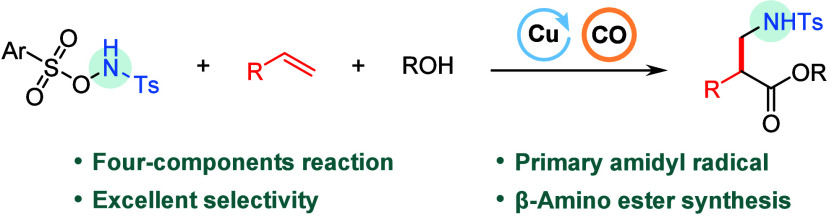

β-Amino acids, prevalent in bioactive natural products, are widely used in peptide therapeutics for their metabolic resilience and drug-like properties.^1–3^ In addition, these compounds are valued as versatile precursors to γ-amino alcohols, β-lactams, and other motifs, which play central roles in catalysis, synthesis, and drug discovery.^4^ During the past decades, numerous catalytic methods have been developed such as the hydrogenation of enamides or amination of α,β-unsaturated carbonyls, which predominantly rely on prefunctionalized substrates and multistep procedures, certainly limiting β-amino acid diverse synthesis.^5–9^ However, the development of direct carboamination protocols starting from simple, nonactivated alkenes presents a more efficient and highly attractive, yet underdeveloped, strategic alternative.

Direct amination of alkenes provides a powerful strategy for C–N bond formation.^10–12^ Over the past decades, the continuous development of alkene amination has led to notable catalytic reactions such as the Sharpless asymmetric aminohydroxylation,^13–15^ aza-Wacker reactions,^16,17^ and metal-catalyzed nitrene transfer from azides,^18^ which enabled efficient access to β-amino alcohols, aziridines, and related nitrogen-containing motifs. Among them, the nitrogen-centered radical (NCR) mediated alkene aminodifunctionalization is a powerful approach to functionalized amines.^19–25^ Following the Hofmann-Löffler-Freytag reaction^26^ discovered at the end of the 19th century, scientists have continuously explored various systems to achieve the generation of nitrogen-centered radicals through classical radical initiation, visible-light photocatalysis, or transition-metal catalysis. Through these catalytic manifolds, a range of alkene difunctionalizations has been enabled, including carboamination,^27–31^ oxyamination,^32–34^ aminofluorination,^35^ and diamination.^36–40^

The simultaneous incorporation of carboxyl and amino groups represents an efficient strategy for the synthesis of β-amino acids, a class of compounds widely embedded in pharmaceuticals, and bioactive peptides have achieved rapid development in recent years.

Concerning the preparation strategies, the utilization of the C1 source to prolong the carbon chain in combination with suitable nitrogen-containing substrates presents an attractive strategy for preparing β-amino acid derivatives, which is of significant interest to both academic and industrial chemists.^41–43^ The use of CO as an abundant and low-cost C1 source in carbonylation reactions provides a straightforward and attractive strategy for the preparation of β-amino acid derivatives.^44–48^ In 2015, Liu and co-workers reported a novel palladium-catalyzed intermolecular aminocarbonylation of alkenes,^49^ enabled by a hypervalent iodine reagent that accelerates the intermolecular aminopalladation step (Scheme 1a). More recently, Beller and co-workers reported a new strategy, accomplishing a copper catalysis and Lewis acid promotion 1,2-amino-alkoxycarbonylation of unactivated alkenes with CO and alkylamines (Scheme 1b).^50^ However, this work employed *O*-benzoylhydroxylamine as the nitrogen-radical precursor that generates a secondary nitrogen-centered radical, and the corresponding transformation with primary amines was not feasible. *N*-Tosyl amines (NHTs) are widely employed in amination chemistry as versatile nitrogen sources and robust protecting groups, and the tosyl group can be conveniently removed under hydrolytic conditions to generate the corresponding amines.^51^ Therefore, we selected this amidyl radical and strategically modulated the electronic properties of the N–O bond^52^ to facilitate its homolytic cleavage, thereby generating an electrophilic nitrogen-centered radical. This radical undergoes regioselective addition to alkenes, enabling a successful copper-catalyzed 1,2-amino-alkoxycarbonylation of alkenes with CO (Scheme 1c). Furthermore, the products can be readily hydrolyzed to afford N-*H* free β-amino acids, thus establishing a practical and general synthetic route to this valuable class of derivatives.

**Scheme 1:**
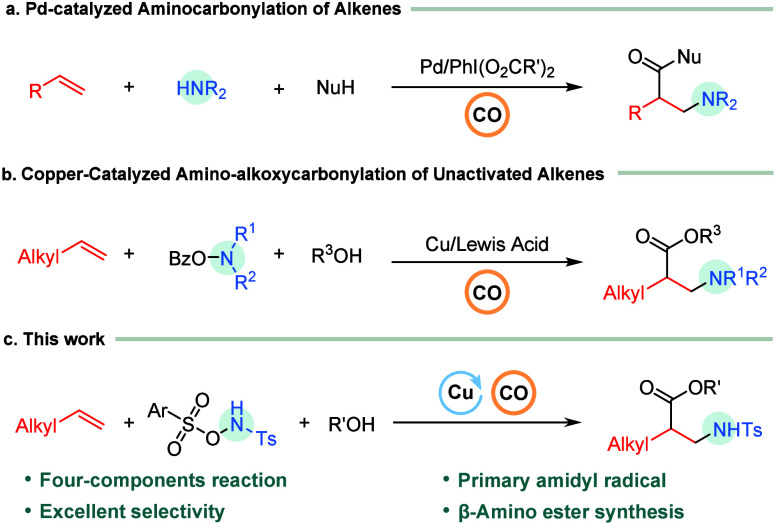
Catalytic Carbonylation of Thioethers

Inspired by previous studies,^50^ a *N*,*O*-disulfonyl nitrogen radical precursor **1a**, styrene derivative **5h**, and *n*-pentanol **3a** were selected as model substrates to optimize this 1,2-amino-alkoxycarbonylation of alkene (Table 1). After systematic evaluation of the reaction parameters, the optimal conditions were identified as follows: Cu­(OTf)_2_ was used as the catalyst, **L1** was used as the ligand, and **P1** was used as a Lewis acid additive in DCM at 80 °C under 60 bar of CO, delivering the desired product in 70% isolated yield after 24 h (Table 1, entry 1). Control experiments revealed that the copper catalyst is indispensable for this transformation (Table 1, entry 2), while the omission of the ligand resulted in a diminished yield of 41% (Table 1, entry 3). The additive of phenylphosphinic acid **P1** (Table 1, entry 4) is likely involved in activating the copper catalyst to promote the reaction, which is also essential for this reaction.^53^ Subsequent evaluation of copper catalyst demonstrated that the different copper catalyst significantly influenced the reaction efficiency, which provided substantially lower yield (Table 1, entries 5–7). Biphenyl and phenanthroline ligands bearing different substituents (**L2**–**L4**) also exerted a notable influence on the reaction yield (Table 1, entries 8–10). In contrast, the use of **P2** severely suppressed the reaction, affording the target product in only 33% yield. Notably, only 21% of the desired **6h** was obtained when the reaction was performed under 20 bar of CO.

**Table 1:**
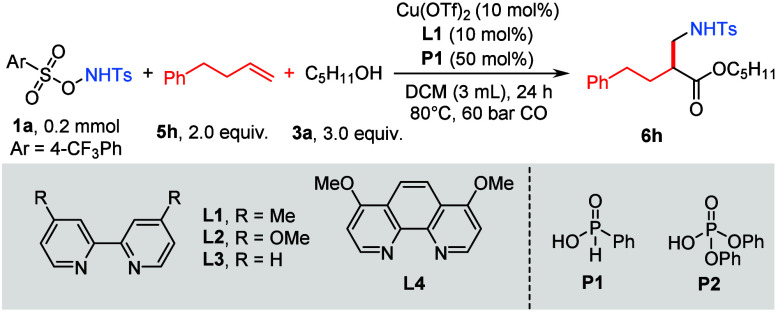
Optimization of Reaction Conditions^a^

Entry	Variation to Standard Conditions	Yield **6h** (%)^c^
1	none	81(70)^b^
2	No Cu(OTf)_2_	ND
3	No **L1**	41
4	No **P1**	62
5	CuOTf instead of Cu(OTf)_2_	71
6	CuCl_2_ instead of Cu(OTf)_2_	47
7	CuTc instead of Cu(OTf)_2_	55
8	**L2** instead of **L1**	69
9	**L3** instead of **L1**	66
10	**L4** instead of **L1**	29
11	**P2** instead of **P1**	33

a Reaction conditions: **1a** (0.2 mmol, 1.0 equiv), **5h** (2.0 equiv), **3a** (3.0 equiv), Cu­(OTf)_2_ (10 mol %), **L1** (10 mol %), **P1** (50 mol %), DCM (3 mL), CO (60 bar), 80 °C, 24 h.

b Yields were determined by GC analysis using hexadecane as an internal standard.

c Isolated yield.

With the optimized conditions established, we next investigated the scope of the alcohols (Scheme 2). A series of linear aliphatic alcohols with different chain lengths were readily accommodated, delivering the corresponding 1,2-amino-alkoxycarbonylation products **4b**–**4f** in moderate to good yields. Cyclic alcohols were also viable substrates, affording products **4g** and **4h** in 54% and 41% yields. The reaction further extended its compatibility with substituted benzyl alcohols: both electron-rich and electron-deficient derivatives underwent smooth conversion to furnish the desired products **4j**–**4m**. Moreover, both phenethyl alcohol and phenylpropanol were successfully transformed to the desired products **4n** and **4o**, respectively. However, the reaction failed when *tert*-butanol was tested as a nucleophile.

**Scheme 2:**
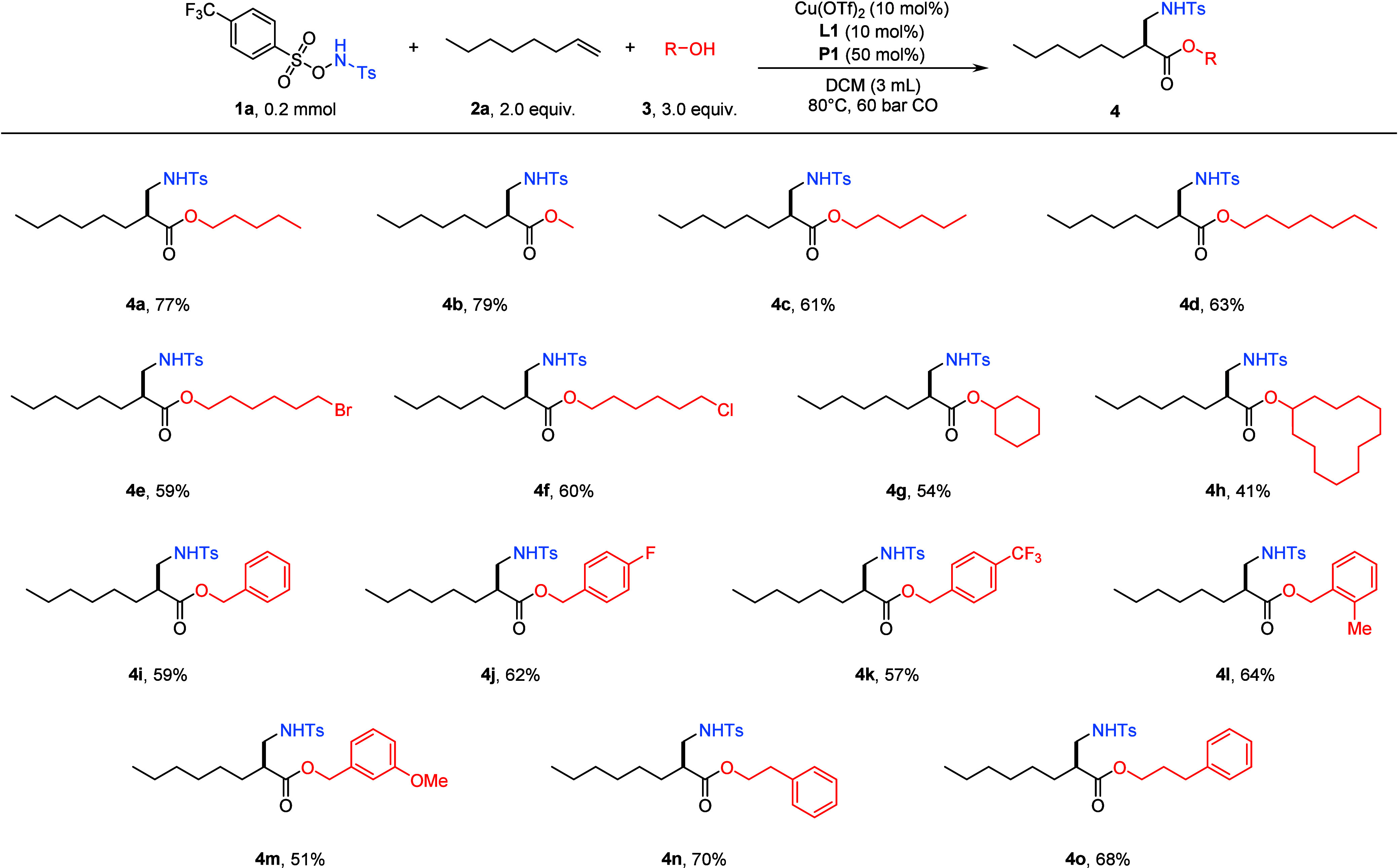
Scope of Alcohols^a^

a Reaction conditions: **1a** (0.2 mmol, 1.0 equiv), **2a** (2.0 equiv), **3** (3.0 equiv), Cu­(OTf)_2_ (10 mol %), **L1** (10 mol %), **P1** (50 mol %), DCM (3 mL), CO (60 bar), 80 °C, 24 h.

We next explored the generality of this system with respect to alkene substrates (Scheme 3). A series of aliphatic alkenes bearing varying carbon-chain lengths were well tolerated under the optimized conditions, smoothly delivering the corresponding 1,2-amino-alkoxycarbonylation products **6a**–**6d** in good yields. Alkenes substituted with cyclohexyl (**6e**) or aryl groups (**6f**–**6h**) also participated efficiently in the transformation, affording the desired products in moderate to good yield, highlighting the compatibility of both alkyl- and aryl-substituted olefins. Notably, ethylene, a simple and abundant feedstock, could be successfully engaged in this reaction to furnish product **6i** in 46% yield. Furthermore, internal alkenes, including cyclopentene and cyclohexene, underwent smooth 1,2-amino-pentoxycarbonylation to afford the corresponding products **6j** and **6k**, demonstrating the applicability of the protocol to more challenging internal olefins. Notably, in the cases of decreased yield of the targeted product generation, the noncarbonylation product was the main identifiable side reaction. It is also worth mentioning that replacing the tosyl group of **1a** with aryl or alkyl led to no desired detectable product.

**Scheme 3:**
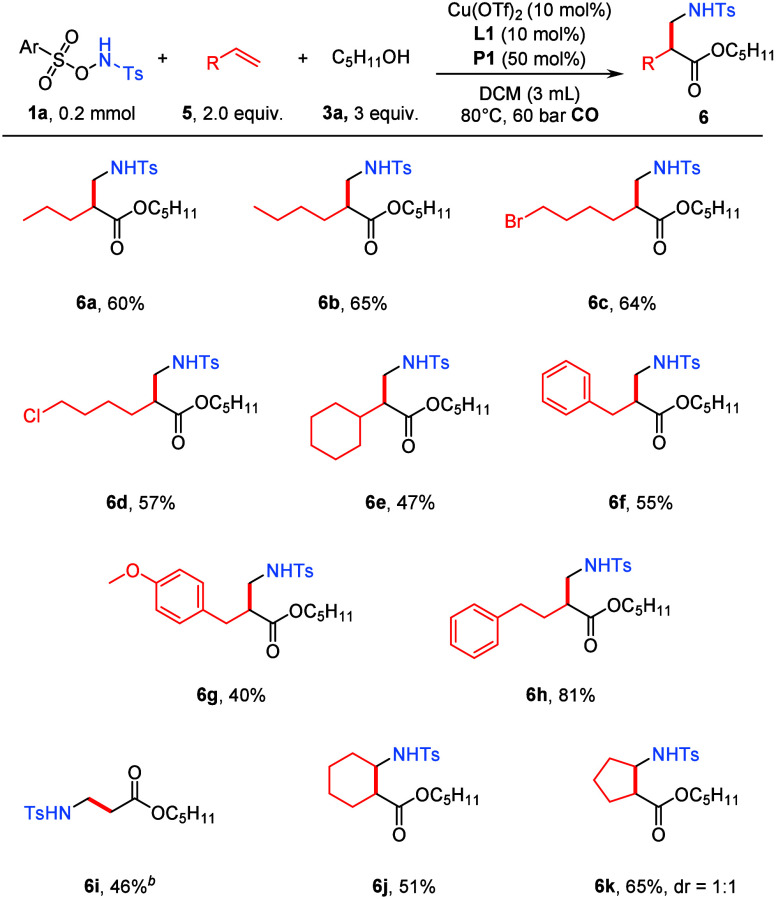
Scope of Alkenes^a^

a Reaction conditions: **1a** (0.2 mmol, 1.0 equiv), **5** (2.0 equiv), **3a** (3.0 equiv), Cu­(OTf)_2_ (10 mol %), **L1** (10 mol %), **P1** (50 mol %), DCM (3 mL), CO (60 bar), 80 °C, 24 h.
b One bar ethylene gas instead of alkenes.

To gain mechanistic insight into the copper-catalyzed 1,2-amino-alkoxycarbonylation of alkenes, a series of control experiments were conducted (Scheme 4a). The addition of the radical scavenger 2,2,6,6-tetramethylpiperidine-1-oxyl (TEMPO) completely suppressed the formation of the desired product **4a**, indicating the involvement of an amidyl radical intermediate. Similarly, butylated hydroxytoluene (BHT) significantly inhibited product formation, and the corresponding BHT-radical adduct derived from tetrahydrothiophene was detected by high-resolution mass spectrometry (HRMS). Furthermore, when 2.0 equiv of diphenylethylene (DPE) was introduced as a radical trap, radical-trapping product **8** was obtained and isolated in 64% yield. Collectively, these results support the participation of amidyl radical species, likely involving an N–O bond homolytic cleavage in this transformation.

**Scheme 4:**
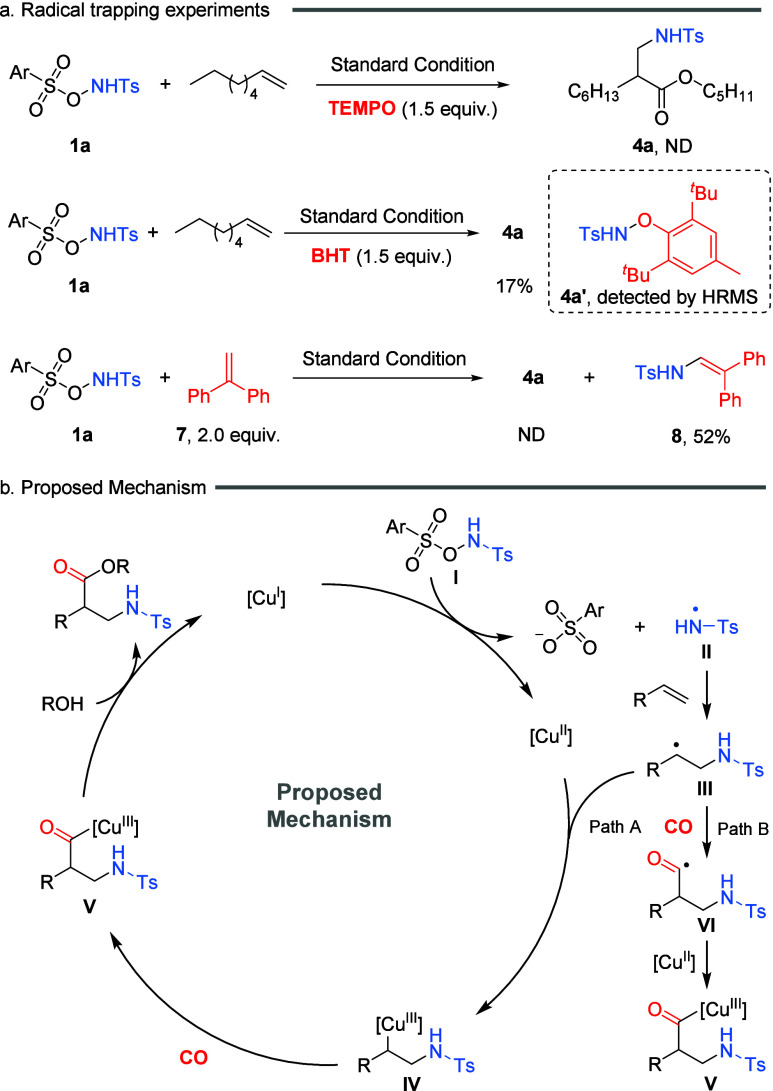
Investigation of the Mechanism

Based on mechanistic investigations and previous reports, we proposed a plausible mechanism for this 1,2-amino-methoxycarbonylation of alkenes (Scheme 4b). First, in situ-generated Cu­(I) complex reacts with *N*-radical precursors **I**, affording a *N*-radical intermediate **II** and Cu­(II) intermediate. This radical intermediate **II** subsequently adds to the unactivated alkene, giving carbon-centered radical intermediate **III**, which might be trapped by Cu­(II) to form alkyl-Cu­(III) intermediate **IV**. This species then undergoes CO insertion to afford acyl-Cu­(III) species **V**. Finally, ligand exchange and reductive elimination deliver the target product and regenerate Cu­(I), thus closing the catalytic cycle. Alternatively, another conceivable pathway involves the direct capture of CO by the radical intermediate **III** after the nitrogen-radical addition to the alkene, forming the corresponding carbonyl radical **VI**, which is subsequently trapped by Cu­(II) and participates in downstream bond-forming events.

In summary, we developed a copper-catalyzed alkene 1,2-amino-alkoxycarbonylation via a primary tosyl amidyl radical with good regioselectivity and excellent functional group tolerance. Mechanistic control experiments suggest that homolytic cleavage of the N–O bond generates a primary amidyl radical that serves as a key intermediate in the formation of diverse β-amino acid derivatives. Through this strategy, a variety of β-amino acid derivatives can be readily accessed after hydrolysis, thereby providing a strategically distinct and practical approach to the synthesis of N-*H* free primary β-amino acids from different unactivated alkenes.

## Supplementary Material



## Data Availability

The data underlying this study are available in the published article and its Supporting Information.

## Notes

General comments, general procedure, optimization details, analytical data, and NMR spectra (PDF)
