# Supplementary material for: Copper-Catalyzed Amino-alkoxycarbonylation of Unactivated Alkenes: Synthesis of β‑Amino Esters via Primary Tosyl Amidyl Radical
Source: Org Lett. 2026 Mar 11;28(11):3649–53. doi: 10.1021/acs.orglett.6c00774 (PMC13010355; doi:10.1021/acs.orglett.6c00774)

Supporting Information

Copper-Catalyzed Amino-alkoxycarbonylation of Unactivated Alkenes: Synthesis of *β*-Amino Esters via Primary Tosyl Amidyl Radical

Xudong Mao,[a,b] Ming Hou,[a,b] Yuanrui Wang,[a] Ren-Guan Miao,[a,b] Xiao-Feng Wu[a,b]*

[a] Dalian National Laboratory for Clean Energy, Dalian Institute of Chemical Physics, Chinese Academy of Sciences, Dalian 116023 China, xwu2020@dicp.ac.cn

[b] Leibniz-Institut für Katalyse e. V., Albert-Einstein-Straβe 29a, 18059 Rostock, Germany

Contents

1. General Information 1

2. Optimization of Reaction Conditions 2

3. Experimental Procedures and Product Characterization 3

4. Mechanistic Studies 4

5. Spectroscopic Data of Products 6

6. Reference 19

7. The NMR Spectrum 20

1. General Information

General. Unless otherwise noted, all reactions were carried out under carbon monoxide or nitrogen atmosphere. All reagents were from commercial sources (Heowns, Rhawn, and Laajoo chemical company) and used as received without further purification. All solvents were dried by standard techniques and distilled prior to use. Column chromatography was performed on silica gel (200-300 meshes) using petroleum ether (bp. 60~90 °C), dichloromethane and ethyl acetate as eluent. All NMR spectra were recorded at ambient temperature using Bruker Avance III 400 MHz NMR (^{1}H, 400 MHz; ^{13}C {^{1}H}, 101 MHz, ^{19}F 376 MHz), Bruker AVANCE III HD 700 MHz NMR spectrometers (^{1}H, 700 MHz; ^{13}C{^{1}H}, 176 MHz). ^{1}H NMR chemical shifts are reported relative to TMS and were referenced via residual proton resonances of the corresponding deuterated solvent ($CDCl_3$: 7.26 ppm; d_6-DMSO: 2.50 ppm) whereas ^{13}C{^{1}H} NMR spectra are reported relative to TMS via the carbon signals of the deuterated solvent ($CDCl_3$: 77.0 ppm; d_6-DMSO: 39.5 ppm). Data for ^{1}H are reported as follows: chemical shift (δ ppm), multiplicity (s = singlet, d = doublet, t = triplet, q = quartet, dd (doublet of doublets), dt (doublet of triplets), m = multiplet), coupling constant (Hz), and integration. All ^{13}C NMR spectra were broad band ^{1}H decoupled. All reactions were monitored by GC-FID or NMR analysis. HRMS data was obtained with Micromass HPLC-Q-TOF mass spectrometer (ESI-TOF) or Agilent 6540 Accurate-MS spectrometer (Q-TOF).

Caution!!! (The high toxicity of carbon monoxide, all the reactions should be performed in an autoclave. The laboratory should be well-equipped with a CO detector and alarm system.)

2. Optimization of Reaction Conditions

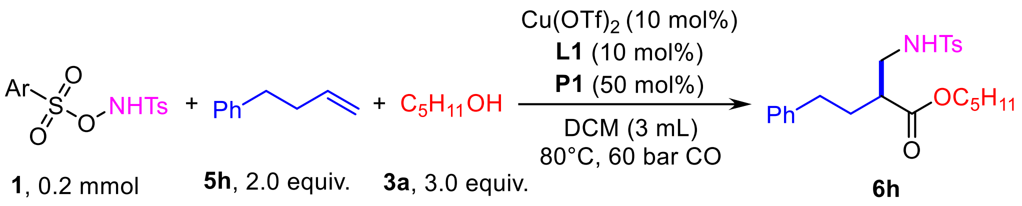

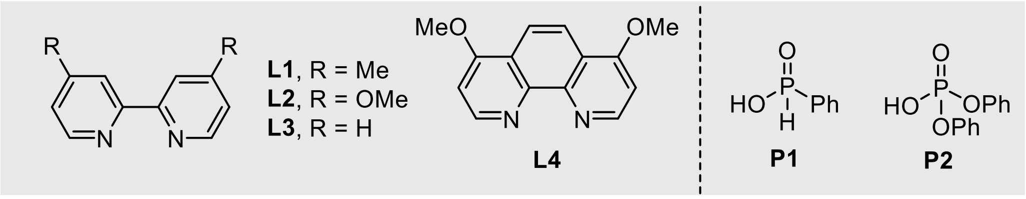

Entry	**Variation to standard conditions**	Yield **3a** (%)[a]
1	none	81(70)[b]
2	No $Cu(OTf)_2$	ND
3	No **L1**	41
4	No **P1**	62
5	CuOTf instead of $Cu(OTf)_2$	71
6	$CuCl_2$ instead of $Cu(OTf)_2$	47
7	CuTc instead of $Cu(OTf)_2$	55
8	**L2** instead of **L1**	69
9	**L3** instead of **L1**	66
10	**L4** instead of **L1**	29
11	**P2** instead of **P1**	33

[a]Yields were determined by GC analysis using hexadecane as an internal standard. [b]Isolated yield.

3. Experimental Procedures and Product Characterization

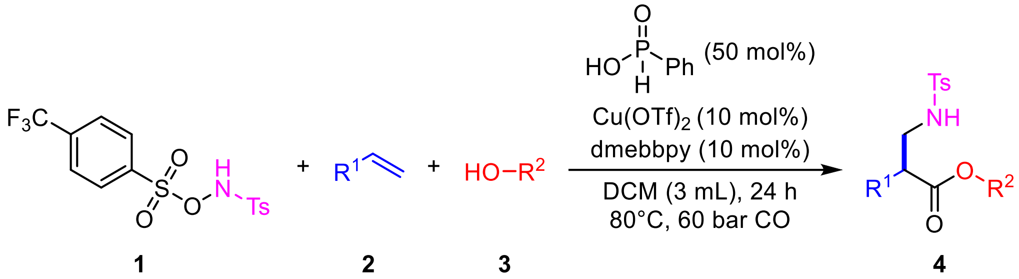

General Procedure: Under an argon atmosphere, a 4 mL screw-cap vial was charged with equipped with a magnetic stir bar was charged with $Cu(OTf)_2$ (7.2 mg, 0.02 mmol, 10 mol%), dmebbpy (3.7 mg, 0.02 mmol, 10 mol%), **1**[1] (1 equiv., 0.2 mmol, 79 mg), **2** alkenes (2 equiv., 0.4 mmol), **3** alcohols (3.0 equiv.), phenylphosphinic acid (14.2 mg, 50 mol%) and DCM (3 mL, 0.067 M) were added nitrogen atmosphere, the vial was moved to an alloy plate and put into a Parr 4560 series autoclave (300 mL) under an argon atmosphere. At room temperature, the autoclave was flushed with CO three times and charged with 60 bar of CO. The autoclave was placed on a heating plate equipped with a magnetic stirrer and an aluminum block. The reaction mixture was heated to 80 ºC for 24 h. After the reaction was complete, the autoclave was cooled down with ice water to room temperature and the pressure was released carefully. After cooling to room temperature, the reaction mixture was directly purified by column chromatography on silica gel using petroleum ether and ethyl acetate to afford the corresponding product.

1 mmol scale reaction: Under an argon atmosphere, a 12 mL screw-cap vial was charged with equipped with a magnetic stir bar was charged with $Cu(OTf)_2$ (10 mol%), dmebbpy (10 mol%), **1** (1 equiv., 1 mmol, 395 mg), **5h** alkene (2 equiv., 2 mmol, 264 mg), **3a** alcohol (3.0 equiv., 3 mmol, 264 mg), phenylphosphinic acid (50 mol%) and DCM (8 mL) were added nitrogen atmosphere, the vial was moved to an alloy plate and put into a Parr 4560 series autoclave (300 mL) under an argon atmosphere. At room temperature, the autoclave was flushed with CO three times and charged with 60 bar of CO. The autoclave was placed on a heating plate equipped with a magnetic stirrer and an aluminum block. The reaction mixture was heated to 80 ºC for 24 h. After the reaction was complete, the autoclave was cooled down with ice water to room temperature and the pressure was released carefully. After cooling to room temperature, the reaction mixture was directly purified by column chromatography on silica gel using petroleum ether and ethyl acetate to afford the corresponding product **6h** in 75% yield (312.75 mg).

4. Mechanistic Studies

Radical trapping experiment

Following the standard procedure of the model reaction, when 1.5 equiv. of radical inhibitor 2,2,6,6-tetramethylpiperidine-1-oxy (TEMPO) was added to the reaction mixture, the formation of the desired product **3a** was completely inhibited. When 2,6-di-tert-butyl-4-methylphenol (BHT) was added to the reaction mixture, the formation of the desired product **3a** was partly inhibited. The adducts of tetrahydrothiophene radical by BHT were confirmed by high-resolution mass spectrometry (HRMS).

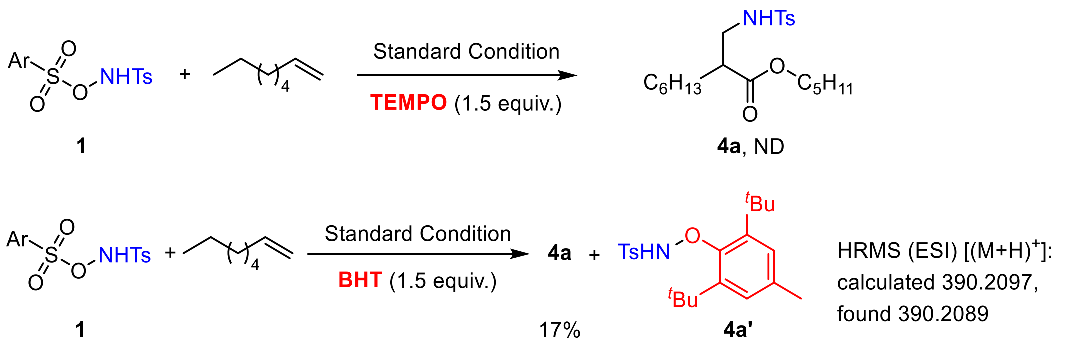

Following the standard procedure of the model reaction, when 0.2 mmol of radical inhibitor 1,1-diphenylethylene (DPE) was added to the reaction mixture, the formation of the desired product **4a** was completely inhibited, and the coupling product **8** was obtained in 52% yield.

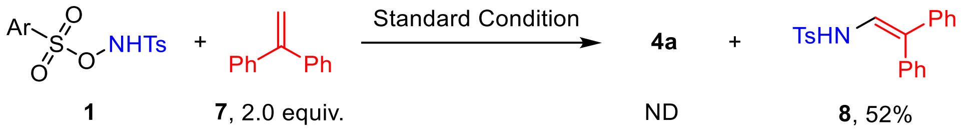

N-(2,2-Diphenylvinyl)-4-methylbenzenesulfonamide (8)

36.3 mg, 52% yield, a light yellow oil, eluent: petroleum ether/ethyl acetate = 10:1.

^{1}H NMR (400 MHz, $CDCl_3$) δ 7.72 (d, *J* = 8.1 Hz, 2H), 7.40-7.30 (m, 5H), 7.26-7.18

(m, 3H), 7.14-7.07 (m, 2H), 6.96-6.87 (m, 2H), 6.80 (d, *J* = 11.6 Hz, 1H), 6.29 (d, *J* = 11.6 Hz, 1H), 2.45 (s, 3H); ^{13}C NMR (101 MHz, $CDCl_3$) δ 144.0, 139.4, 136.7, 136.3, 129.9, 129.6, 129.4, 128.4, 128.2, 127.1, 126.8, 126.5, 126.1, 120.2, 21.6.

5. Spectroscopic Data of Products

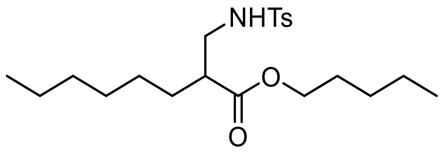

Pentyl 2-(((4-methylphenyl)sulfonamido)methyl)octanoate (4a) Prepared according to the general procedure from **1a** (0.20 mmol), **2a** (2.0 equiv.) and **3a** (3.0 equiv.) purified by column chromatography on silica gel with petroleum ether/ethyl acetate (10:1) to provide the title compound **4a** as a light yellow oil (61.2 mg, 77% yield); ^{1}H NMR (400 MHz, $CDCl_3$) δ 7.73 (d, *J* = 8.4 Hz, 2H), 7.30 (d, *J* = 8.0 Hz, 2H), 5.01 (t, *J* = 6.6 Hz, 1H), 4.12-3.97 (m, 2H), 3.08 (t, *J* = 6.5 Hz, 2H), 2.58-2.49 (m, 1H), 2.43 (s, 3H), 1.64-1.55 (m, 3H), 1.50-1.43 (m, 1H), 1.34-1.29 (m, 4H), 1.26-1.21 (m, 8H), 0.92-0.85 (m, 6H); ^{13}C NMR (101 MHz, $CDCl_3$) δ 174.7, 143.4, 137.0, 129.7, 127.0, 65.0, 45.0, 43.7, 31.5, 29.6, 29.0, 28.2, 28.0, 26.8, 22.5, 22.2, 21.5, 14.0, 13.9; HRMS (ESI) Calcd for $C_{21}H_{36}NO_4S$ $[M + H]^+$ 398.2360, found 398.2369.

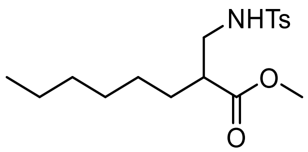

Methyl 2-(((4-methylphenyl)sulfonamido)methyl)octanoate (4b) Prepared according to the general procedure from **1a** (0.20 mmol), **2a** (2.0 equiv.) and **3b** (3.0 equiv.) purified by column chromatography on silica gel with petroleum ether/ethyl acetate (10:1) to provide the title compound **4b** as a light yellow oil (53.9 mg, 79% yield); ^{1}H NMR (400 MHz, $CDCl_3$) δ 7.74 (d, *J* = 8.0 Hz, 2H), 7.31 (d, *J* = 7.9 Hz, 2H), 5.05 (t, *J* = 6.6 Hz, 1H), 3.65 (s, 3H), 3.08 (t, *J* = 6.5 Hz, 2H), 2.61-2.51 (m, 1H), 2.43 (s, 3H), 1.63-1.41 (m, 2H), 1.31-1.17 (m, 8H), 0.87 (t, *J* = 6.8 Hz, 3H); ^{13}C NMR (101 MHz, $CDCl_3$) δ 175.1, 143.4, 137.0, 129.7, 127.0, 51.9, 45.0, 43.7, 31.5, 29.5, 29.0, 26.8, 22.5, 21.5, 14.0; HRMS (ESI) Calcd for $C_{17}H_{28}NO_4S$ $[M + H]^+$ 342.1734, found 342.1735.

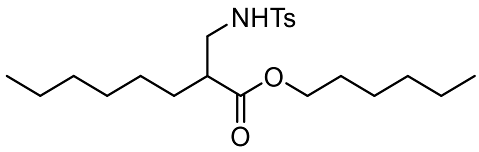

Hexyl 2-(((4-methylphenyl)sulfonamido)methyl)octanoate (4c) Prepared according to the general procedure from **1a** (0.20 mmol), **2a** (2.0 equiv.) and **3c** (3.0 equiv.) purified by column chromatography on silica gel with petroleum ether/ethyl acetate (10:1) to provide the title compound **4c** as a light yellow oil (50.1 mg, 61 % yield); ^{1}H NMR (400 MHz, $CDCl_3$) δ 7.73 (d, *J* = 8.0 Hz, 2H), 7.30 (d, *J* = 8.0 Hz, 2H), 5.07 (t, *J* = 6.6 Hz, 1H), 4.10-3.96 (m, 2H), 3.07 (t, *J* = 6.4 Hz, 2H), 2.58-2.49 (m, 1H), 2.42 (s, 3H), 1.58 (q, *J* = 6.9 Hz, 3H), 1.49-1.43 (m, 1H), 1.29 (s, 6H), 1.27-1.18 (m, 8H), 0.92-0.85 (m, 6H); ^{13}C NMR (101 MHz, $CDCl_3$) δ 174.7, 143.3, 137.0, 129.7, 127.0, 65.0, 45.0, 43.7, 31.5, 31.3, 29.5, 29.0, 28.4, 26.7, 25.5, 22.49, 22.46, 21.5, 14.0, 13.9; HRMS (ESI) Calcd for $C_{22}H_{38}NO_4S$ $[M + H]^+$ 412.2516, found 412.2519.

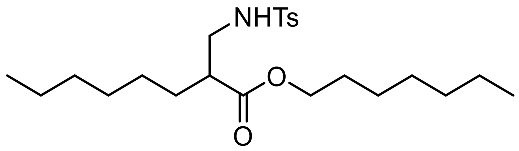

Heptyl 2-(((4-methylphenyl)sulfonamido)methyl)octanoate (4d) Prepared according to the general procedure from **1a** (0.20 mmol), **2a** (2.0 equiv.) and **3d** (3.0 equiv.) purified by column chromatography on silica gel with petroleum ether/ethyl acetate (10:1) to provide the title compound **4d** as a light yellow oil (53.6 mg, 63 % yield); ^{1}H NMR (400 MHz, $CDCl_3$) δ 7.73 (d, *J* = 8.0 Hz, 2H), 7.31 (d, *J* = 8.0 Hz, 2H), 4.96 (t, *J* = 6.6 Hz, 1H), 4.10-3.97 (m, 2H), 3.08 (t, *J* = 6.4 Hz, 2H), 2.59-2.49 (m, 1H), 2.43 (s, 3H), 1.59 (d, *J* = 8.4 Hz, 3H), 1.47 (dd, *J* = 14.0, 6.7 Hz, 1H), 1.33-1.26 (m, 9H), 1.26-1.19 (m, 7H), 0.88 (q, *J* = 6.7 Hz, 6H); ^{13}C NMR (101 MHz, $CDCl_3$) δ 174.8, 143.4, 137.0, 129.7, 127.0, 65.0, 45.0, 43.7, 31.7, 31.5, 29.6, 29.0, 28.9, 28.5, 26.8, 25.8, 22.6, 22.5, 21.5, 14.03, 14.01; HRMS (ESI) Calcd for $C_{23}H_{40}NO_4S$ $[M + H]^+$ 426.2673, found 426.2674.

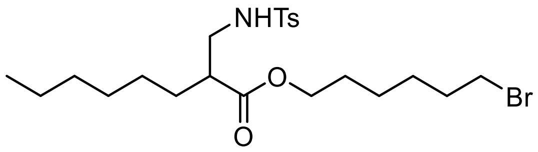

6-Bromohexyl 2-(((4-methylphenyl)sulfonamido)methyl)octanoatee (4e) Prepared according to the general procedure from **1a** (0.20 mmol), **2a** (2.0 equiv.) and **3e** (3.0 equiv.) purified by column chromatography on silica gel with petroleum ether/ethyl acetate (10:1) to provide the title compound **4e** as a light yellow oil (58.1 mg, 59% yield); ^{1}H NMR (400 MHz, $CDCl_3$) δ 7.73 (d, *J* = 8.1 Hz, 2H), 7.31 (d, *J* = 8.0 Hz, 2H), 5.03 (t, *J* = 6.6 Hz, 1H), 4.12-4.00 (m, 2H), 3.41 (t, *J* = 6.7 Hz, 2H), 3.07 (t, *J* = 6.5 Hz, 2H), 2.60-2.51 (m, 1H), 2.43 (s, 3H), 1.91-1.82 (m, 2H), 1.67-1.55 (m, 3H), 1.51-1.43 (m, 3H), 1.40-1.34 (m, 2H), 1.30-1.20 (m, 8H), 0.87 (t, *J* = 6.8 Hz, 3H); ^{13}C NMR (101 MHz, $CDCl_3$) δ 174.7, 143.4, 136.9, 129.7, 127.0, 64.7, 45.0, 43.7, 33.6, 32.5, 31.5, 29.5, 29.0, 28.3, 27.7, 26.8, 25.1, 22.5, 21.5, 14.0; HRMS (ESI) Calcd for $C_{22}H_{37}BrNO_4S$ $[M + H]^+$ 490.1621, found 490.1627.

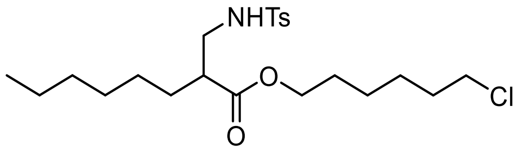

6-Chlorohexyl 2-(((4-methylphenyl)sulfonamido)methyl)octanoate (4f) Prepared according to the general procedure from **1a** (0.20 mmol), **2a** (2.0 equiv.) and **3f** (3.0 equiv.) purified by column chromatography on silica gel with petroleum ether/ethyl acetate (10:1) to provide the title compound **4f** as a light yellow oil (53.4 mg, 60% yield); ^{1}H NMR (400 MHz, $CDCl_3$) δ 7.73 (d, *J* = 8.0 Hz, 2H), 7.31 (d, *J* = 7.9 Hz, 2H), 5.03 (t, *J* = 6.6 Hz, 1H), 4.13-3.98 (m, 2H), 3.54 (t, *J* = 6.6 Hz, 2H), 3.07 (t, *J* = 6.5 Hz, 2H), 2.60-2.50 (m, 1H), 2.43 (s, 3H), 1.84-1.72 (m, 2H), 1.66-1.54 (m, 3H), 1.52-1.42 (m, 3H), 1.37 (q, *J* = 8.0 Hz, 2H), 1.31-1.20 (m, 8H), 0.87 (t, *J* = 6.8 Hz, 3H); ^{13}C NMR (101 MHz, $CDCl_3$) δ 174.7, 143.4, 136.9, 129.7, 127.02, 126.98, 64.7, 45.0, 44.9, 43.7, 32.3, 31.5, 29.5, 29.0, 28.3, 26.8, 26.4, 25.2, 22.5, 21.5, 14.0; HRMS (ESI) Calcd for $C_{22}H_{37}ClNO_4S$ $[M + H]^+$ 446.2126, found 446.2127.

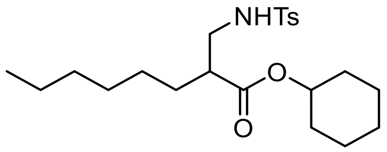

Cyclohexyl 2-(((4-methylphenyl)sulfonamido)methyl)octanoate (4g) Prepared according to the general procedure from **1a** (0.20 mmol), **2a** (2.0 equiv.) and **3g** (3.0 equiv.) purified by column chromatography on silica gel with petroleum ether/ethyl acetate (10:1) to provide the title compound **4g** as a light yellow oil (44.2 mg, 54% yield); ^{1}H NMR (400 MHz, $CDCl_3$) δ 7.73 (d, *J* = 8.1 Hz, 2H), 7.30 (d, *J* = 8.0 Hz, 2H), 5.01 (t, *J* = 6.5 Hz, 1H), 4.78-4.68 (m, 1H), 3.07 (t, *J* = 6.4 Hz, 2H), 2.50 (dd, *J* = 13.4, 6.8 Hz, 1H), 2.42 (s, 3H), 1.77 (q, *J* = 4.8, 3.9 Hz, 2H), 1.72-1.66 (m, 2H), 1.59-1.50 (m, 2H), 1.42-1.33 (m, 4H), 1.27 (d, *J* = 7.7 Hz, 3H), 1.23 (d, *J* = 3.8 Hz, 7H), 0.87 (t, *J* = 6.8 Hz, 3H); ^{13}C NMR (101 MHz, $CDCl_3$) δ 174.1, 143.3, 137.0, 129.7, 127.0, 73.1, 45.0, 43.8, 31.5, 31.4, 29.6, 29.0, 26.7, 25.2, 23.6, 22.5, 21.5, 14.0; HRMS (ESI) Calcd for $C_{22}H_{36}NO_4S$ $[M + H]^+$ 410.2360, found 410.2359.

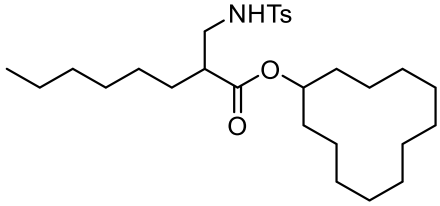

Cyclododecyl 2-(((4-methylphenyl)sulfonamido)methyl)octanoate (4h) Prepared according to the general procedure from **1a** (0.20 mmol), **2a** (2.0 equiv.) and **3h** (3.0 equiv.) purified by column chromatography on silica gel with petroleum ether/ethyl acetate (10:1) to provide the title compound **4h** as a light yellow oil (40.5 mg, 41% yield); ^{1}H NMR (400 MHz, $CDCl_3$) δ 7.73 (d, *J* = 7.9 Hz, 2H), 7.30 (d, *J* = 8.0 Hz, 2H), 5.05-4.89 (m, 2H), 3.15-2.98 (m, 2H), 2.56-2.46 (m, 1H), 2.43 (s, 3H), 1.74-1.61 (m, 2H), 1.61-1.56 (m, 1H), 1.49-1.19 (m, 29H), 0.87 (t, *J* = 6.8 Hz, 3H); ^{13}C NMR (101 MHz, $CDCl_3$) δ 174.5, 143.4, 137.0, 129.7, 127.0, 73.0, 45.0, 43.8, 31.6, 29.6, 29.2, 29.1, 29.0, 26.8, 24.04, 24.01, 23.8, 23.4, 23.3, 23.2, 23.1, 22.5, 21.5, 20.9, 14.0; HRMS (ESI) Calcd for $C_{28}H_{48}NO_4S$ $[M + H]^+$ 494.3299, found 494.3298.

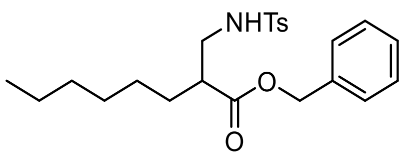

Benzyl 2-(((4-methylphenyl)sulfonamido)methyl)octanoate (4i) Prepared according to the general procedure from **1a** (0.20 mmol), **2a** (2.0 equiv.) and **3i** (3.0 equiv.) purified by column chromatography on silica gel with petroleum ether/ethyl acetate (10:1) to provide the title compound **4i** as a light yellow oil (49.2 mg, 59% yield); ^{1}H NMR (400 MHz, $CDCl_3$) δ 7.70 (d, J = 8.0 Hz, 2H), 7.36 (d, J = 7.2 Hz, 2H), 7.34-7.28 (m, 4H), 7.27 (s, 1H), 5.14-5.04 (m, 2H), 4.90 (t, J = 6.6 Hz, 1H), 3.09 (t, J = 6.5 Hz, 2H), 2.63-2.55 (m, 1H), 2.41 (s, 3H), 1.64-1.59 (m, 1H), 1.51-1.44 (m, 1H), 1.28-1.23 (m, 3H), 1.19 (s, 5H), 0.85 (t, J = 6.9 Hz, 3H); ^{13}C NMR (101 MHz, $CDCl_3$) δ 174.5, 143.4, 137.0, 135.5, 129.7, 128.6, 128.4, 128.2, 127.0, 66.6, 45.0, 43.7, 31.5, 29.5, 29.0, 26.7, 22.5, 21.5, 14.0; HRMS (ESI) Calcd for $C_{23}H_{32}NO_4S$ [M + H]$^+$ 418.2047, found 418.2047.

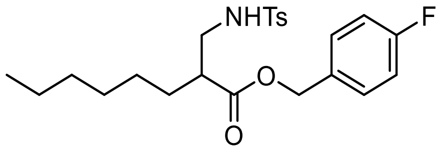

4-Fluorobenzyl 2-(((4-methylphenyl)sulfonamido)methyl)octanoate (4j) Prepared according to the general procedure from **1a** (0.20 mmol), **2a** (2.0 equiv.) and **3j** (3.0 equiv.) purified by column chromatography on silica gel with petroleum ether/ethyl acetate (10:1) to provide the title compound **4j** as a light yellow oil (53.9 mg, 62% yield); ^{1}H NMR (400 MHz, $CDCl_3$) δ 7.70 (d, J = 8.0 Hz, 2H), 7.35-7.26 (m, 4H), 7.04 (t, J = 8.5 Hz, 2H), 5.05 (q, J = 12.1 Hz, 2H), 4.88 (t, J = 6.6 Hz, 1H), 3.08 (t, J = 6.5 Hz, 2H), 2.66-2.53 (m, 1H), 2.42 (s, 3H), 1.63-1.56 (m, 1H), 1.51-1.42 (m, 1H), 1.27-1.15 (m, 8H), 0.85 (t, J = 6.9 Hz, 3H); ^{13}C NMR (176 MHz, $CDCl_3$) δ 174.4, 162.7 (d, $J_{C\text{-}F}$ = 243 Hz), 143.5, 136.9, 131.4 (d, $J_{C\text{-}F}$ = 3 Hz), 130.27 (d, $J_{C\text{-}F}$ = 7 Hz), 129.73, 127.00, 115.57 (d, $J_{C\text{-}F}$ = 23 Hz), 65.9, 45.0, 43.7, 31.5, 29.5, 29.0, 26.7, 22.5, 21.5, 14.0; ^{19}F NMR (376 MHz, $CDCl_3$) δ -113.23; HRMS (ESI) Calcd for $C_{23}H_{31}FNO_4S$ [M + H]$^+$ 436.1952, found 436.1954.

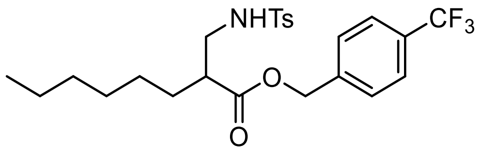

4-(Trifluoromethyl)benzyl 2-(((4-methylphenyl)sulfonamido)methyl)-octanoate (4k) Prepared according to the general procedure from **1a** (0.20 mmol), **2a** (2.0 equiv.) and **3k** (3.0 equiv.) purified by column chromatography on silica gel with petroleum ether/ethyl acetate (10:1) to provide the title compound **4k** as a light yellow oil (55.3 mg, 57% yield); ^{1}H NMR (400 MHz, $CDCl_3$) δ 7.71 (d, *J* = 8.1 Hz, 2H), 7.62 (d, *J* = 8.0 Hz, 2H), 7.43 (d, *J* = 8.0 Hz, 2H), 7.29 (d, *J* = 8.0 Hz, 2H), 5.21-5.09 (m, 2H), 4.92 (t, *J* = 6.7 Hz, 1H), 3.10 (t, *J* = 6.5 Hz, 2H), 2.71-2.60 (m, 1H), 2.42 (s, 3H), 1.64-1.58 (m, 1H), 1.53-1.45 (m, 1H), 1.27-1.15 (m, 8H), 0.85 (t, *J* = 6.9 Hz, 3H); ^{13}C NMR (101 MHz, $CDCl_3$) δ 174.3, 143.5, 136.9, 130.5 (q, $J_{C\text{-}F}$ = 33 Hz), 129.8, 128.2, 127.0, 125.6 (q, $J_{C\text{-}F}$ = 4 Hz), 123.9 (q, $J_{C\text{-}F}$ = 270 Hz), 65.6, 53.4, 45.15, 43.75, 31.5, 29.6, 29.0, 26.7, 22.5, 21.5, 14.0; ^{19}F NMR (376 MHz, $CDCl_3$) δ -62.67; HRMS (ESI) Calcd for $C_{24}H_{31}F_3NO_4S$ $[M + H]^+$ 486.1920, found 486.1926.

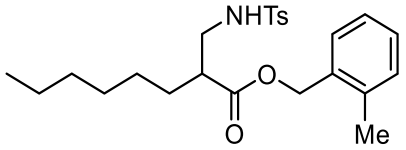

2-Methylbenzyl 2-(((4-methylphenyl)sulfonamido)methyl)octanoate (4l) Prepared according to the general procedure from **1a** (0.20 mmol), **2a** (2.0 equiv.) and **3l** (3.0 equiv.) purified by column chromatography on silica gel with petroleum ether/ethyl acetate (10:1) to provide the title compound **4l** as a light yellow oil (55.2 mg, 64% yield); ^{1}H NMR (400 MHz, $CDCl_3$) δ 7.70 (d, *J* = 8.0 Hz, 2H), 7.29-7.25 (m, 4H), 7.20 (d, *J* = 7.3 Hz, 2H), 5.16-5.05 (m, 2H), 4.89 (t, *J* = 6.7 Hz, 1H), 3.09 (t, *J* = 6.5 Hz, 2H), 2.62-2.55 (m, 1H), 2.41 (s, 3H), 2.32 (s, 3H), 1.62-1.56 (m, 1H), 1.51-1.43 (m, 1H), 1.25-1.16 (m, 8H), 0.85 (t, *J* = 7.0 Hz, 3H); ^{13}C NMR (101 MHz, $CDCl_3$) δ 174.5, 143.4, 137.0, 136.9, 133.4, 130.4, 129.7, 129.3, 128.7, 127.0, 126.1, 65.1, 45.0, 43.7, 31.5, 29.6, 29.0, 26.7, 22.5, 21.5, 18.9, 14.0; HRMS (ESI) Calcd for $C_{24}H_{34}NO_4S$ $[M + H]^+$ 432.2203, found 432.2193.

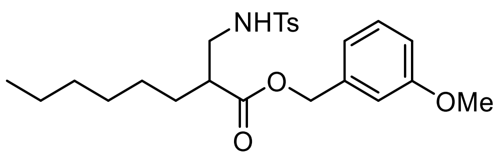

3-Methoxybenzyl 2-(((4-methylphenyl)sulfonamido)methyl)octanoate (4m) Prepared according to the general procedure from **1a** (0.20 mmol), **2a** (2.0 equiv.) and **3m** (3.0 equiv.) purified by column chromatography on silica gel with petroleum ether/ethyl acetate (10:1) to provide the title compound **4m** as a light yellow oil (45.6 mg, 51% yield); ^{1}H NMR (400 MHz, $CDCl_3$) δ 7.70 (d, *J* = 8.3 Hz, 2H), 7.29 (d, *J* = 6.1 Hz, 3H), 6.92-6.82 (m, 3H), 5.11-5.01 (m, 2H), 4.90 (t, *J* = 6.7 Hz, 1H), 3.81 (s, 3H), 3.10 (t, *J* = 6.3 Hz, 2H), 2.64-2.55 (m, 1H), 2.41 (s, 3H), 1.65-1.57 (m, 1H), 1.52-1.44 (m, 1H), 1.27-1.16 (m, 8H), 0.85 (t, *J* = 6.8 Hz, 3H); ^{13}C NMR (101 MHz, $CDCl_3$) δ 174.4, 159.8, 143.4, 137.0, 129.7, 129.7, 127.0, 120.3, 113.9, 113.6, 66.5, 55.2, 45.0, 43.7, 31.5, 29.5, 29.0, 26.7, 22.5, 21.5, 14.0; HRMS (ESI) Calcd for $C_{24}H_{34}NO_5S$ $[M + H]^+$ 448.2152, found 448.2161.

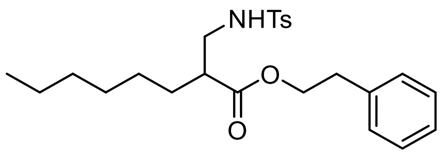

Phenethyl 2-(((4-methylphenyl)sulfonamido)methyl)octanoate (4n) Prepared according to the general procedure from **1a** (0.20 mmol), **2a** (2.0 equiv.) and **3n** (3.0 equiv.) purified by column chromatography on silica gel with petroleum ether/ethyl acetate (10:1) to provide the title compound **4n** as a light yellow oil (60.3 mg, 70% yield); ^{1}H NMR (400 MHz, $CDCl_3$) δ 7.70 (d, *J* = 8.0 Hz, 2H), 7.29 (d, *J* = 7.7 Hz, 4H), 7.24 (d, *J* = 7.1 Hz, 1H), 7.19 (d, *J* = 7.6 Hz, 2H), 4.84 (t, *J* = 6.7 Hz, 1H), 4.33-4.24 (m, 2H), 3.03 (t, *J* = 6.2 Hz, 2H), 2.91 (t, *J* = 6.9 Hz, 2H), 2.54-2.47 (m, 1H), 2.42 (s, 3H), 1.54-1.36 (m, 2H), 1.29-1.22 (m, 2H), 1.21-1.13 (m, 6H), 0.87 (t, *J* = 7.0 Hz, 3H); ^{13}C NMR (101 MHz, $CDCl_3$) δ 174.5, 143.4, 137.5, 137.0, 129.7, 128.8, 128.6, 127.0, 126.7, 65.1, 45.1, 43.7, 34.9, 31.5, 29.5, 29.0, 26.7, 22.5, 21.5, 14.0; HRMS (ESI) Calcd for $C_{24}H_{34}NO_4S$ $[M + H]^+$ 432.2203, found 432.2199.

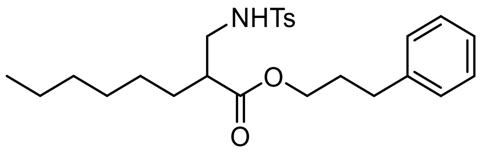

3-Phenylpropyl 2-(((4-methylphenyl)sulfonamido)methyl)octanoate (4o) Prepared according to the general procedure from **1a** (0.20 mmol), **2a** (2.0 equiv.) and **3o** (3.0 equiv.) purified by column chromatography on silica gel with petroleum ether/ethyl acetate (10:1) to provide the title compound **4o** as a light yellow oil (60.5 mg, 68% yield); ^{1}H NMR (400 MHz, $CDCl_3$) δ 7.73 (d, *J* = 8.0 Hz, 2H), 7.32-7.26 (m, 4H), 7.21 (d, *J* = 7.2 Hz, 1H), 7.16 (d, *J* = 7.7 Hz, 2H), 4.98 (t, *J* = 6.6 Hz, 1H), 4.11-4.02 (m, 2H), 3.08 (t, *J* = 6.5 Hz, 2H), 2.66 (t, *J* = 7.7 Hz, 2H), 2.59-2.52 (m, 1H), 2.41 (s, 3H), 1.97-1.90 (m, 2H), 1.61-1.42 (m, 2H), 1.25 (d, *J* = 7.8 Hz, 8H), 0.87 (t, *J* = 6.5 Hz, 3H); ^{13}C NMR (101 MHz, $CDCl_3$) δ 174.7, 143.4, 140.9, 137.0, 129.7, 128.5, 128.3, 127.0, 126.1, 64.2, 45.0, 43.7, 32.1, 31.5, 30.1, 29.6, 29.0, 26.8, 22.5, 21.5, 14.0; HRMS (ESI) Calcd for $C_{25}H_{36}NO_4S$ $[M + H]^+$ 446.2360, found 446.2362.

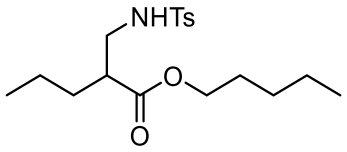

Pentyl 2-(((4-methylphenyl)sulfonamido)methyl)pentanoate (6a) Prepared according to the general procedure from **1a** (0.20 mmol), **5a** (2.0 equiv.) and **3a** (3.0 equiv.) purified by column chromatography on silica gel with petroleum ether/ethyl acetate (10:1) to provide the title compound **6a** as a light yellow oil (42.6 mg, 60% yield); ^{1}H NMR (400 MHz, $CDCl_3$) δ 7.76-7.69 (m, 2H), 7.31 (d, *J* = 8.0 Hz, 2H), 5.00 (t, *J* = 6.6 Hz, 1H), 4.10-3.99 (m, 2H), 3.07 (t, *J* = 6.4 Hz, 2H), 2.60-2.52 (m, 1H), 2.43 (s, 3H), 1.63-1.53 (m, 3H), 1.49-1.43 (m, 1H), 1.35-1.26 (m, 6H), 0.91 (t, *J* = 6.1 Hz, 3H), 0.89-0.84 (m, 3H); ^{13}C NMR (101 MHz, $CDCl_3$) δ 174.8, 143.4, 137.0, 129.7, 127.0, 65.0, 44.7, 43.7, 31.6, 28.2, 28.0, 22.2, 21.5, 20.0, 13.9, 13.8; HRMS (ESI) Calcd for $C_{18}H_{30}NO_4S$ $[M + H]^+$ 356.1890, found 356.1891.

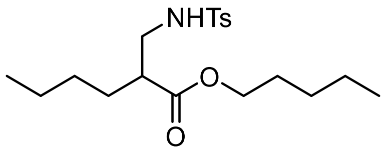

Pentyl (*S*)-2-(((4-methylphenyl)sulfonamido)methyl)hexanoate (6b) Prepared according to the general procedure from **1a** (0.20 mmol), **5b** (2.0 equiv.) and **3a** (3.0 equiv.) purified by column chromatography on silica gel with petroleum ether/ethyl acetate (10:1) to provide the title compound **6b** as a light yellow oil (48.0 mg, 65% yield); ^{1}H NMR (400 MHz, $CDCl_3$) δ 7.73 (d, *J* = 8.3 Hz, 2H), 7.31 (d, *J* = 8.0 Hz, 2H), 5.03 (t, *J* = 6.6 Hz, 1H), 4.11-3.98 (m, 2H), 3.08 (t, *J* = 6.4 Hz, 2H), 2.60-2.49 (m, 1H), 2.43 (s, 3H), 1.63-1.54 (m, 3H), 1.52-1.44 (m, 1H), 1.37-1.27 (m, 5H), 1.26-1.21 (m, 3H), 0.90 (t, *J* = 6.9 Hz, 3H), 0.86 (t, *J* = 7.0 Hz, 3H); ^{13}C NMR (101 MHz, $CDCl_3$) δ 174.7, 143.4, 137.0, 129.7, 127.0, 65.0, 44.9, 43.7, 29.2, 28.9, 28.2, 28.0, 22.4, 22.2, 21.5, 13.9, 13.8; HRMS (ESI) Calcd for $C_{19}H_{32}NO_4S$ $[M + H]^+$ 370.2047, found 370.2043.

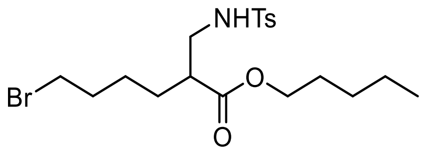

Pentyl 6-bromo-2-(((4-methylphenyl)sulfonamido)methyl)hexanoate (6c) Prepared according to the general procedure from **1a** (0.20 mmol), **5c** (2.0 equiv.) and **3a** (3.0 equiv.) purified by column chromatography on silica gel with petroleum ether/ethyl acetate (10:1) to provide the title compound **6c** as a light yellow oil (57.2 mg, 64% yield); ^{1}H NMR (400 MHz, $CDCl_3$) δ 7.73 (d, *J* = 8.1 Hz, 2H), 7.32 (d, *J* = 7.9 Hz, 2H), 4.96 (t, *J* = 6.6 Hz, 1H), 4.13-3.98 (m, 2H), 3.37 (t, *J* = 6.7 Hz, 2H), 3.09 (t, *J* = 6.4 Hz, 2H), 2.61-2.50 (m, 1H), 2.43 (s, 3H), 1.87-1.77 (m, 2H), 1.66-1.58 (m, 3H), 1.56-1.49 (m, 1H), 1.48-1.39 (m, 2H), 1.36-1.27 (m, 4H), 0.91 (t, *J* = 6.8 Hz, 3H); ^{13}C NMR (101 MHz, $CDCl_3$) δ 174.4, 143.5, 136.9, 129.8, 127.0, 65.2, 44.7, 43.6, 33.1, 32.3, 28.6, 28.2, 28.0, 25.4, 22.3, 21.5, 13.9; HRMS (ESI) Calcd for $C_{19}H_{31}BrNO_4S$ $[M + H]^+$ 448.1152, found 448.1155.

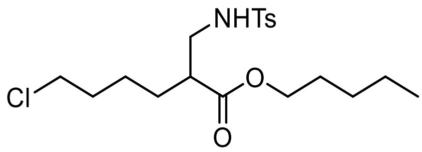

Pentyl 6-chloro-2-(((4-methylphenyl)sulfonamido)methyl)hexanoate (6d) Prepared according to the general procedure from **1a** (0.20 mmol), **5d** (2.0 equiv.) and **3a** (3.0 equiv.) purified by column chromatography on silica gel with petroleum ether/ethyl acetate (10:1) to provide the title compound **6d** as a light yellow oil (46.1 mg, 57% yield); ^{1}H NMR (400 MHz, $CDCl_3$) δ 7.74 (d, *J* = 8.0 Hz, 2H), 7.31 (d, *J* = 7.9 Hz, 2H), 5.12 (t, *J* = 6.6 Hz, 1H), 4.12-3.98 (m, 2H), 3.49 (t, *J* = 6.5 Hz, 2H), 3.18-2.96 (m, 2H), 2.64-2.50 (m, 1H), 2.43 (s, 3H), 1.75-1.70 (m, 2H), 1.65-1.49 (m, 4H), 1.47-1.38 (m, 2H), 1.35-1.27 (m, 4H), 0.90 (t, *J* = 6.8 Hz, 3H); ^{13}C NMR (101 MHz, $CDCl_3$) δ 174.3, 143.4, 136.9, 129.7, 127.0, 65.1, 44.8, 44.5, 43.6, 32.1, 28.6, 28.1, 28.0, 24.1, 22.2, 21.5, 13.9; HRMS (ESI) Calcd for $C_{19}H_{31}ClNO_4S$ $[M + H]^+$ 404.1657, found 404.1662.

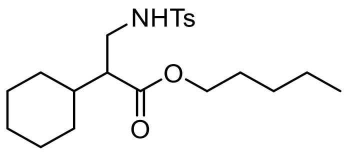

Pentyl 2-cyclohexyl-3-((4-methylphenyl)sulfonamido)propanoate (6e) Prepared according to the general procedure from **1a** (0.20 mmol), **5e** (2.0 equiv.) and **3a** (3.0 equiv.) purified by column chromatography on silica gel with petroleum ether/ethyl acetate (10:1) to provide the title compound **6e** as a light yellow oil (37.2 mg, 47% yield); ^{1}H NMR (400 MHz, $CDCl_3$) δ 7.73 (d, *J* = 8.2 Hz, 2H), 7.31 (d, *J* = 8.1 Hz, 2H), 4.95 (t, *J* = 6.5 Hz, 1H), 4.16-3.89 (m, 2H), 3.23-3.00 (m, 2H), 2.45-2.37 (m, 4H), 1.72-1.53 (m, 8H), 1.36-1.28 (m, 4H), 1.22-1.06 (m, 3H), 1.04-0.94 (m, 2H), 0.90 (t, *J* = 6.8 Hz, 3H); ^{13}C NMR (101 MHz, $CDCl_3$) δ 174.3, 143.3, 136.9, 129.7, 127.0, 64.9, 50.9, 41.9, 38.1, 30.6, 30.1, 28.2, 28.0, 26.2, 26.1, 26.0, 22.2, 21.5, 13.9; HRMS (ESI) Calcd for $C_{21}H_{34}NO_4S$ $[M + H]^+$ 396.2203, found 396.2202.

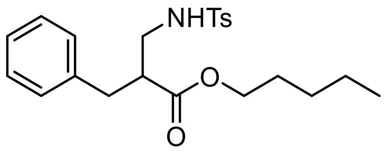

Pentyl 2-benzyl-3-((4-methylphenyl)sulfonamido)propanoate (6f) Prepared according to the general procedure from **1a** (0.20 mmol), **5f** (2.0 equiv.) and **3a** (3.0 equiv.) purified by column chromatography on silica gel with petroleum ether/ethyl acetate (10:1) to provide the title compound **6f** as a light yellow oil (44.3 mg, 55% yield); ^{1}H NMR (400 MHz, $CDCl_3$) δ 7.67 (d, *J* = 7.9 Hz, 2H), 7.30-7.24 (m, 4H), 7.23-7.19 (m, 1H), 7.10 (d, *J* = 6.5 Hz, 2H), 5.03 (t, *J* = 6.5 Hz, 1H), 4.07-3.94 (m, 2H), 3.12-3.00 (m, 2H), 2.99-2.92 (m, 1H), 2.87-2.74 (m, 2H), 2.42 (s, 3H), 1.56-1.48 (m, 2H), 1.29 (q, *J* = 7.6, 7.1 Hz, 2H), 1.25-1.17 (m, 2H), 0.88 (t, *J* = 7.1 Hz, 3H); ^{13}C NMR (101 MHz, $CDCl_3$) δ 173.9, 143.4, 137.7, 136.8, 129.7, 128.9, 128.5, 127.0, 126.7, 65.2, 46.7, 43.2, 35.5, 28.1, 27.9, 22.2, 21.5, 13.9; HRMS (ESI) Calcd for $C_{22}H_{30}NO_4S$ $[M + H]^+$ 404.1890, found 404.1897.

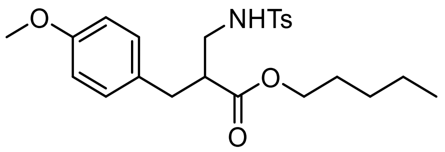

Pentyl 2-(4-methoxybenzyl)-3-((4-methylphenyl)sulfonamido)propanoate (6g) Prepared according to the general procedure from **1a** (0.20 mmol), **5g** (2.0 equiv.) and **3a** (3.0 equiv.) purified by column chromatography on silica gel with petroleum ether/ethyl acetate (10:1) to provide the title compound **6g** as a light yellow oil (34.7 mg, 40% yield); ^{1}H NMR (400 MHz, $CDCl_3$) δ 7.67 (d, *J* = 8.0 Hz, 2H), 7.28 (d, *J* = 7.9 Hz, 2H), 7.02 (d, *J* = 8.3 Hz, 2H), 6.80 (d, *J* = 8.2 Hz, 2H), 4.99 (t, *J* = 6.6 Hz, 1H), 4.08-3.93 (m, 2H), 3.78 (s, 3H), 3.14-2.99 (m, 2H), 2.90 (dd, *J* = 12.5, 5.2 Hz, 1H), 2.83-2.70 (m, 2H), 2.42 (s, 3H), 1.58-1.49 (m, 2H), 1.34-1.20 (m, 4H), 0.89 (t, *J* = 7.1 Hz, 3H); ^{13}C NMR (101 MHz, $CDCl_3$) δ 174.0, 158.4, 143.4, 136.8, 129.9, 129.70, 129.66, 127.0, 113.9, 65.2, 55.2, 46.8, 43.2, 34.7, 28.1, 27.9, 22.2, 21.5, 13.9; HRMS (ESI) Calcd for $C_{23}H_{32}NO_5S$ $[M + H]^+$ 434.1996, found 434.1996.

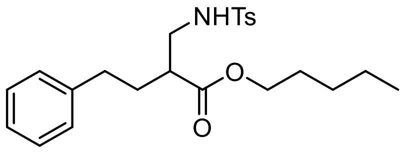

Pentyl 2-(((4-methylphenyl)sulfonamido)methyl)-4-phenylbutanoate (6h) Prepared according to the general procedure from **1a** (0.20 mmol), **5h** (2.0 equiv.) and **3a** (3.0 equiv.) purified by column chromatography on silica gel with petroleum ether/ethyl acetate (10:1) to provide the title compound **6h** as a light yellow oil (67.5 mg, 81% yield); ^{1}H NMR (400 MHz, $CDCl_3$) δ 7.72 (d, *J* = 8.2 Hz, 2H), 7.30-7.25 (m, 4H), 7.22-7.17 (m, 1H), 7.15-7.10 (m, 2H), 5.01 (t, *J* = 6.6 Hz, 1H), 4.11-3.98 (m, 2H), 3.19-3.05 (m, 2H), 2.64-2.53 (m, 3H), 2.42 (s, 3H), 2.00-1.90 (m, 1H), 1.87-1.75 (m, 1H), 1.67-1.55 (m, 3H), 1.34-1.30 (m, 3H), 0.90 (t, *J* = 6.8 Hz, 3H); ^{13}C NMR (101 MHz, $CDCl_3$) δ 174.4, 143.4, 140.8, 136.9, 129.7, 128.4, 128.3, 127.0, 126.1, 65.2, 44.3, 43.6, 32.9, 31.0, 28.2, 28.0, 22.2, 21.5, 13.9; HRMS (ESI) Calcd for $C_{23}H_{32}NO_4S$ $[M + H]^+$ 418.2047, found 418.2046.

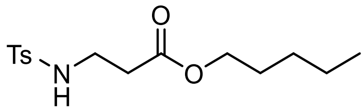

Pentyl 3-((4-methylphenyl)sulfonamido)propanoate (6i) Prepared according to the general procedure from **1a** (0.20 mmol), **5i** (2.0 equiv.) and **3a** (3.0 equiv.) purified by column chromatography on silica gel with petroleum ether/ethyl acetate (10:1) to provide the title compound **6i** as a light yellow oil (28.8 mg, 46% yield); ^{1}H NMR (400 MHz, $CDCl_3$) δ 7.67 (d, *J* = 8.3 Hz, 2H), 7.24 (d, *J* = 8.1 Hz, 2H), 5.14 (t, *J* = 6.6 Hz, 1H), 3.98 (t, *J* = 6.8 Hz, 2H), 3.11 (q, *J* = 6.3 Hz, 2H), 2.45 (t, *J* = 6.0 Hz, 2H), 2.35 (s, 3H), 1.57-1.48 (m, 2H), 1.27-1.19 (m, 4H), 0.85-0.80 (m, 3H); ^{13}C NMR (101 MHz, $CDCl_3$) δ 172.1, 143.4, 136.9, 129.7, 127.0, 65.1, 38.8, 33.9, 28.1, 27.9, 22.2, 21.5, 13.9; HRMS (ESI) Calcd for $C_{15}H_{24}NO_4S$ $[M + H]^+$ 314.1421, found 314.1424.

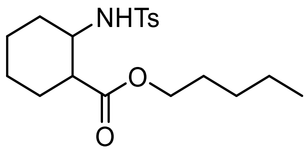

Pentyl 2-((4-methylphenyl)sulfonamido)cyclohexane-1-carboxylate (6j) Prepared according to the general procedure from **1a** (0.20 mmol), **5j** (2.0 equiv.) and **3a** (3.0 equiv.) purified by column chromatography on silica gel with petroleum ether/ethyl acetate (10:1) to provide the title compound **6j** as a light yellow oil (37.2 mg, 51% yield); ^{1}H NMR (400 MHz, $CDCl_3$) δ 7.74 (d, *J* = 8.3 Hz, 2H), 7.28 (d, *J* = 8.8 Hz, 2H), 5.56 (d, *J* = 8.9 Hz, 1H), 4.10-3.99 (m, 1H), 3.95-3.86 (m, 1H), 3.49-3.36 (m, 1H), 2.63 (q, *J* = 4.8 Hz, 1H), 2.42 (s, 3H), 2.05-1.95 (m, 1H), 1.82-1.72 (m, 1H), 1.69-1.63 (m, 1H), 1.60-1.54 (m, 2H), 1.54-1.47 (m, 2H), 1.45-1.37 (m, 1H), 1.36-1.23 (m, 6H), 0.91 (t, *J* = 6.9 Hz, 3H); ^{13}C NMR (101 MHz, $CDCl_3$) δ 173.7, 143.1, 138.6, 129.6, 126.9, 64.8, 52.5, 45.0, 30.0, 28.1, 28.0, 27.2, 23.7, 22.3, 22.2, 21.5, 13.9; HRMS (ESI) Calcd for $C_{19}H_{30}NO_4S$ $[M + H]^+$ 368.1890, found 368.1894.

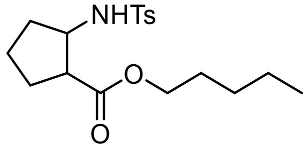

Pentyl 2-((4-methylphenyl)sulfonamido)cyclopentane-1-carboxylate (6k) Prepared according to the general procedure from **1a** (0.20 mmol), **5k** (2.0 equiv.) and **3a** (3.0 equiv.) purified by column chromatography on silica gel with petroleum ether/ethyl acetate (10:1) to provide the title compound **6k** as a light yellow oil (45.9 mg, 65% yield, dr = 1:1); **6k** (cis): ^{1}H NMR (400 MHz, $CDCl_3$) δ 7.73 (d, *J* = 8.3 Hz, 2H), 7.28 (d, *J* = 8.1 Hz, 2H), 5.49 (d, *J* = 8.7 Hz, 1H), 4.13-3.84 (m, 2H), 3.82-3.67 (m, 1H), 2.81-2.68 (m, 1H), 2.42 (s, 3H), 1.97-1.78 (m, 2H), 1.82-1.64 (m, 3H), 1.62-1.46 (m, 4H), 1.38-1.22 (m, 4H), 0.91 (t, *J* = 7.0 Hz, 3H); ^{13}C NMR (101 MHz, $CDCl_3$) δ 174.4, 143.2, 137.9, 129.6, 127.0, 65.0, 56.1, 46.1, 32.1, 28.1, 28.0, 22.3, 21.5, 21.5, 13.9; **6k** (trans): ^{1}H NMR (400 MHz, $CDCl_3$) δ 7.76 (d, *J* = 8.3 Hz, 2H), 7.30 (d, *J* = 8.1 Hz, 2H),

4.92 (d, J = 6.1 Hz, 1H), 4.02-3.90 (m, 2H), 3.77-3.67 (m, 1H), 2.69-2.60 (m, 1H), 2.43 (s, 3H), 2.05-1.91 (m, 2H), 1.81-1.72 (m, 1H), 1.72-1.62 (m, 2H), 1.61-1.54 (m, 2H), 1.53-1.43 (m, 1H), 1.36-1.26 (m, 4H), 0.91 (t, J = 6.9 Hz, 3H); ^{13}C NMR (101 MHz, $CDCl_3$) δ 174.1, 143.4, 137.1, 129.6, 127.3, 65.0, 57.7, 50.8, 33.5, 28.2, 28.0, 27.9, 22.8, 22.3, 21.5, 13.9; HRMS (ESI) Calcd for $C_{18}H_{28}NO_4S$ $[M + H]^+$ 354.1734, found 354.1735.

6. Reference

(1) Li S., Xu X., Chen J., et al. Visible-Light-Promoted α-C(sp^3)-H Amidation of Cyclic Ethers under Redox-Neutral Conditions. *Organic Letters*, **2025**, *27*, 2863-2867.

7.NMR Spectra

^{1}H NMR (400 MHz, $CDCl_3$) and ^{13}C NMR (101 MHz, $CDCl_3$) spectrum of 4a

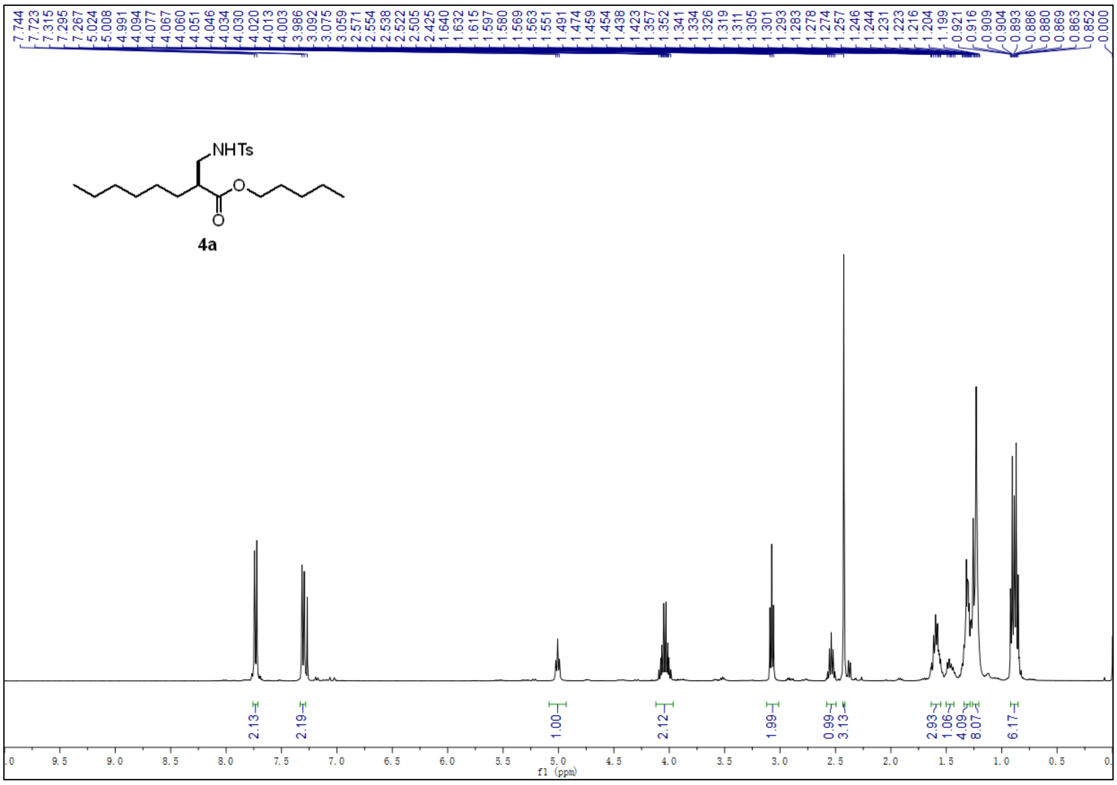

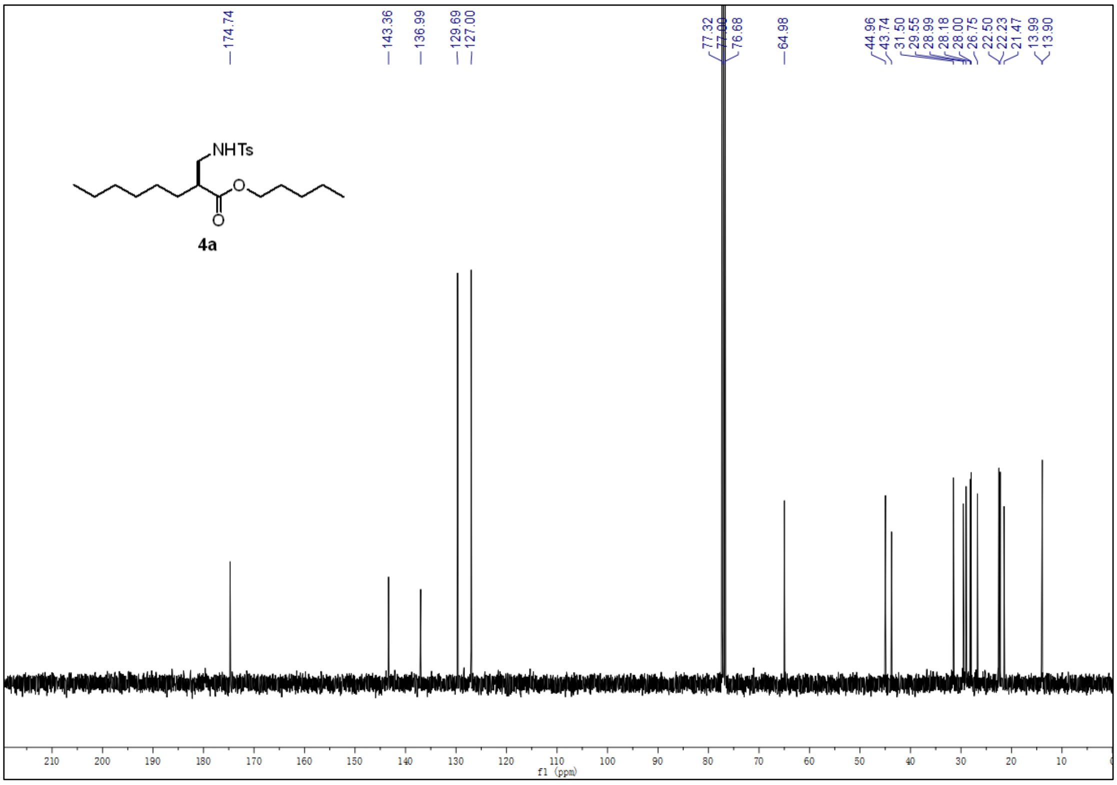

^{1}H NMR (400 MHz, CDCl$_3$) and ^{13}C NMR (101 MHz, CDCl$_3$) spectrum of 4b

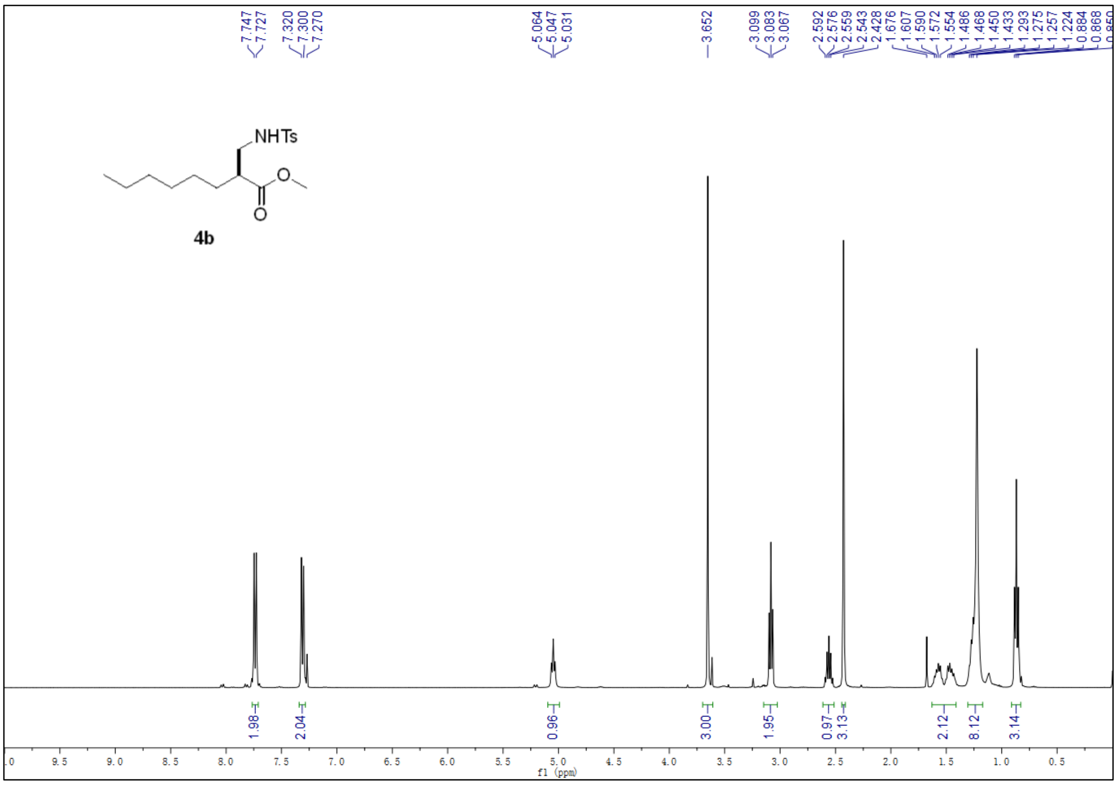

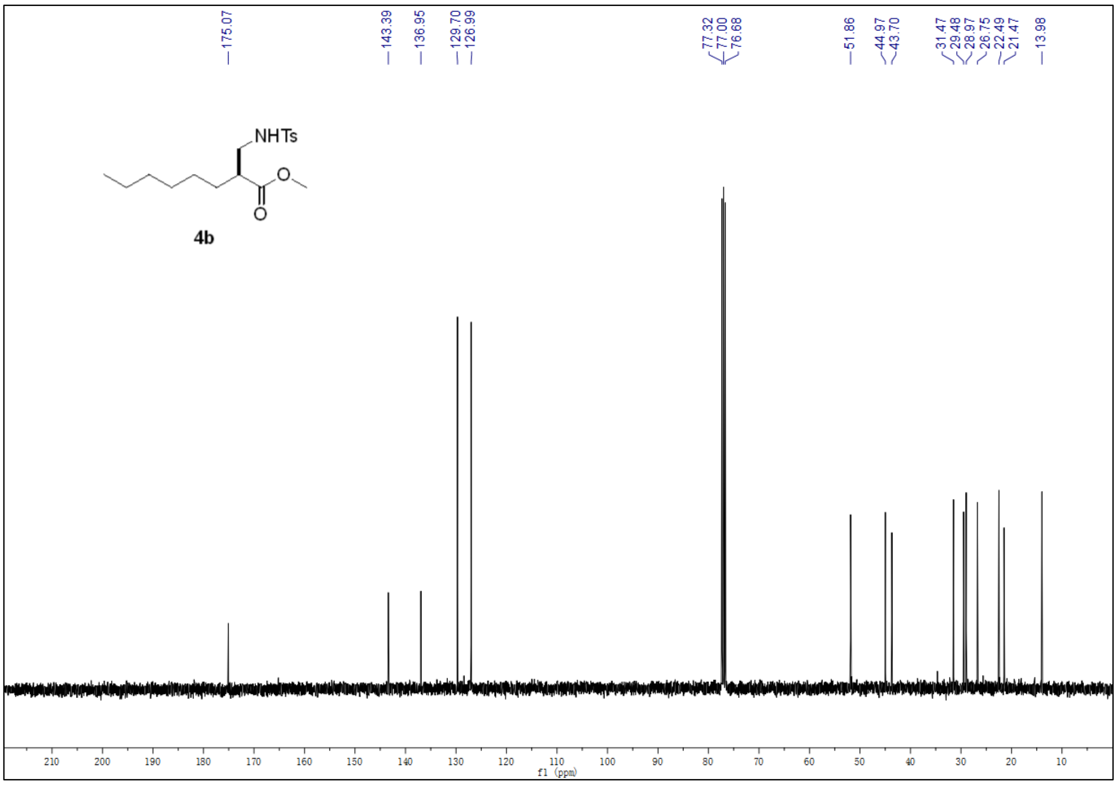

^{1}H NMR (400 MHz, CDCl₃) and ^{13}C NMR (101 MHz, CDCl₃) spectrum of 4c

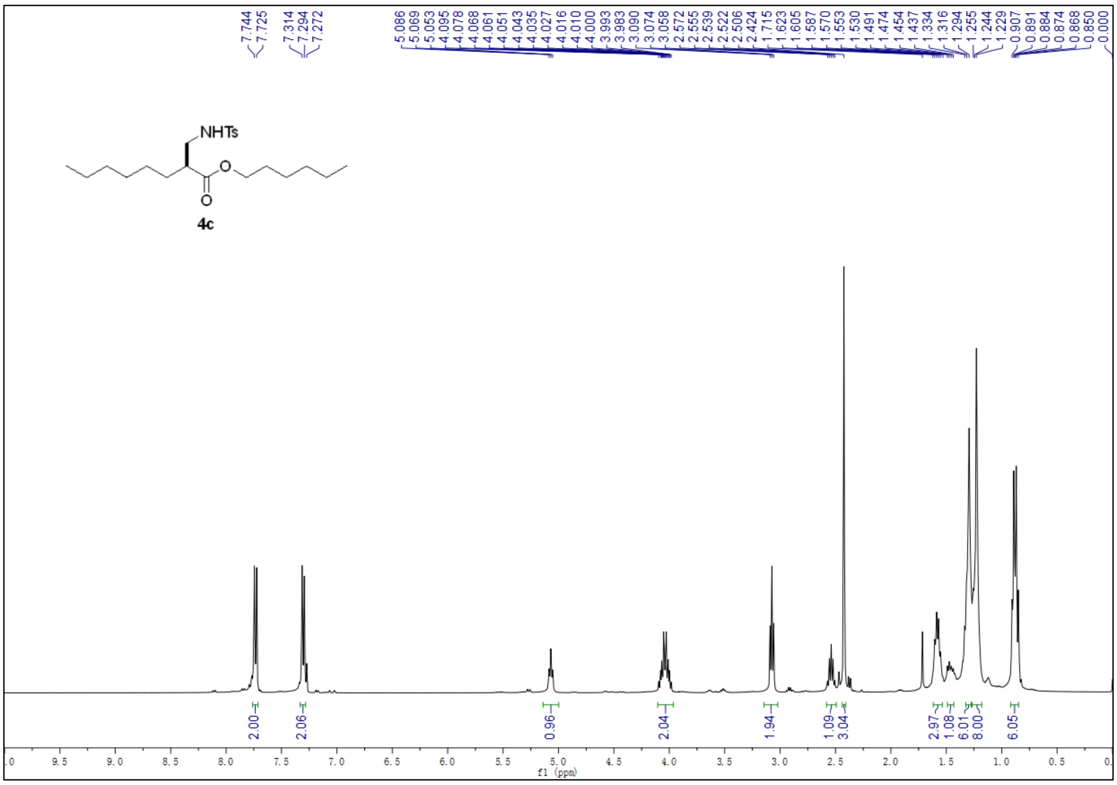

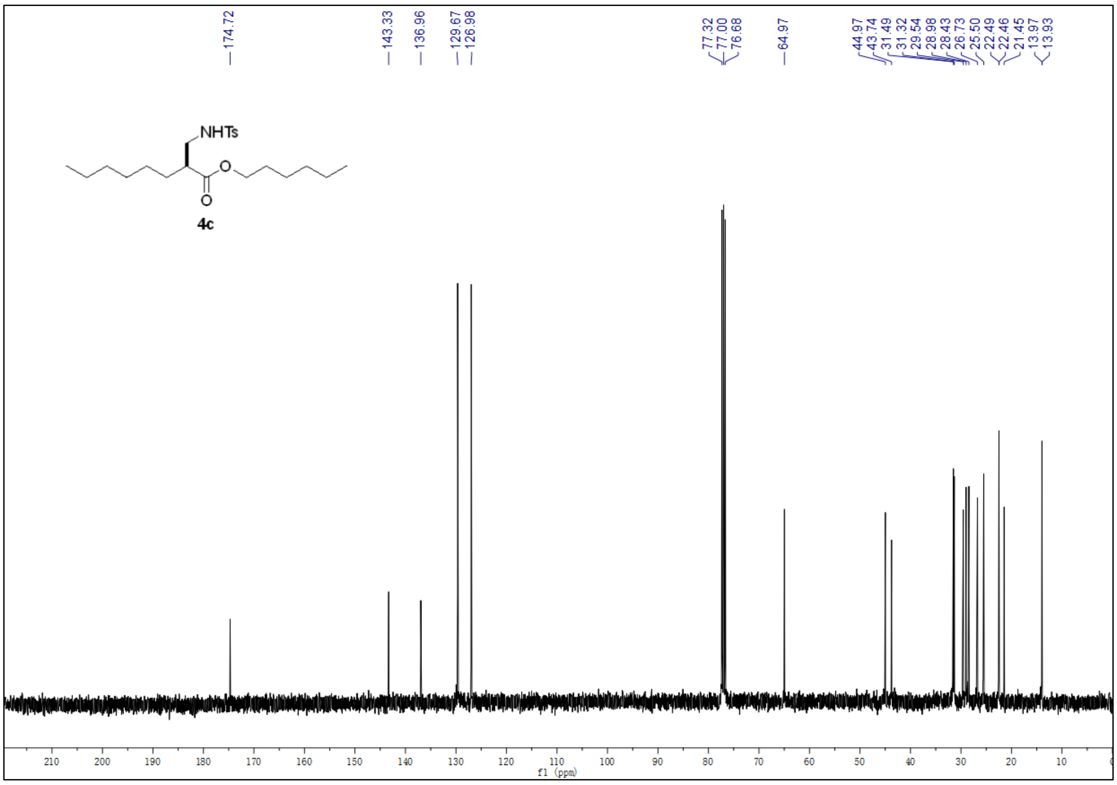

^{1}H NMR (400 MHz, CDCl3) and ^{13}C NMR (101 MHz, CDCl3) spectrum of 4d

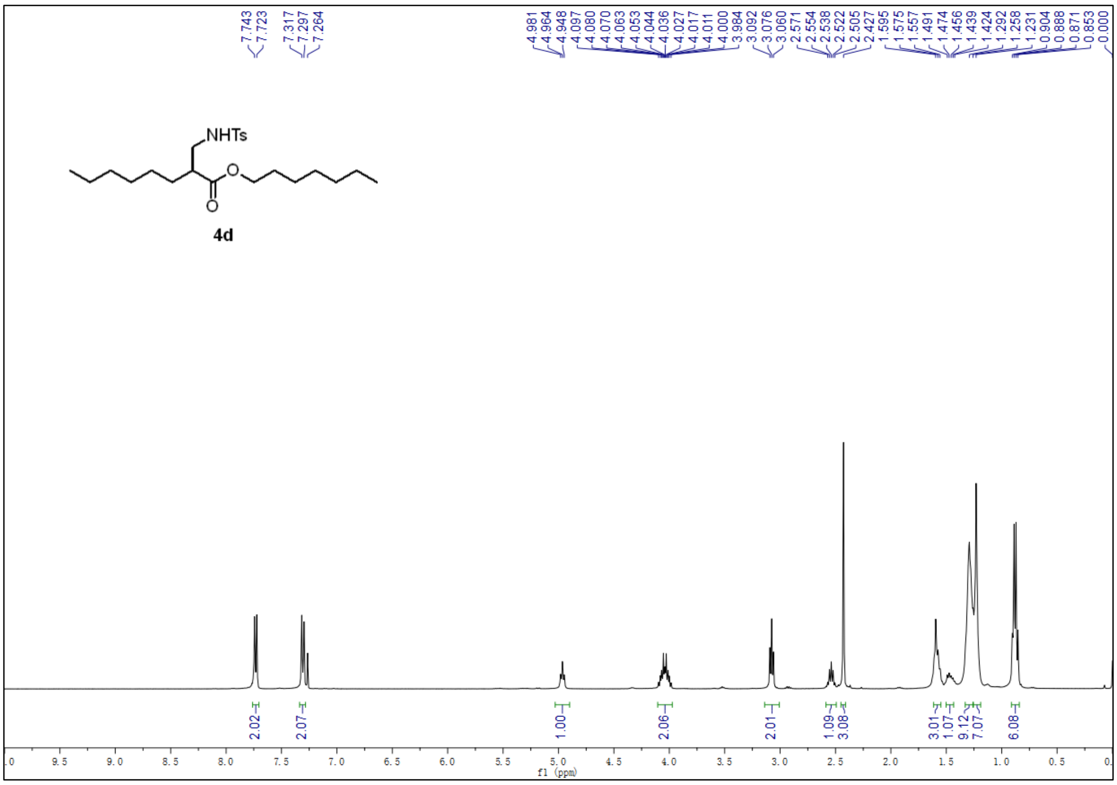

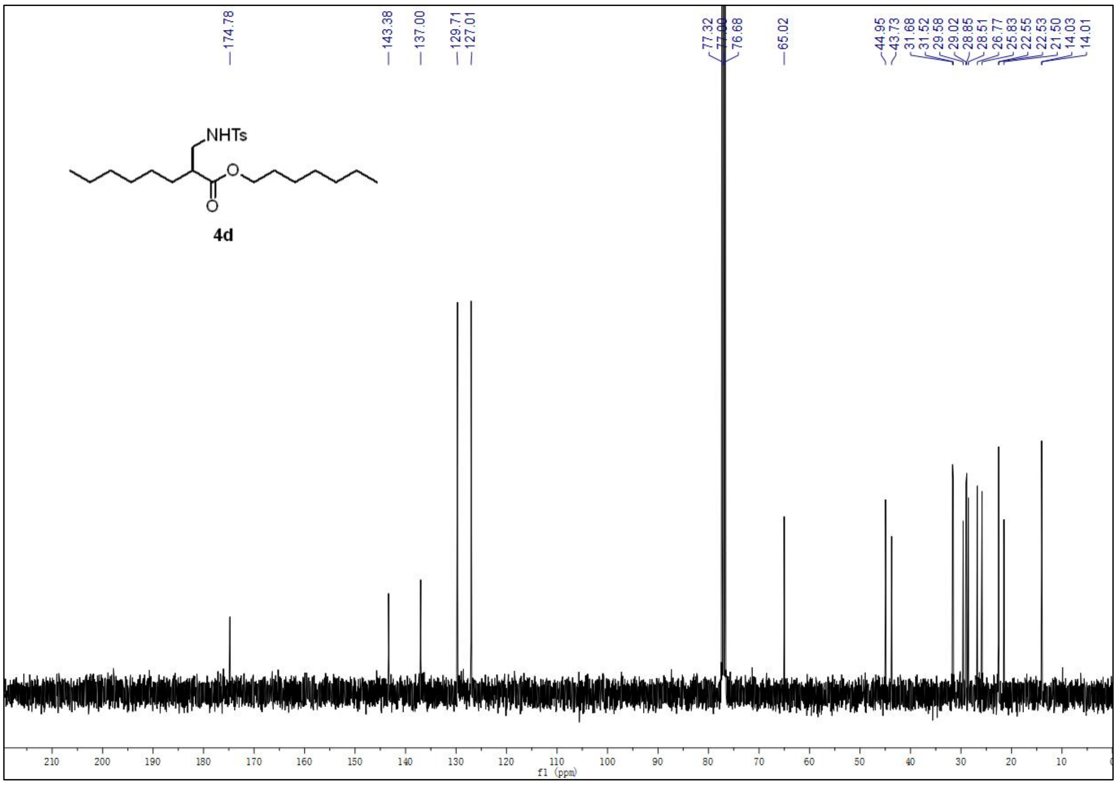

^{1}H NMR (400 MHz, CDCl3) and ^{13}C NMR (101 MHz, CDCl3) spectrum of 4e

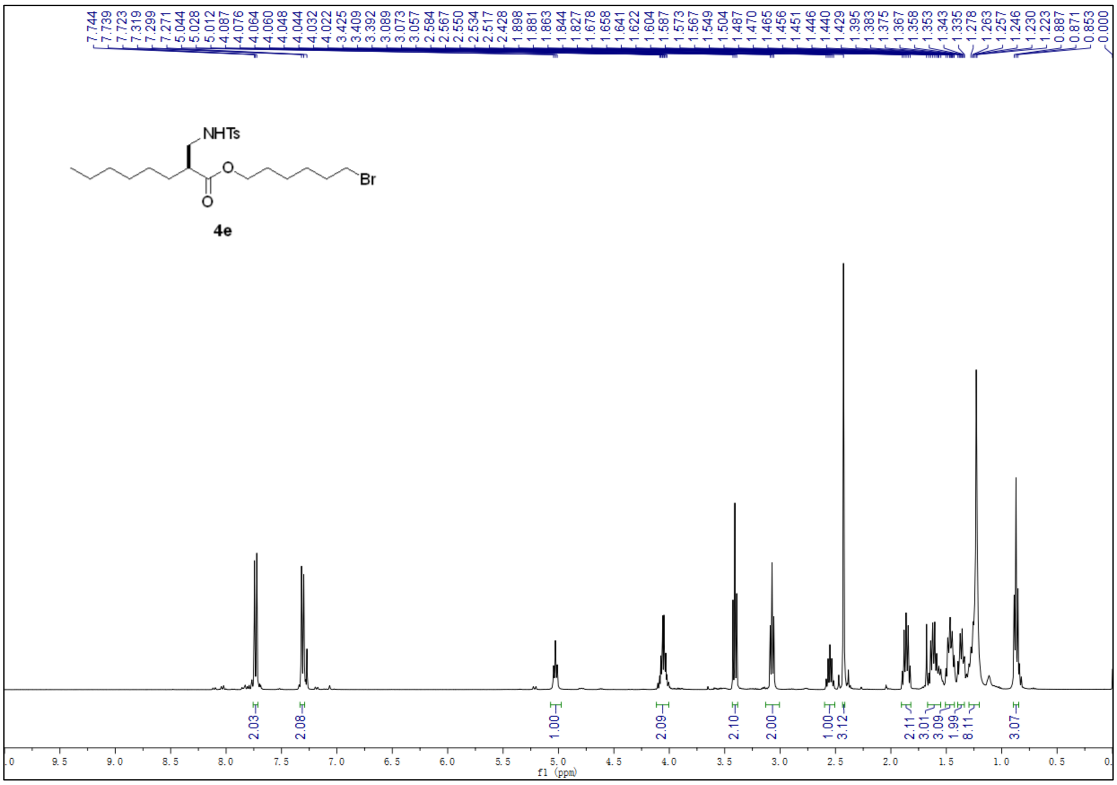

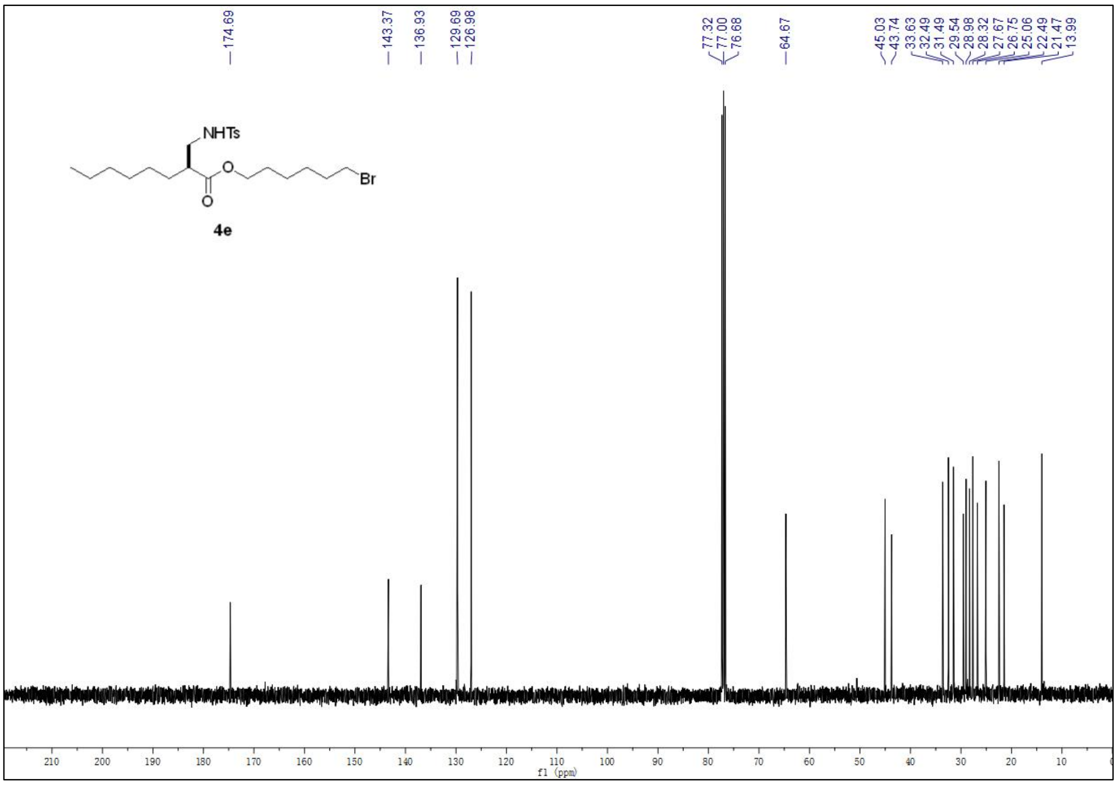

^{1}H NMR (400 MHz, $CDCl_3$) and ^{13}C NMR (101 MHz, $CDCl_3$) spectrum of 4f

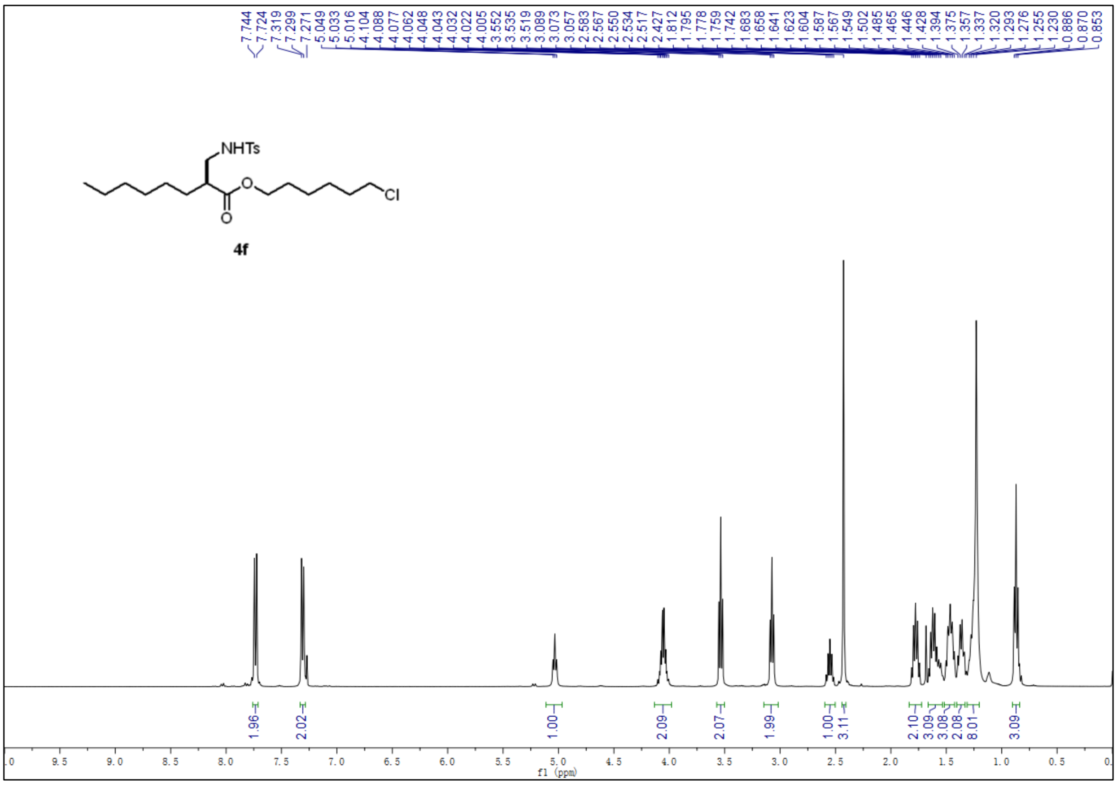

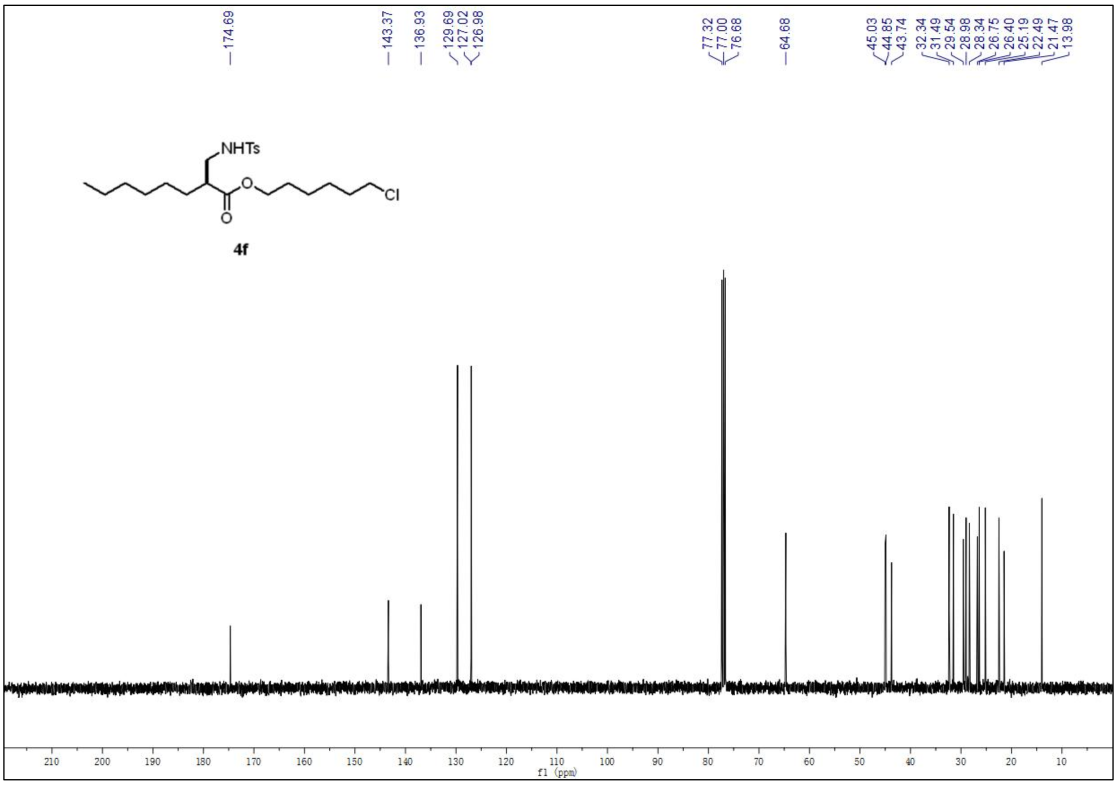

^{1}H NMR (400 MHz, $CDCl_3$) and ^{13}C NMR (101 MHz, $CDCl_3$) spectrum of 4g

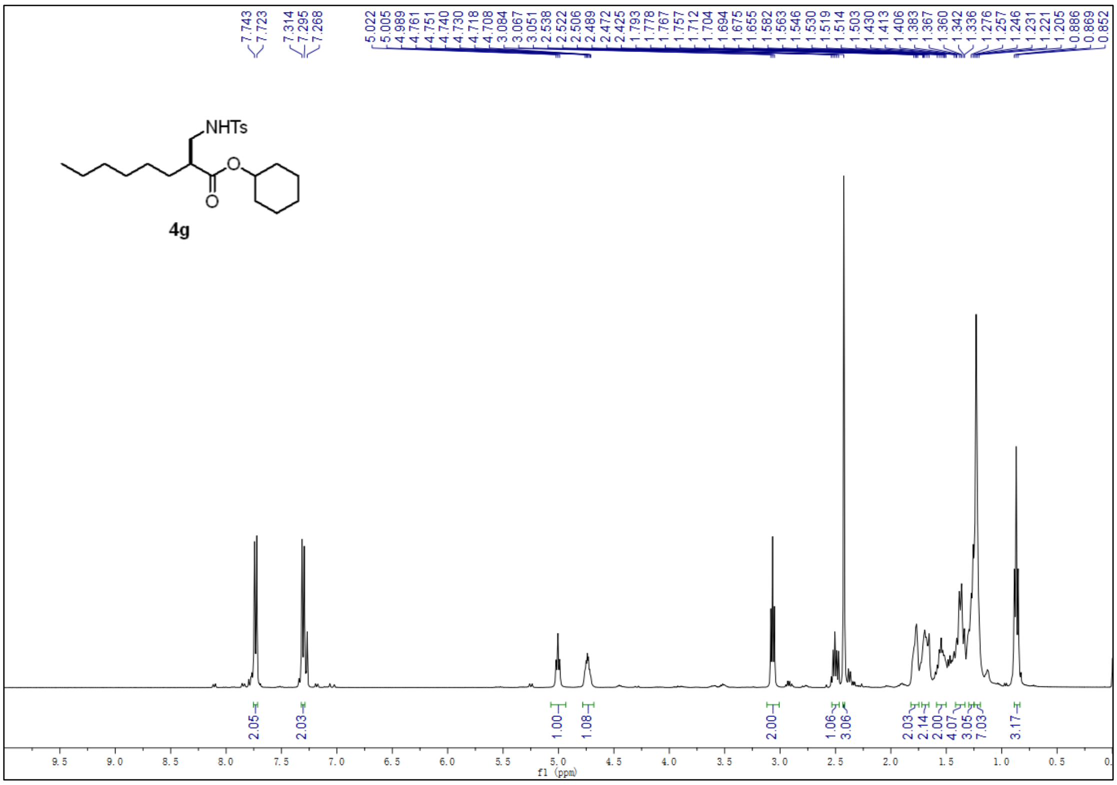

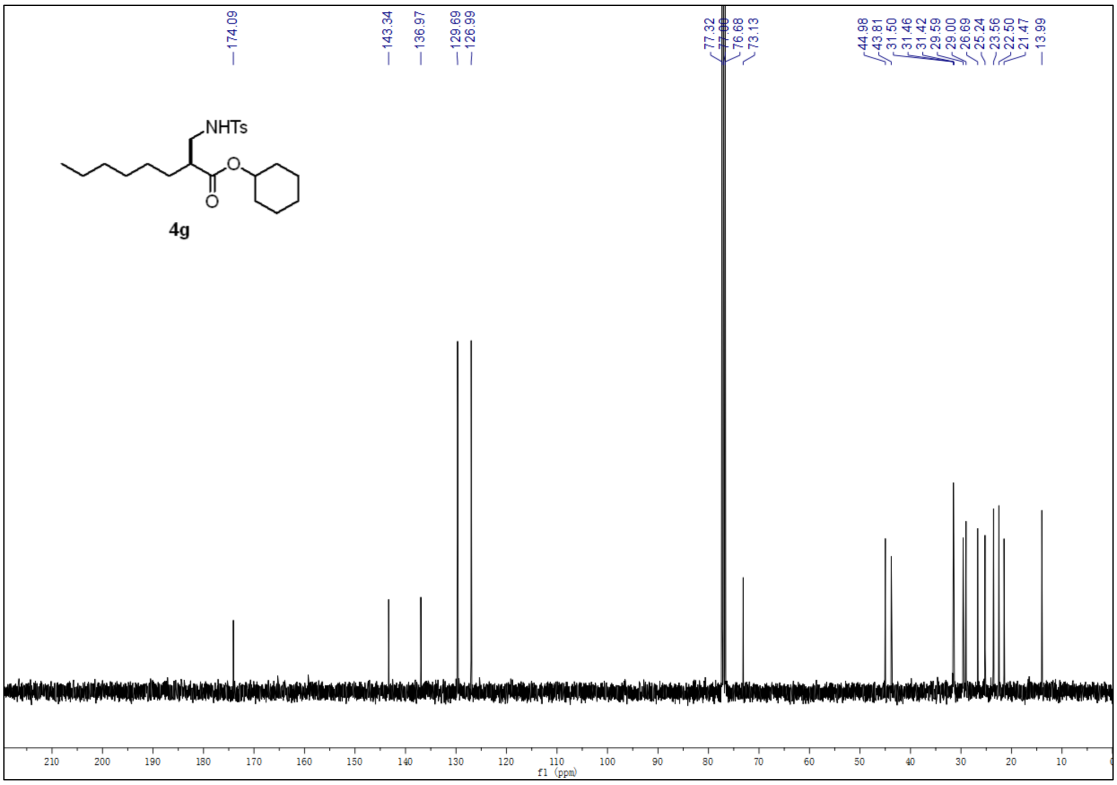

^{1}H NMR (400 MHz, $CDCl_3$) and ^{13}C NMR (101 MHz, $CDCl_3$) spectrum of 4h

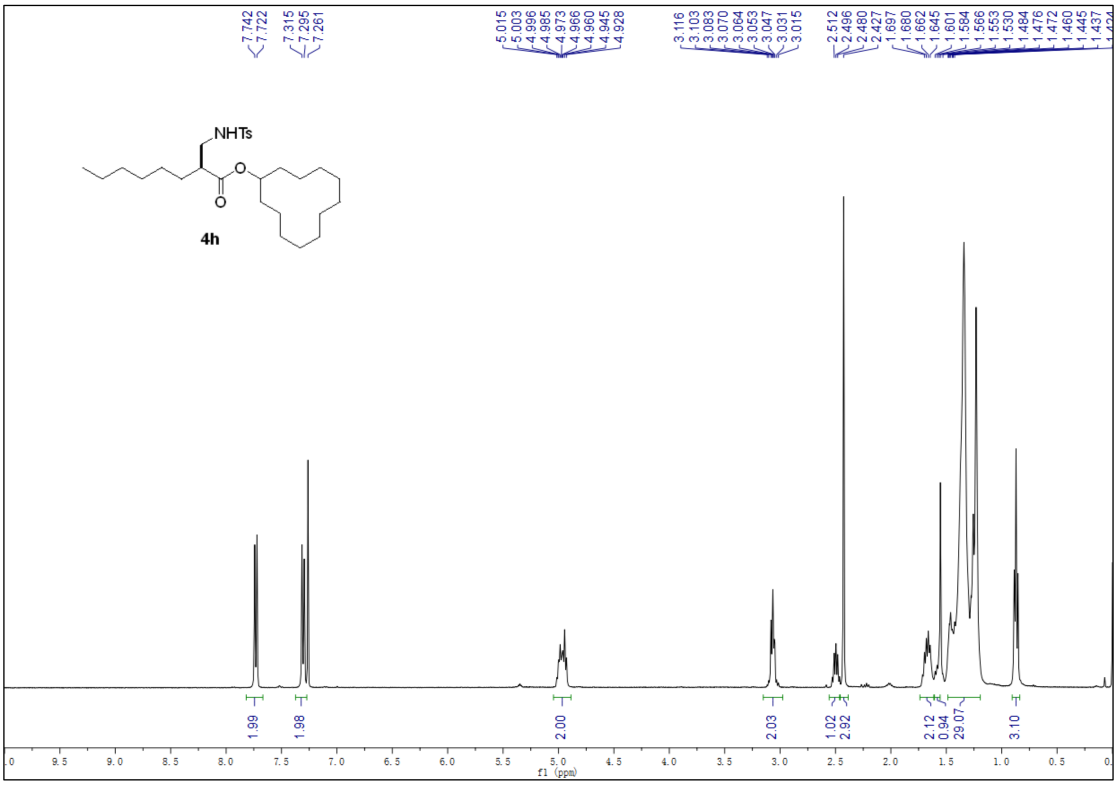

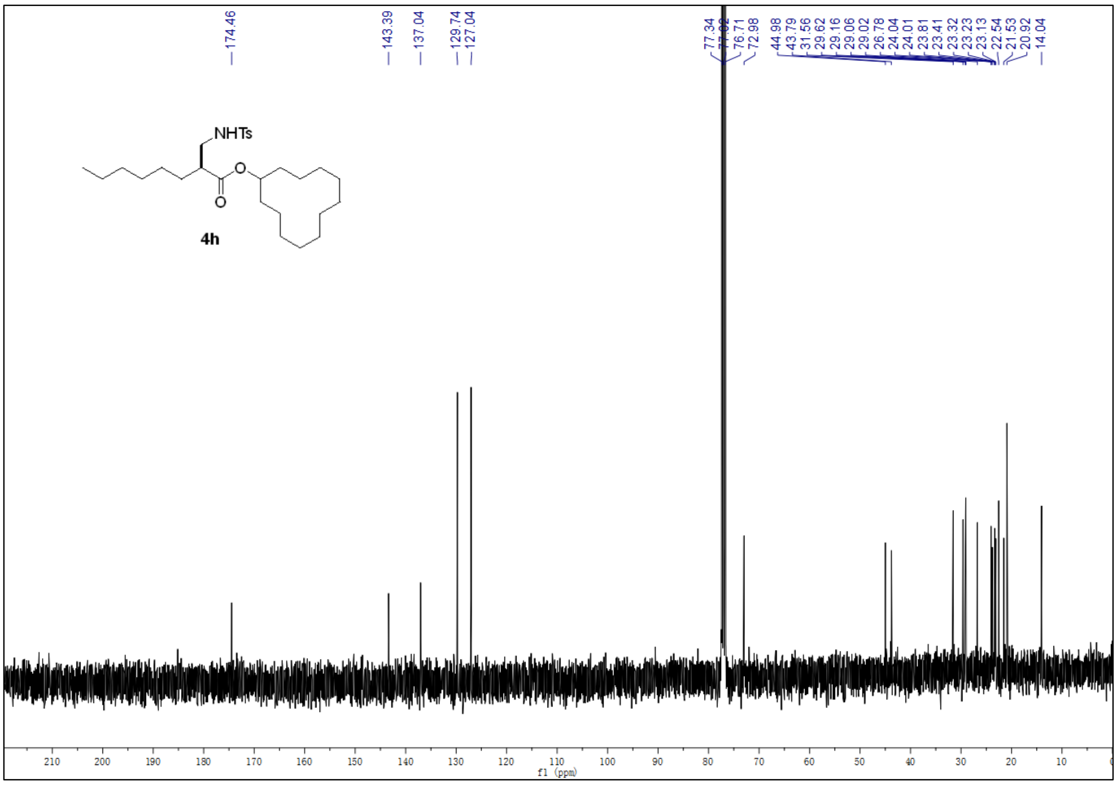

^{1}H NMR (400 MHz, $CDCl_3$) and ^{13}C NMR (101 MHz, $CDCl_3$) spectrum of 4i

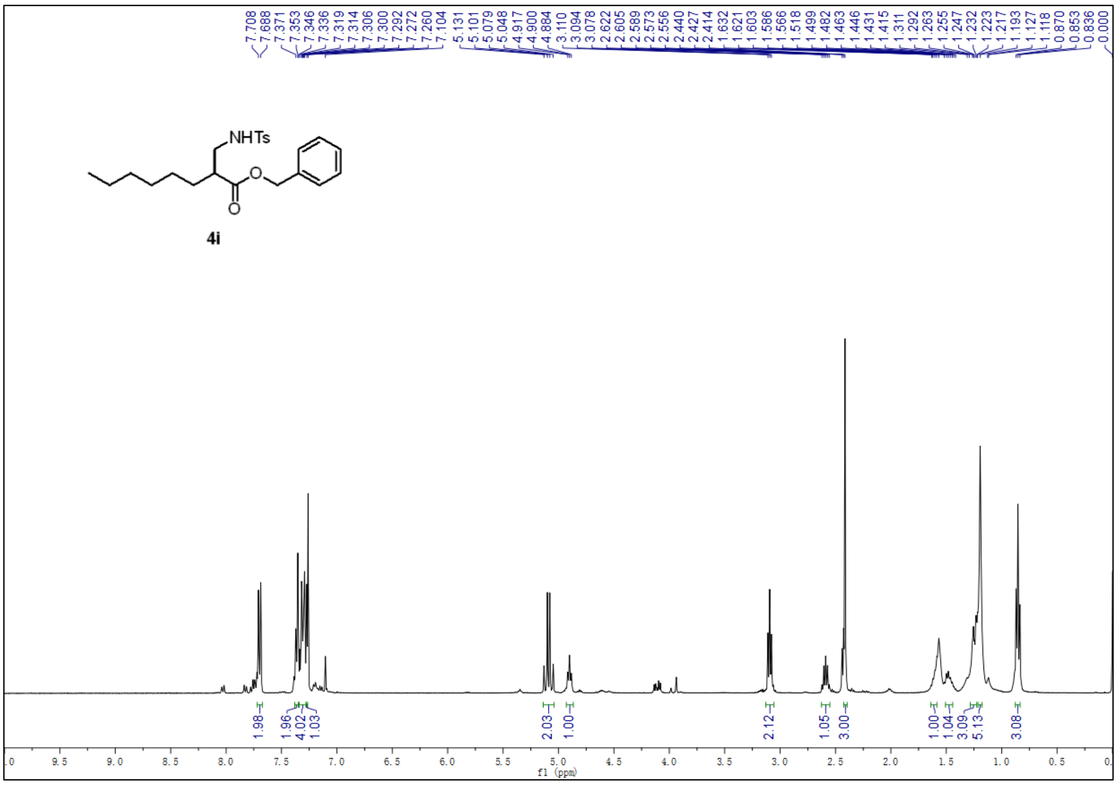

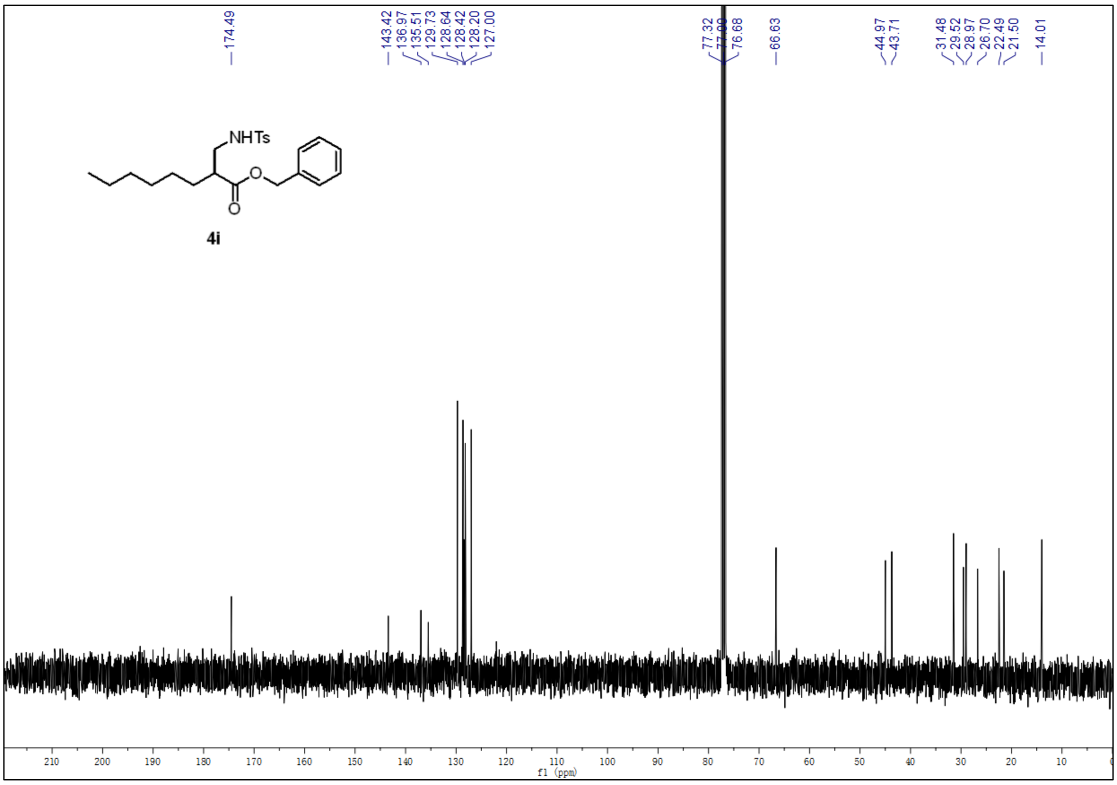

^{1}H NMR (400 MHz, $CDCl_3$), ^{13}C NMR (101 MHz, $CDCl_3$) and ^{19}F NMR (376 MHz, $CDCl_3$) spectrum of 4j

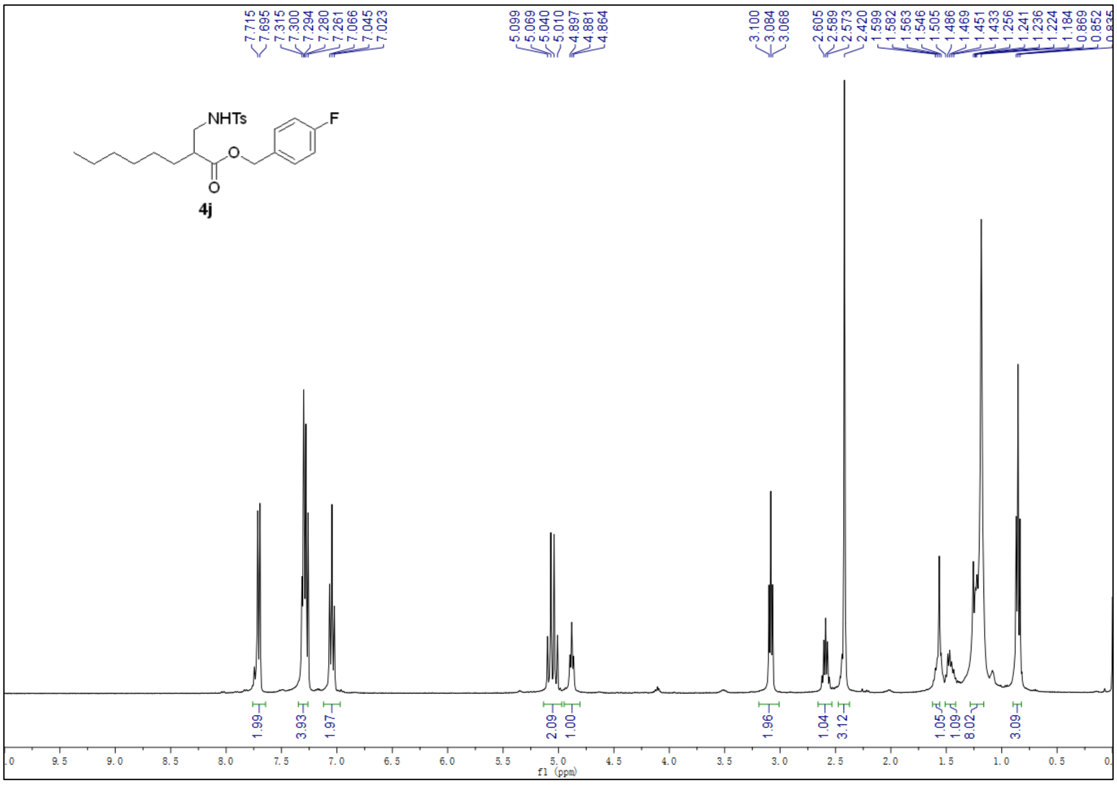

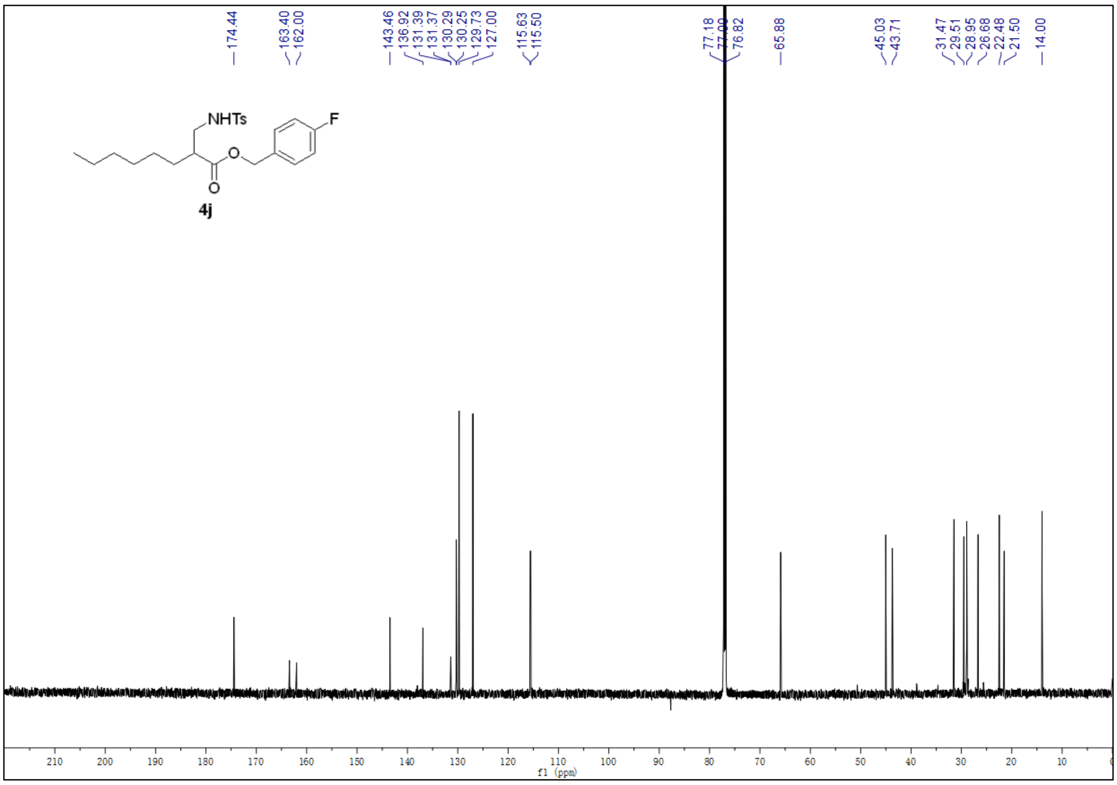

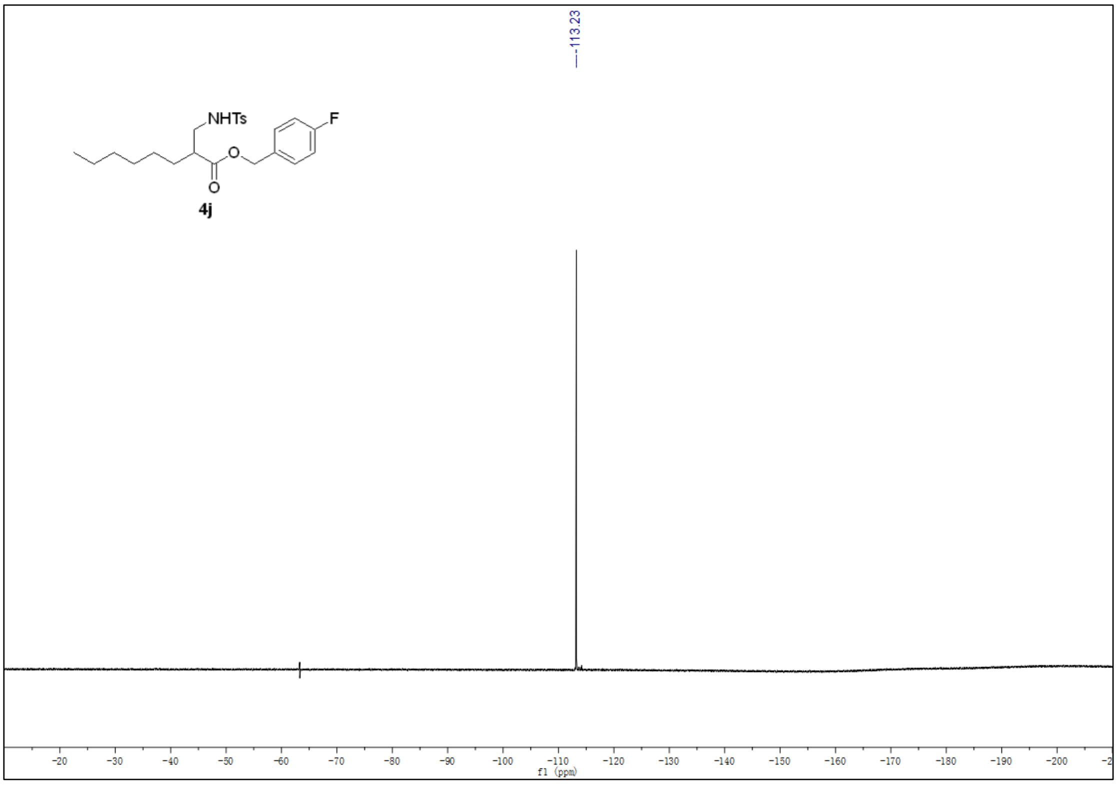

^{1}H NMR (400 MHz, CDCl$_3$), ^{13}C NMR (101 MHz, CDCl$_3$) and ^{19}F NMR (376 MHz, CDCl$_3$) spectrum of 4k

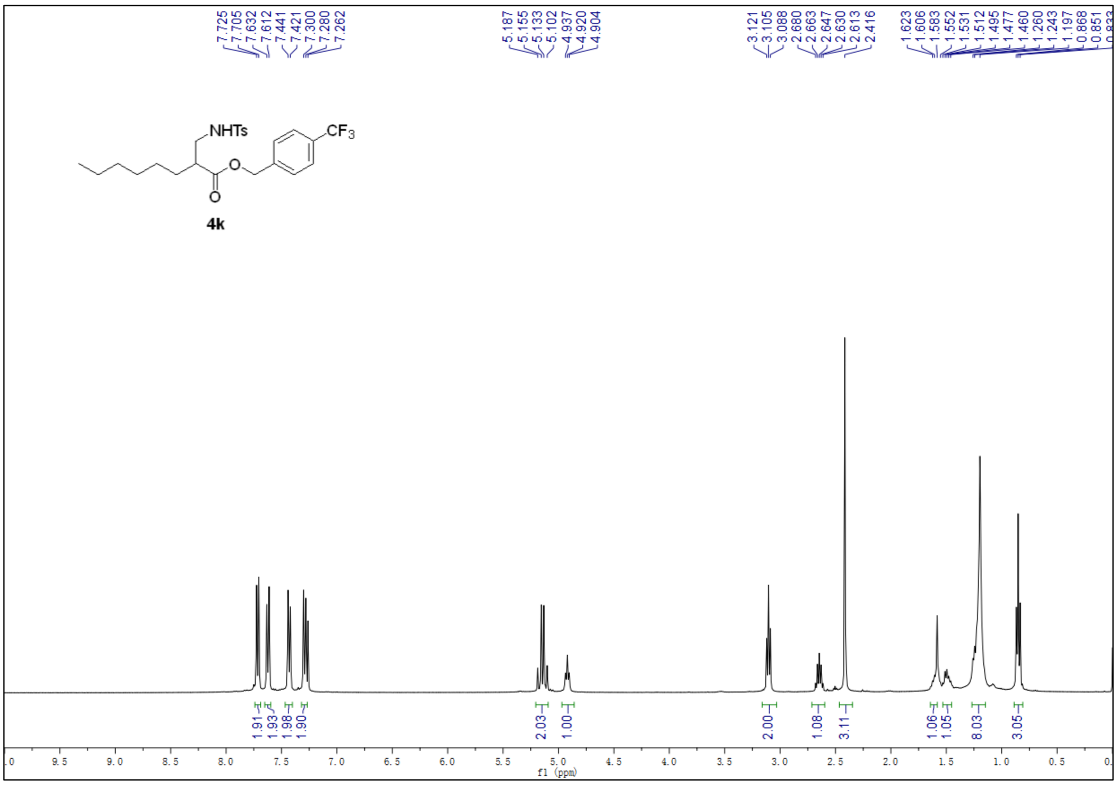

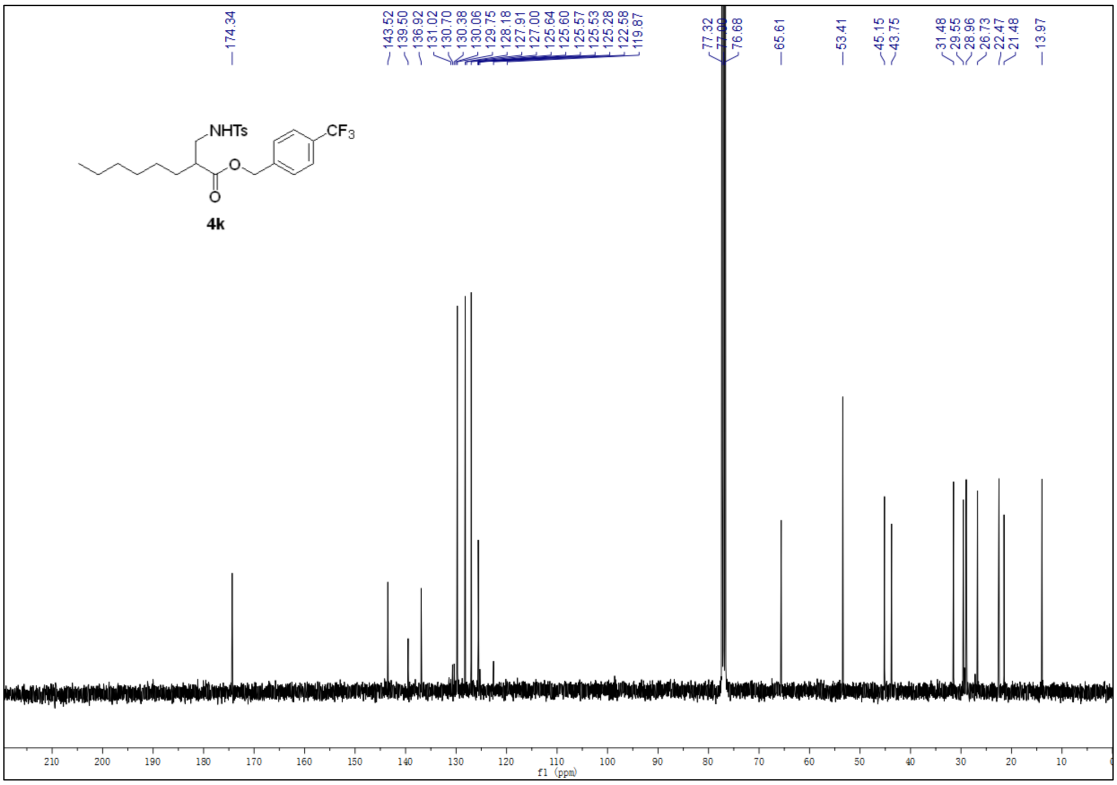

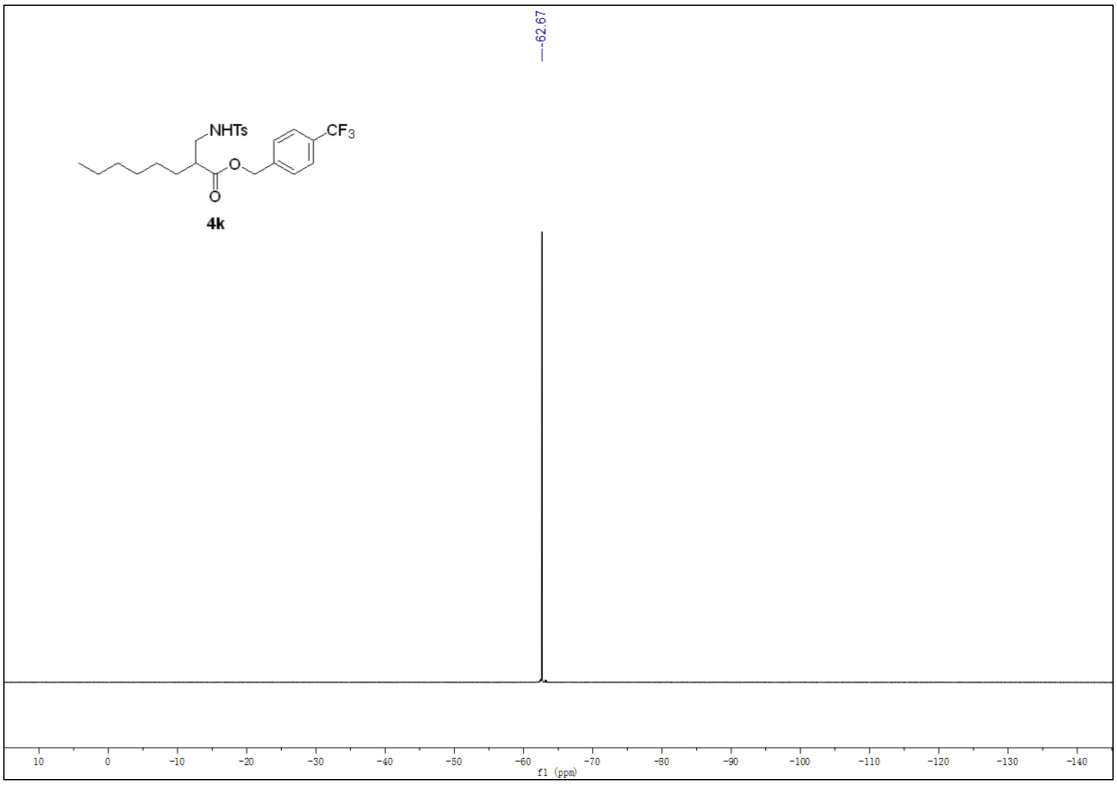

^{1}H NMR (400 MHz, $CDCl_3$) and ^{13}C NMR (101 MHz, $CDCl_3$) spectrum of 4l

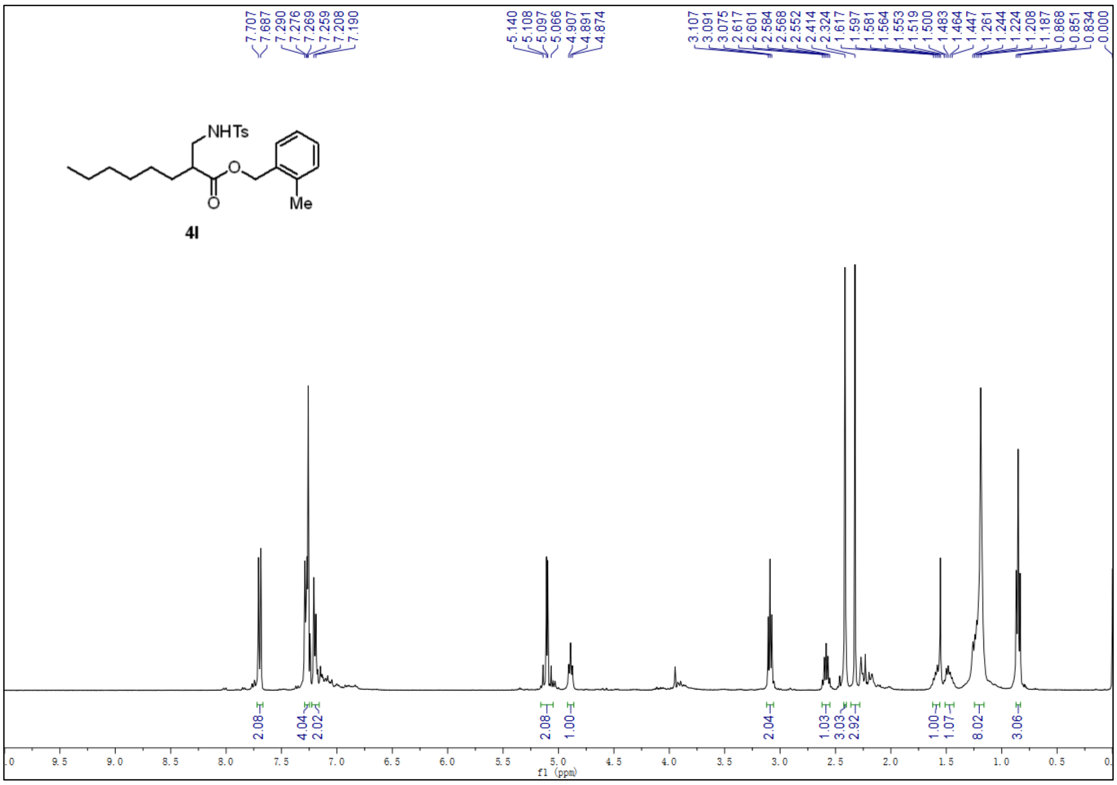

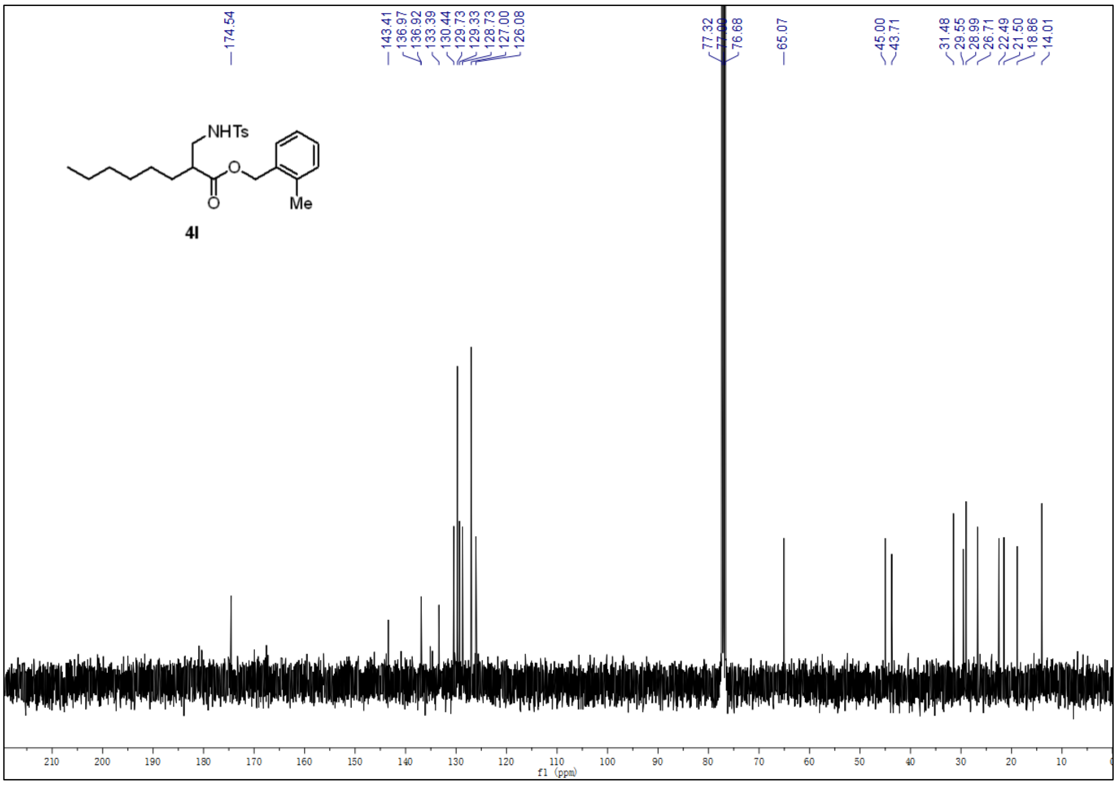

^{1}H NMR (400 MHz, CDCl$_3$) and ^{13}C NMR (101 MHz, CDCl$_3$) spectrum of 4m

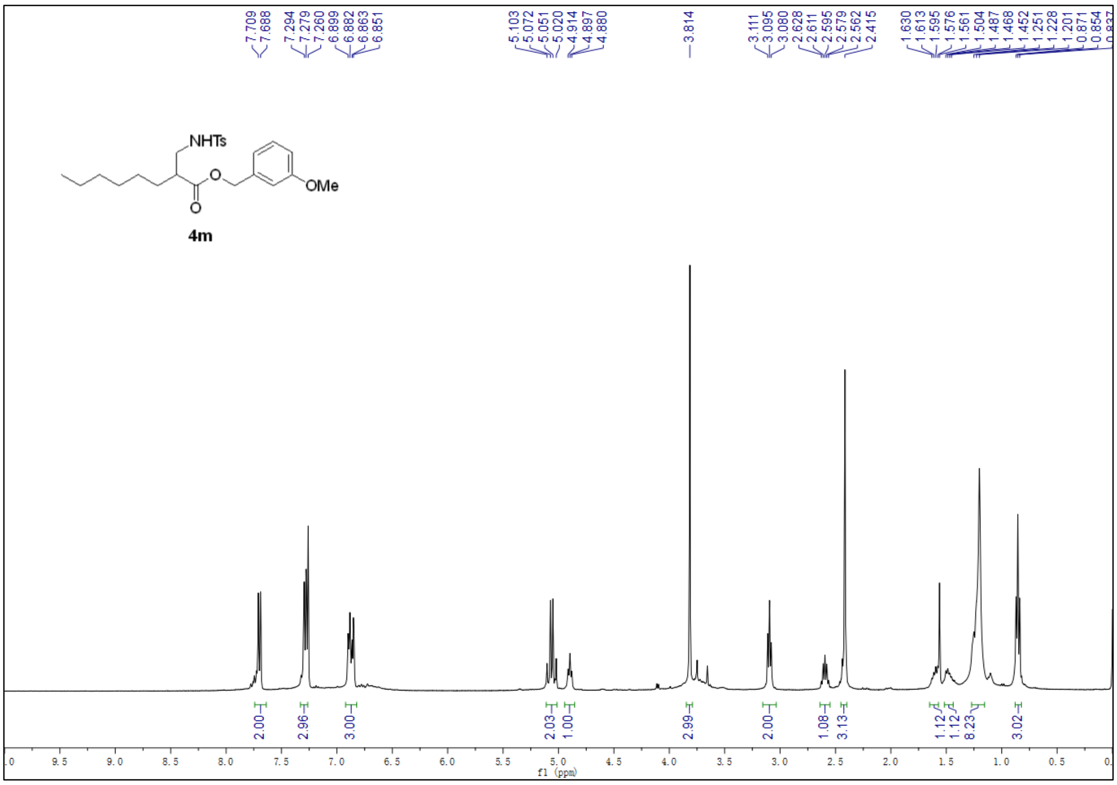

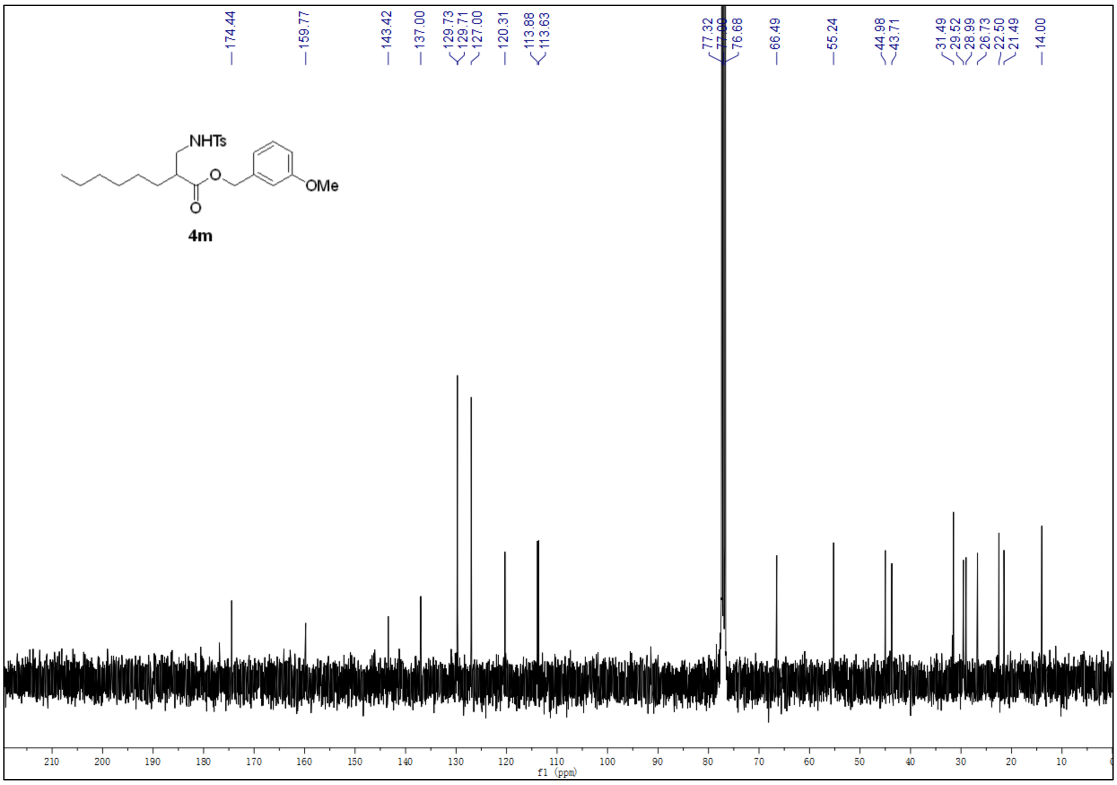

^1H NMR (400 MHz, $CDCl_3$) and ^{13}C NMR (101 MHz, $CDCl_3$) spectrum of 4n

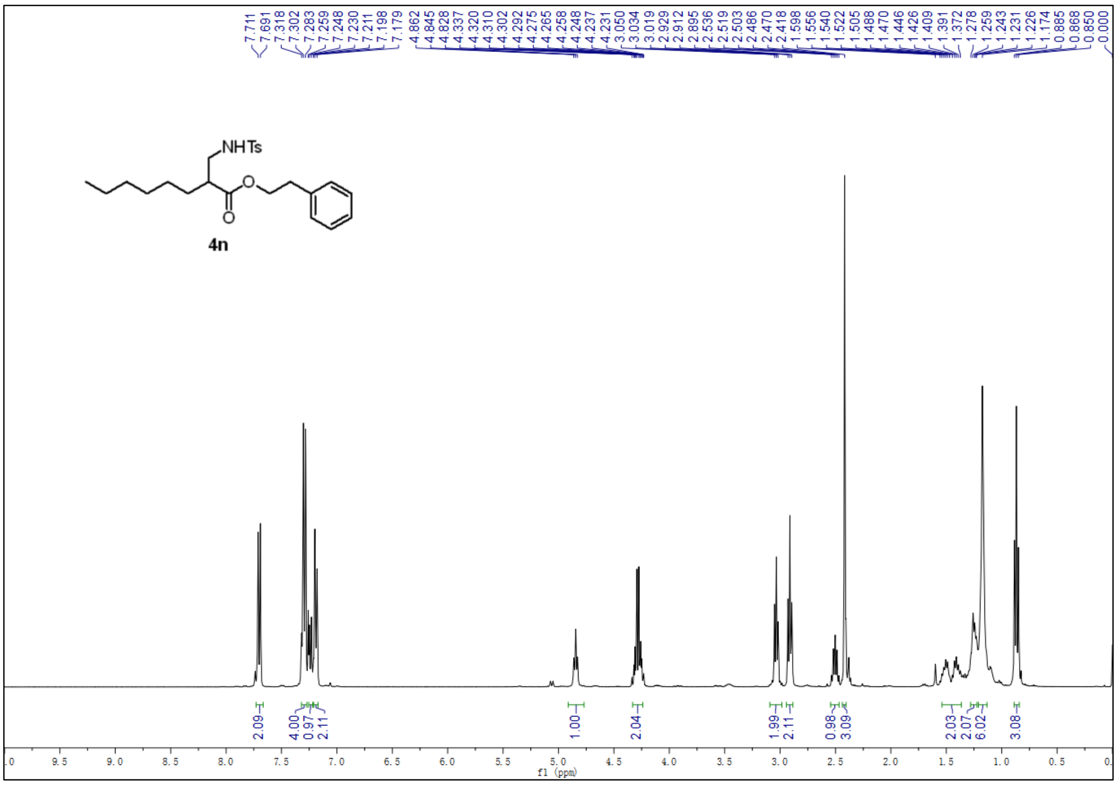

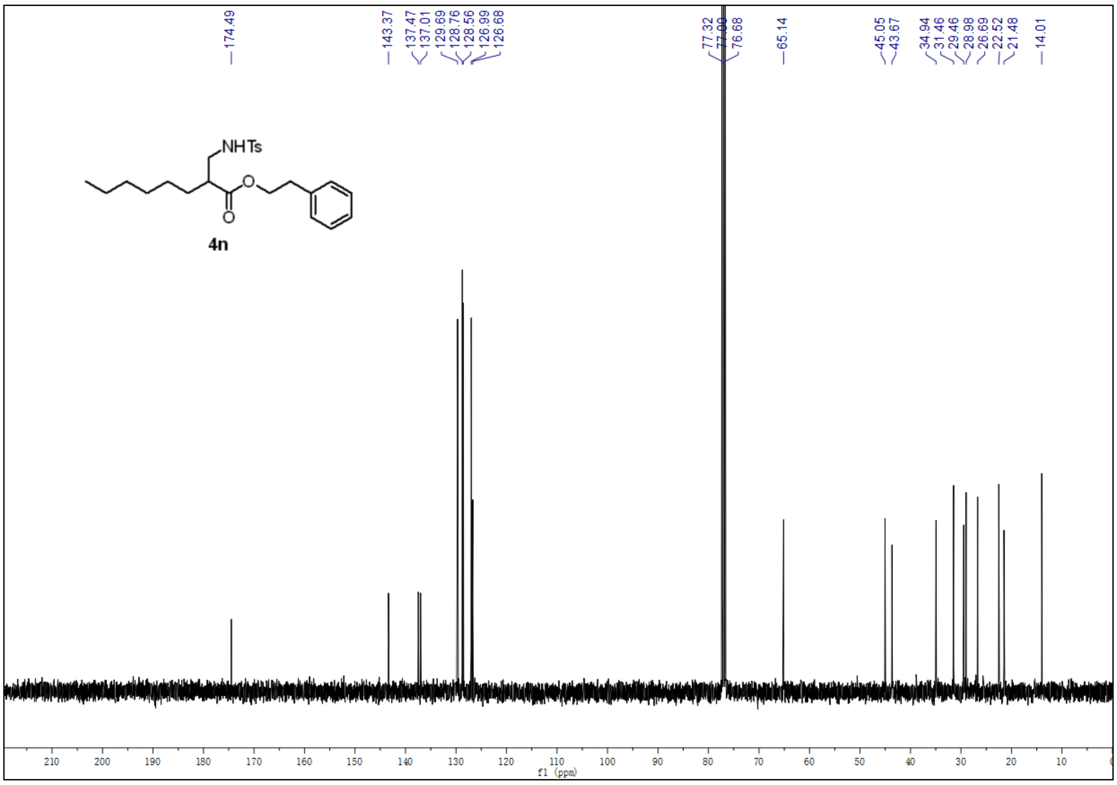

^{1}H NMR (400 MHz, CDCl3) and ^{13}C NMR (101 MHz, CDCl3) spectrum of 4o

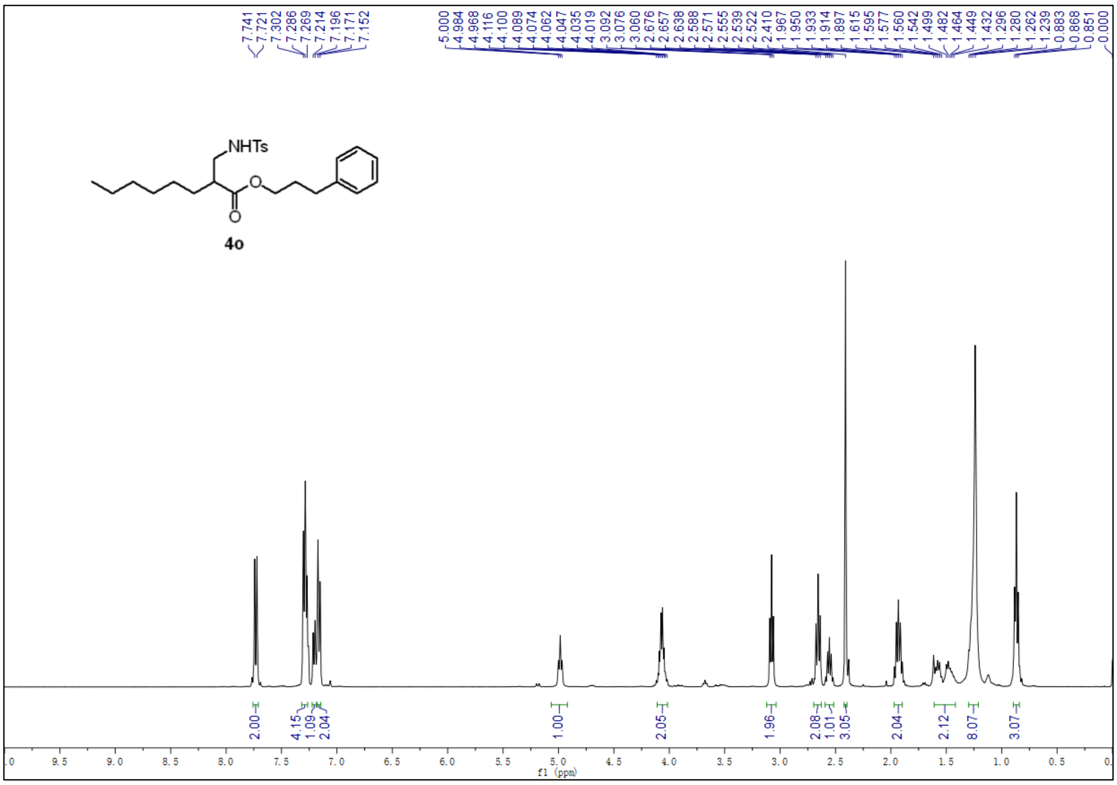

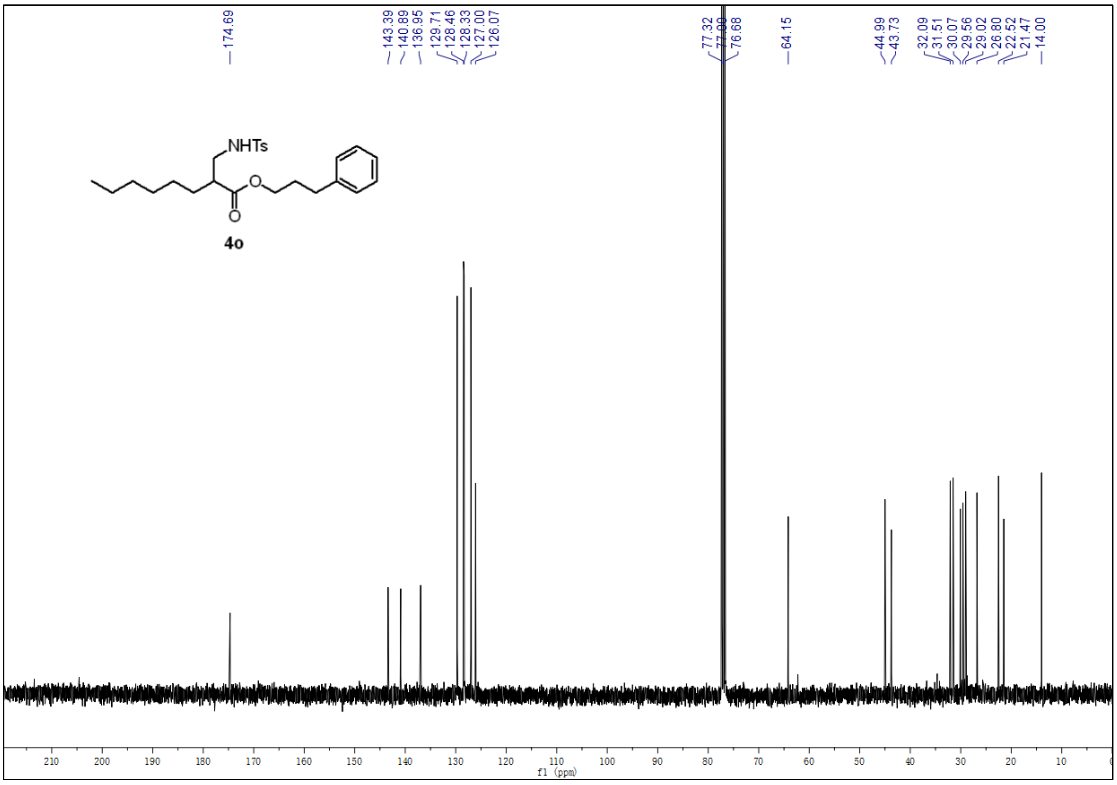

^{1}H NMR (400 MHz, CDCl3) and ^{13}C NMR (101 MHz, CDCl3) spectrum of 6a

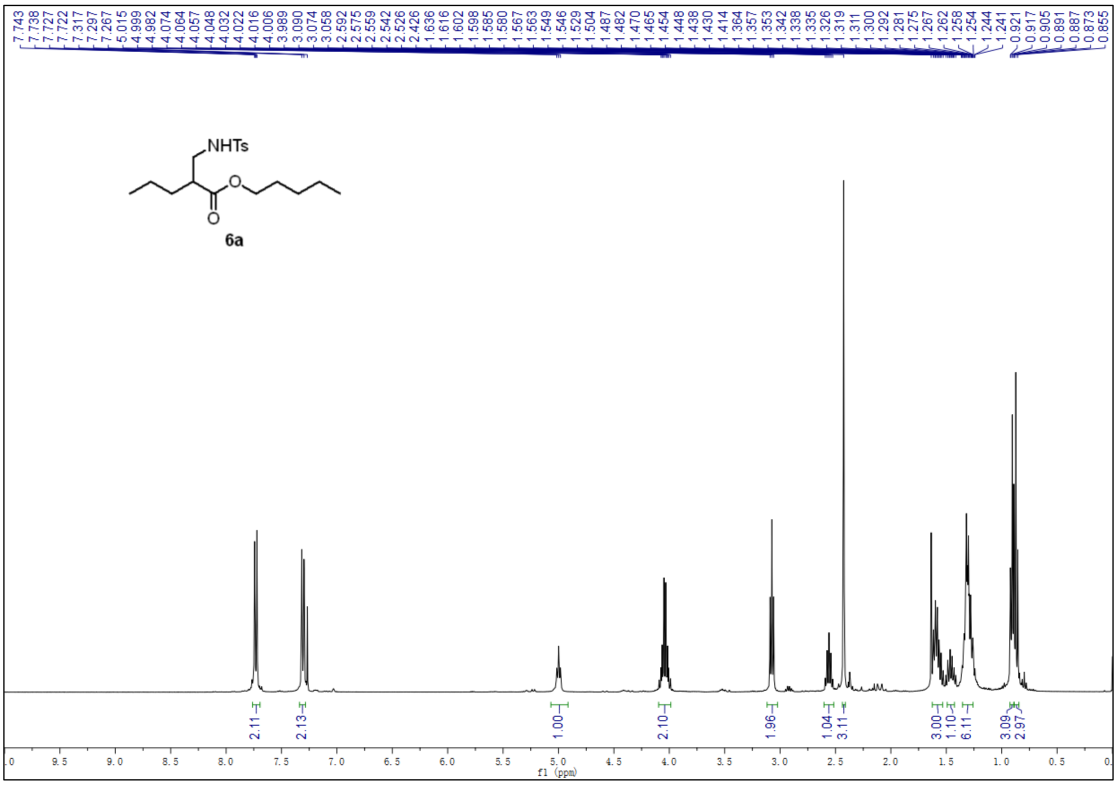

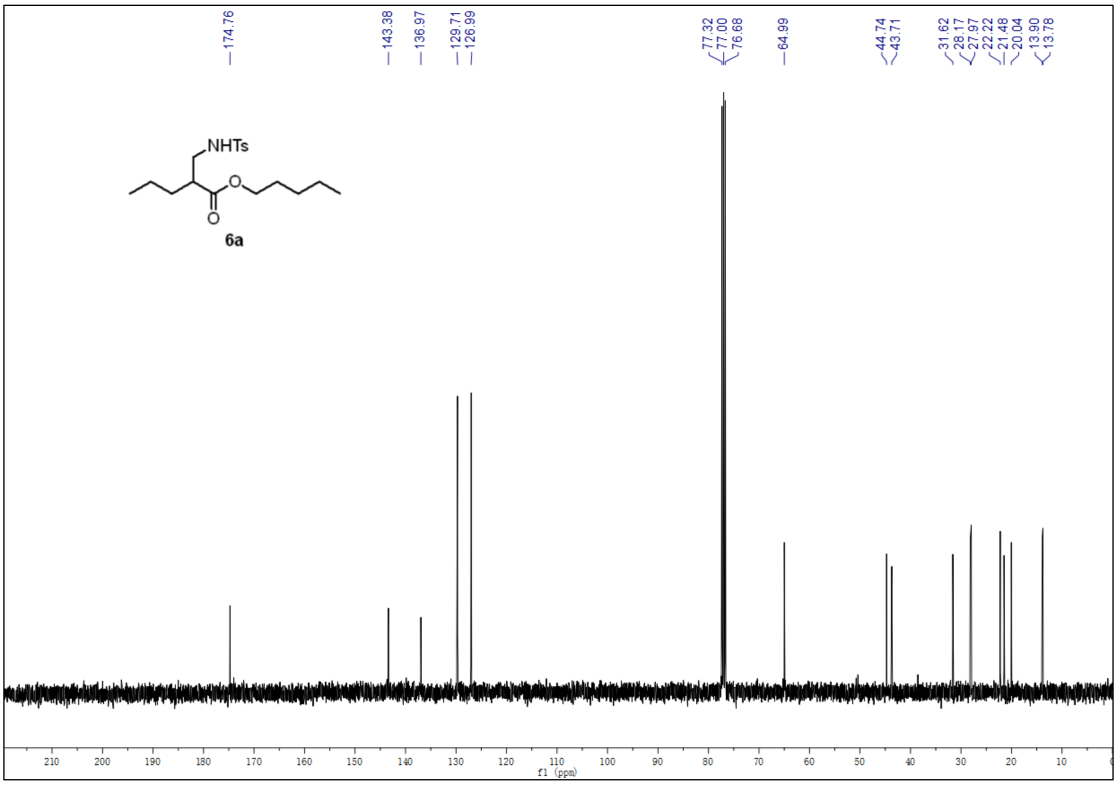

^{1}H NMR (400 MHz, $CDCl_3$) and ^{13}C NMR (101 MHz, $CDCl_3$) spectrum of 6b

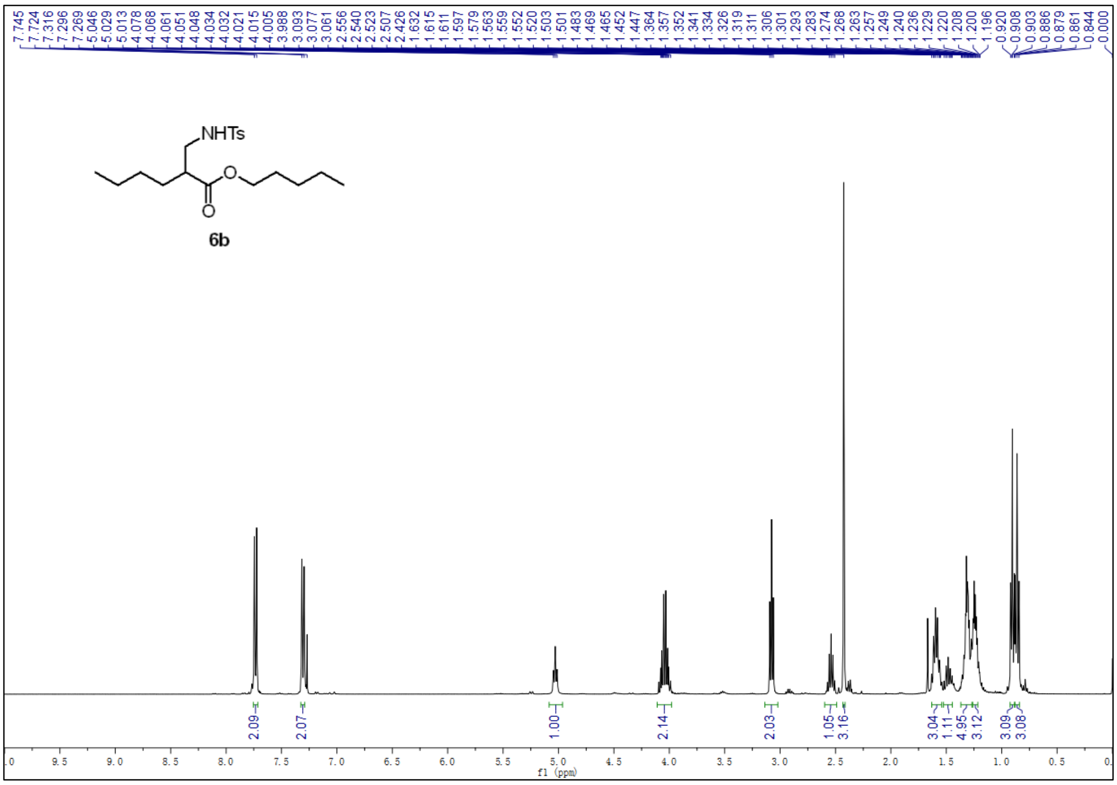

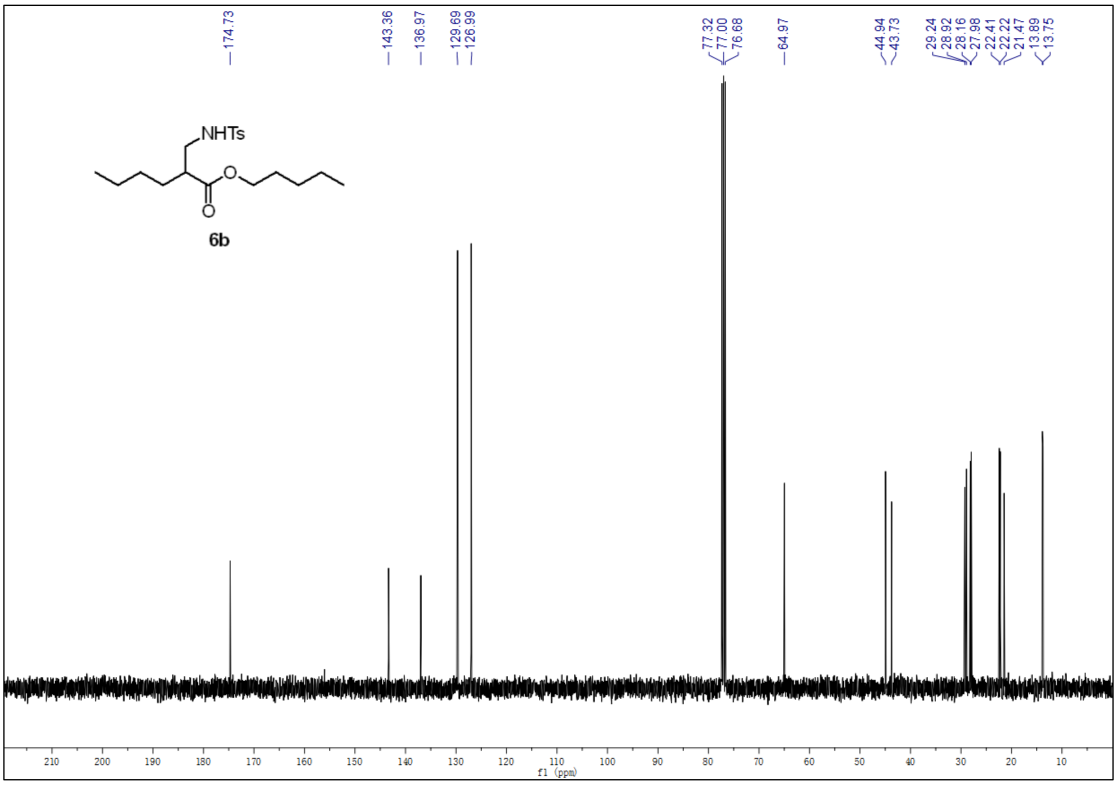

^{1}H NMR (400 MHz, $CDCl_3$) and ^{13}C NMR (101 MHz, $CDCl_3$) spectrum of 6c

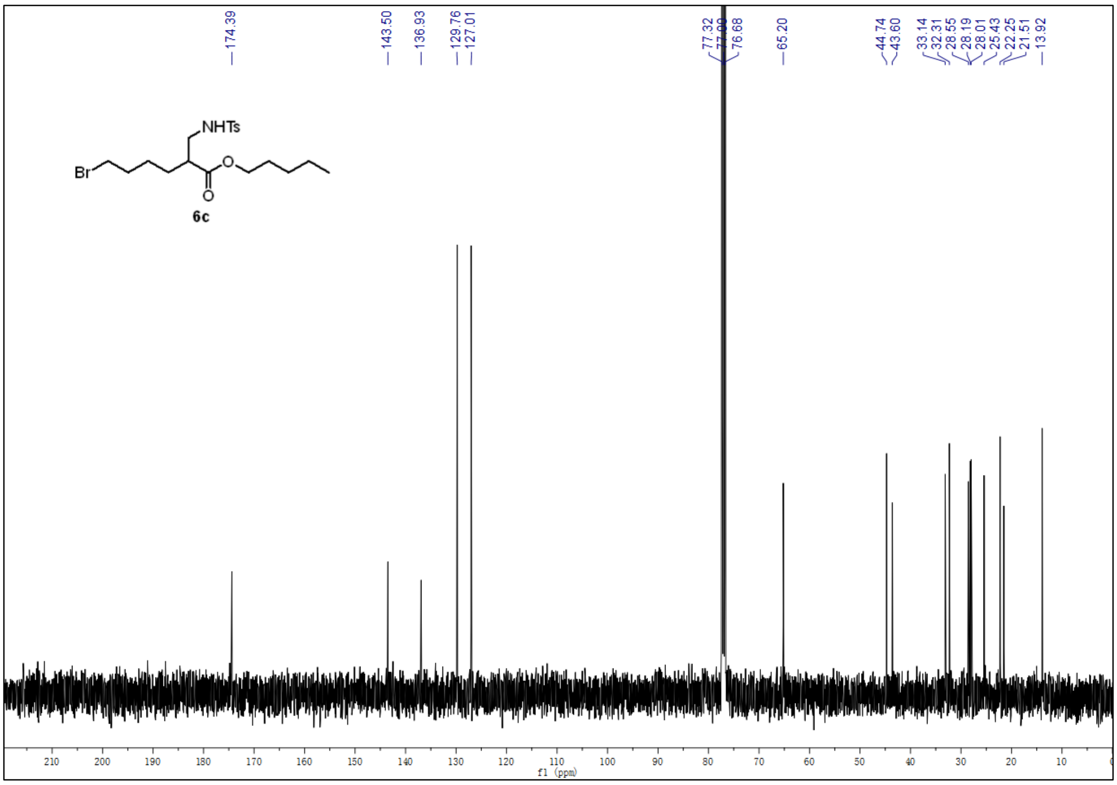

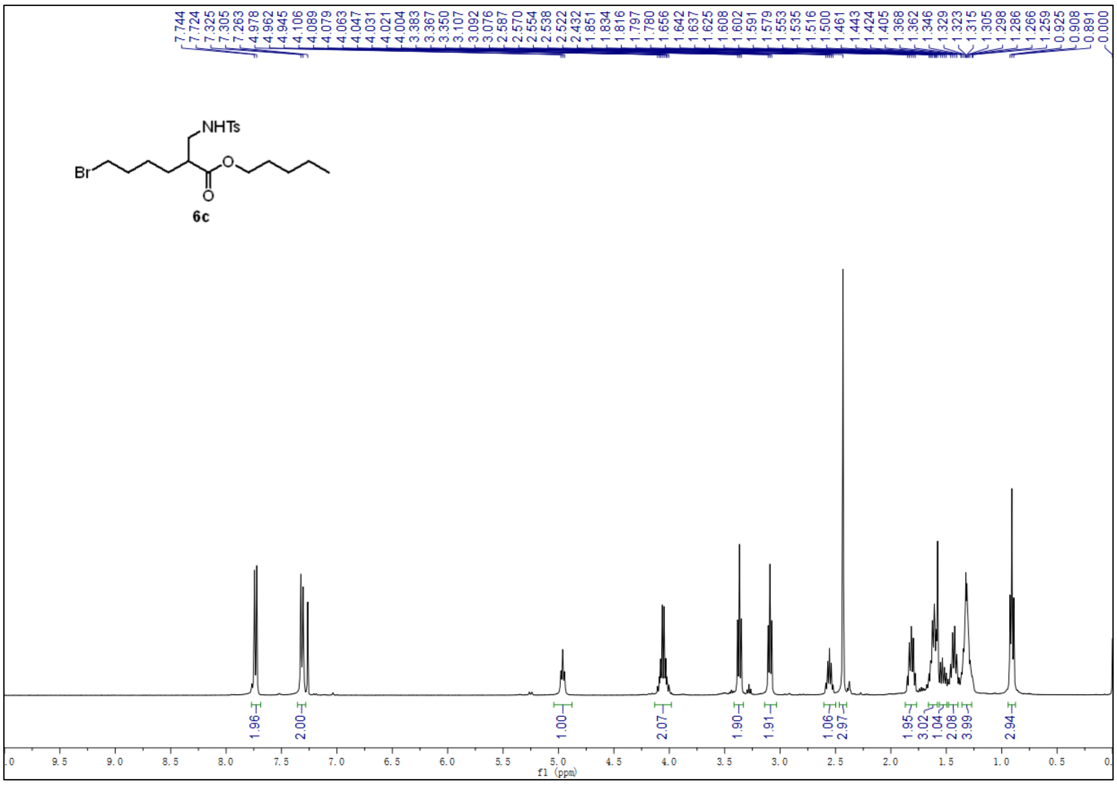

^{1}H NMR (400 MHz, CDCl₃) and ^{13}C NMR (101 MHz, CDCl₃) spectrum of 6d

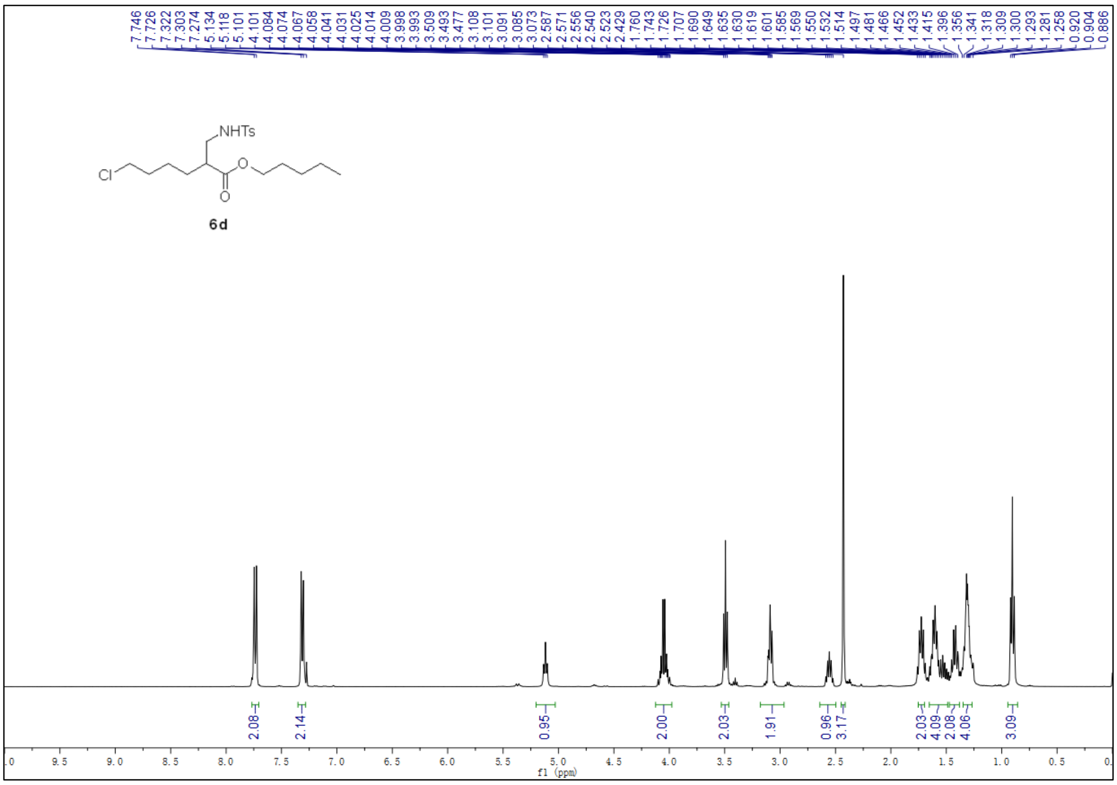

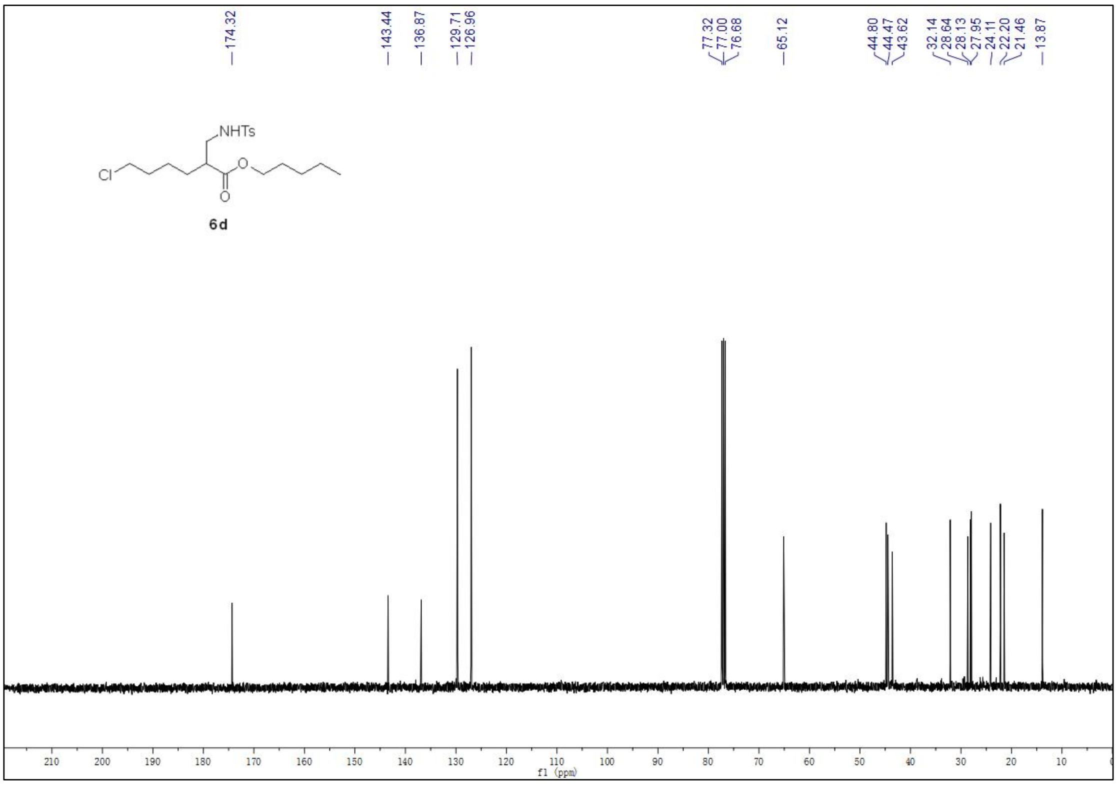

^{1}H NMR (400 MHz, CDCl3) and ^{13}C NMR (101 MHz, CDCl3) spectrum of 6e

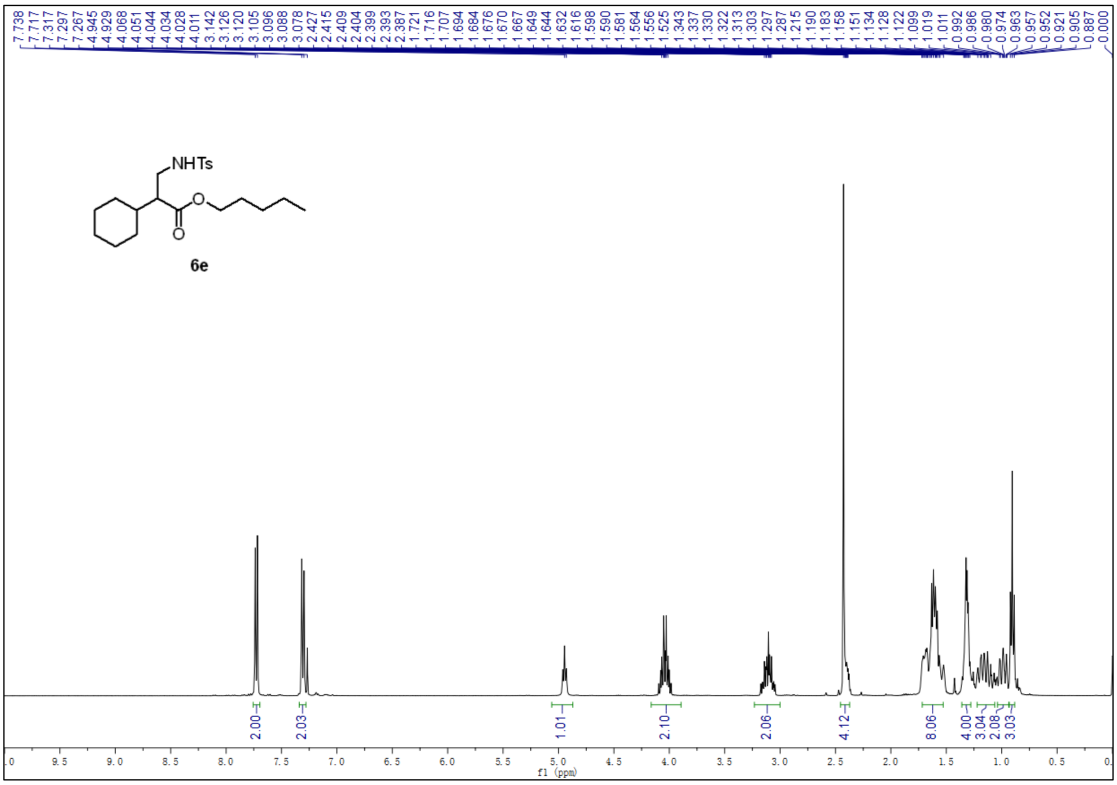

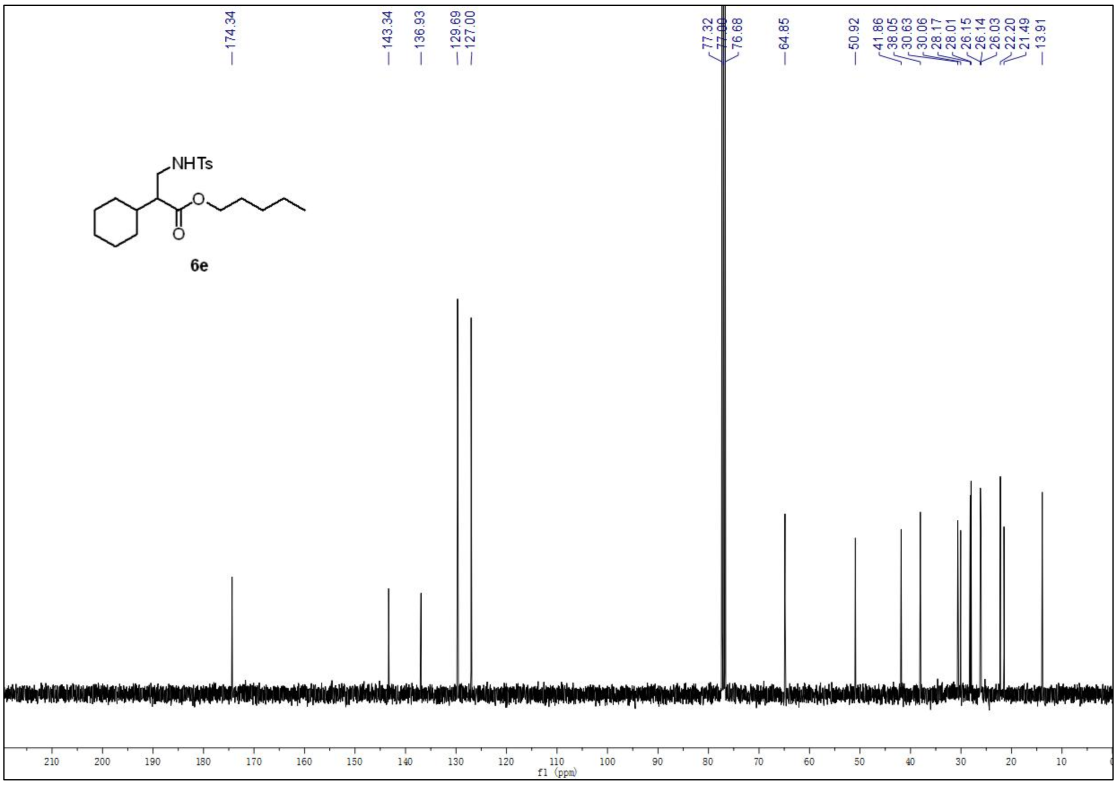

^{1}H NMR (400 MHz, $CDCl_3$) and ^{13}C NMR (101 MHz, $CDCl_3$) spectrum of 6f

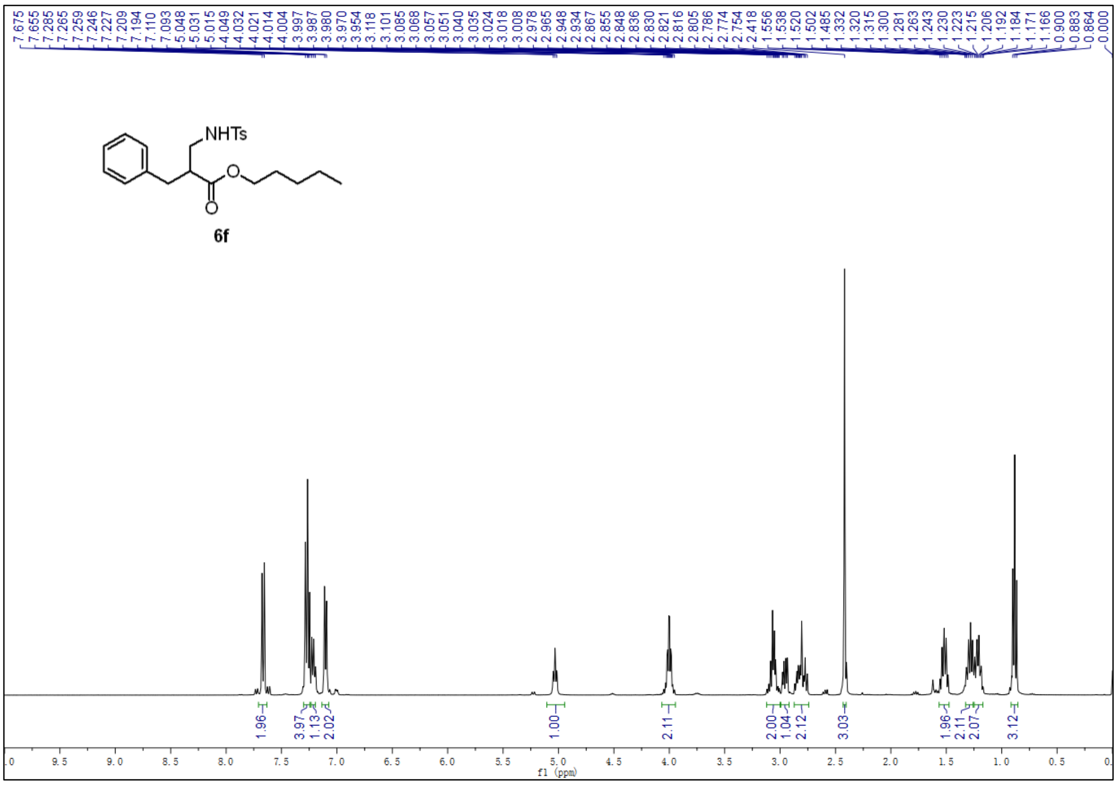

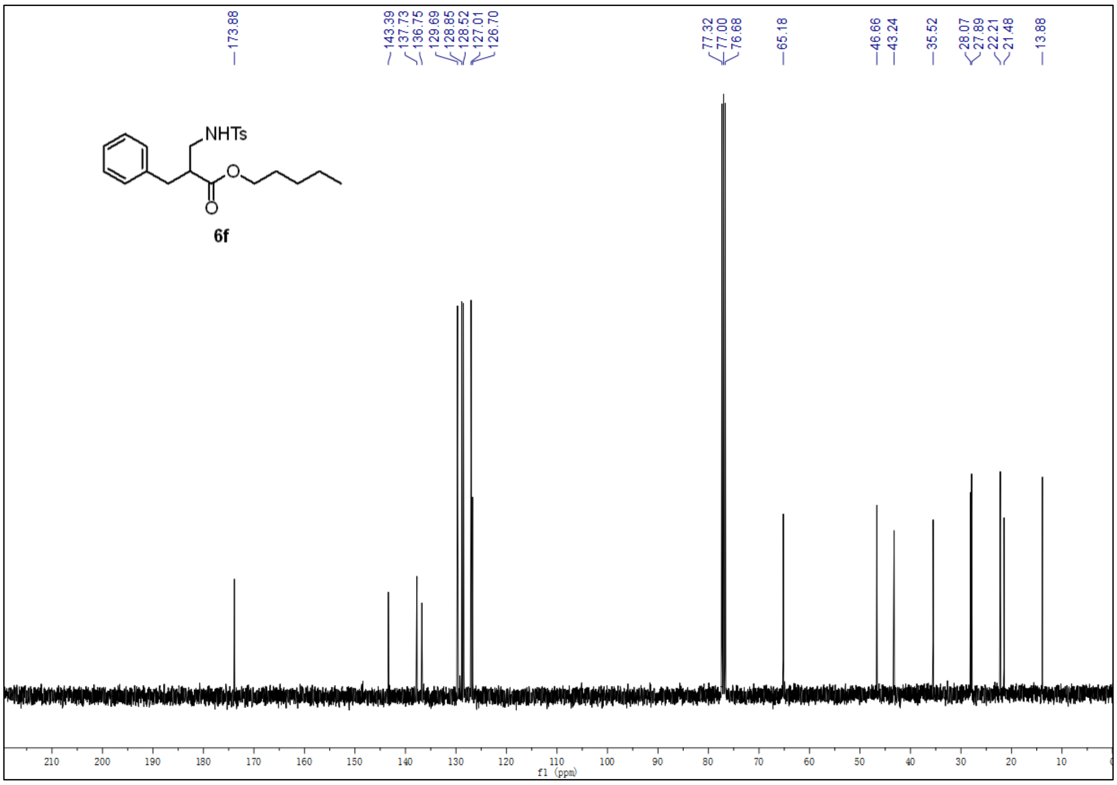

^{1}H NMR (400 MHz, CDCl3) and ^{13}C NMR (101 MHz, CDCl3) spectrum of 6g

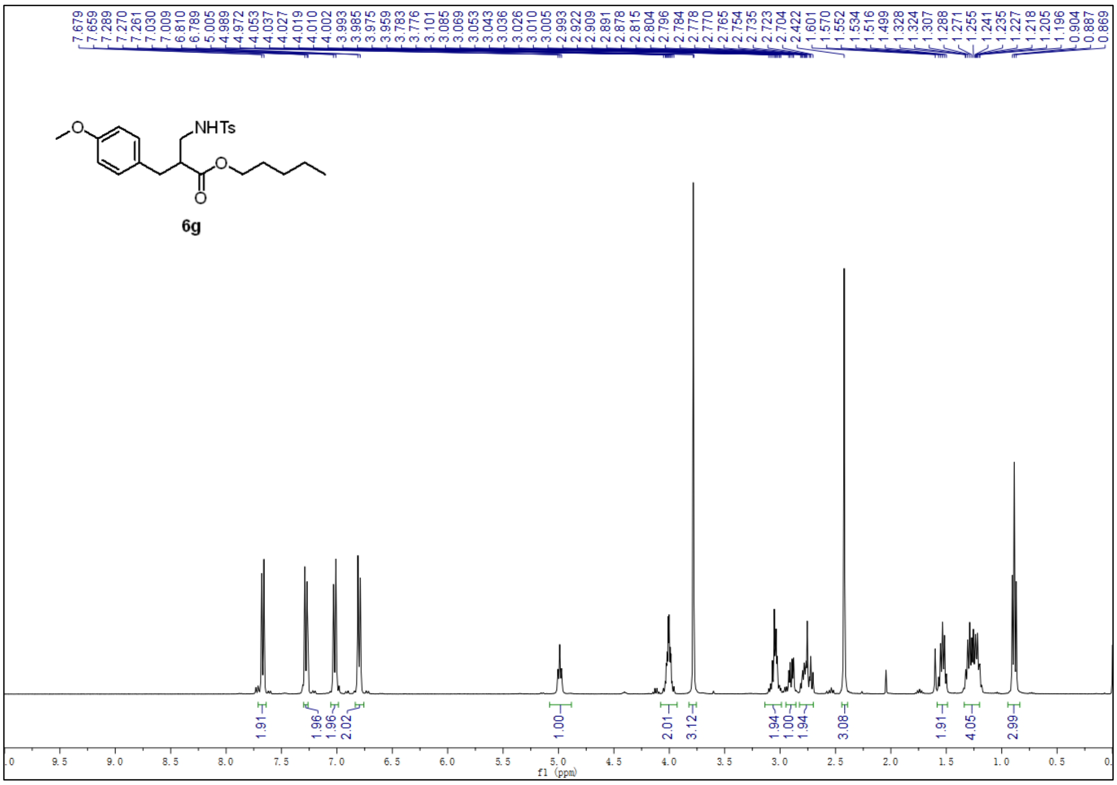

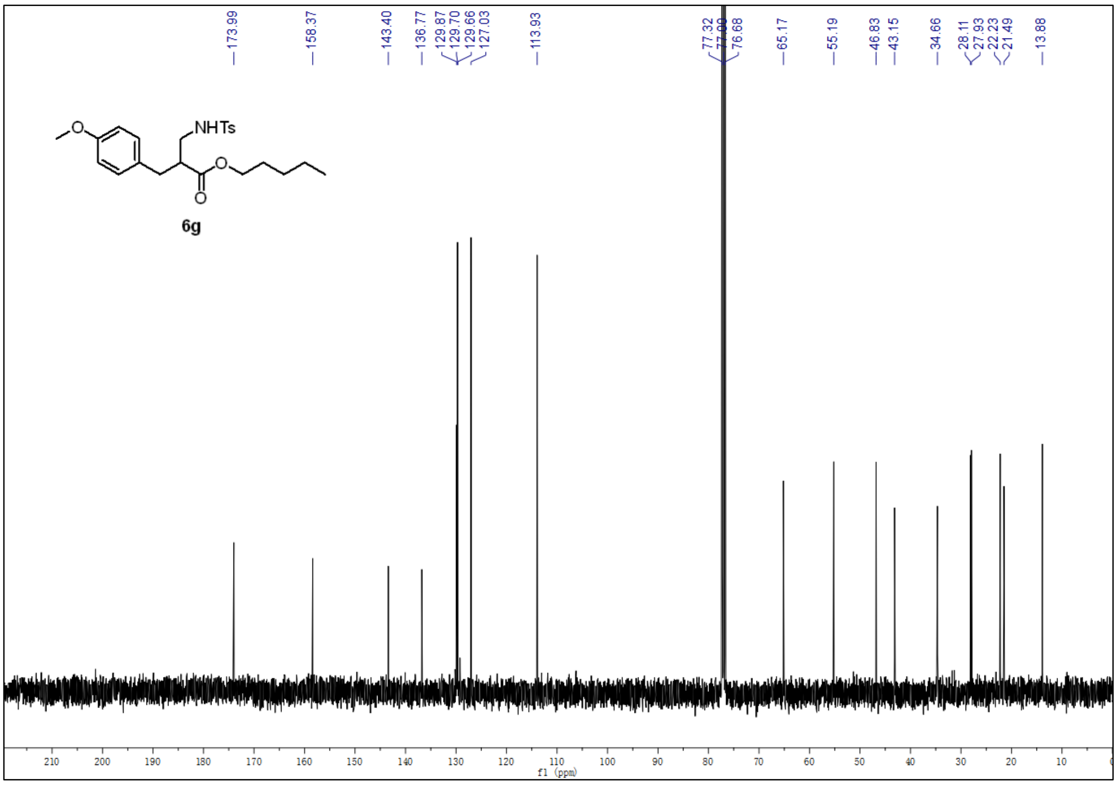

^{1}H NMR (400 MHz, $CDCl_3$) and ^{13}C NMR (101 MHz, $CDCl_3$) spectrum of 6h

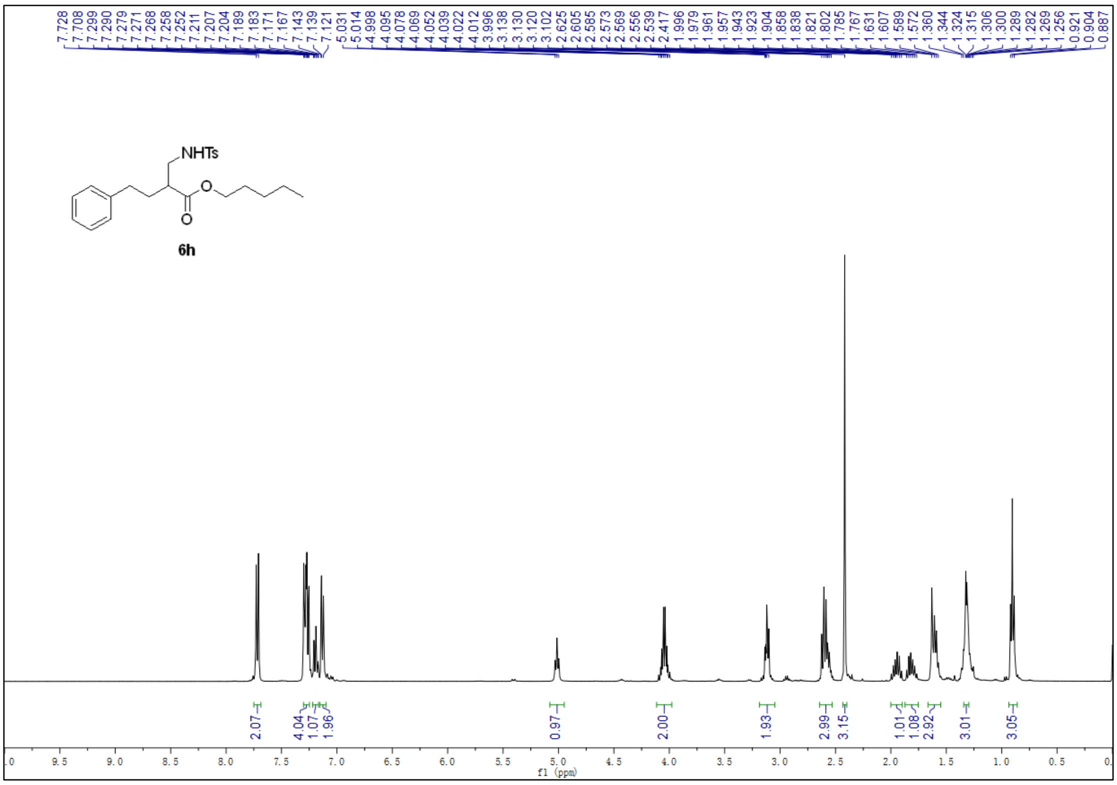

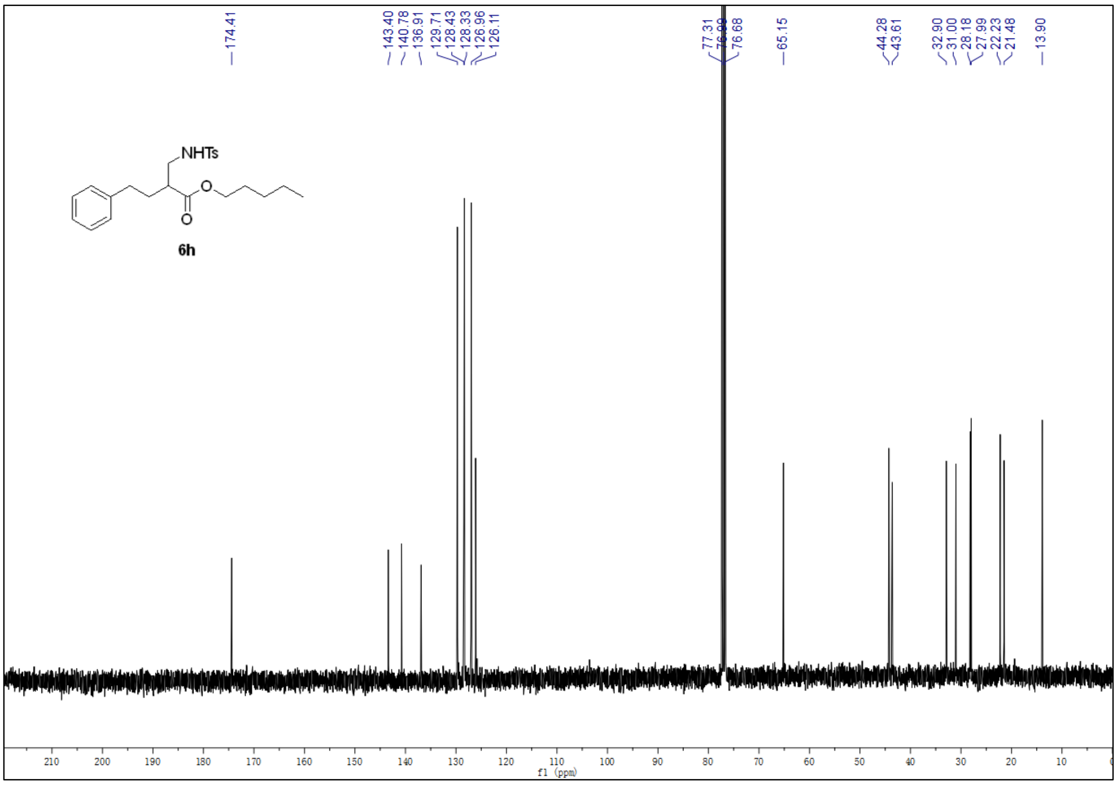

^{1}H NMR (400 MHz, CDCl3) and ^{13}C NMR (101 MHz, CDCl3) spectrum of 6i

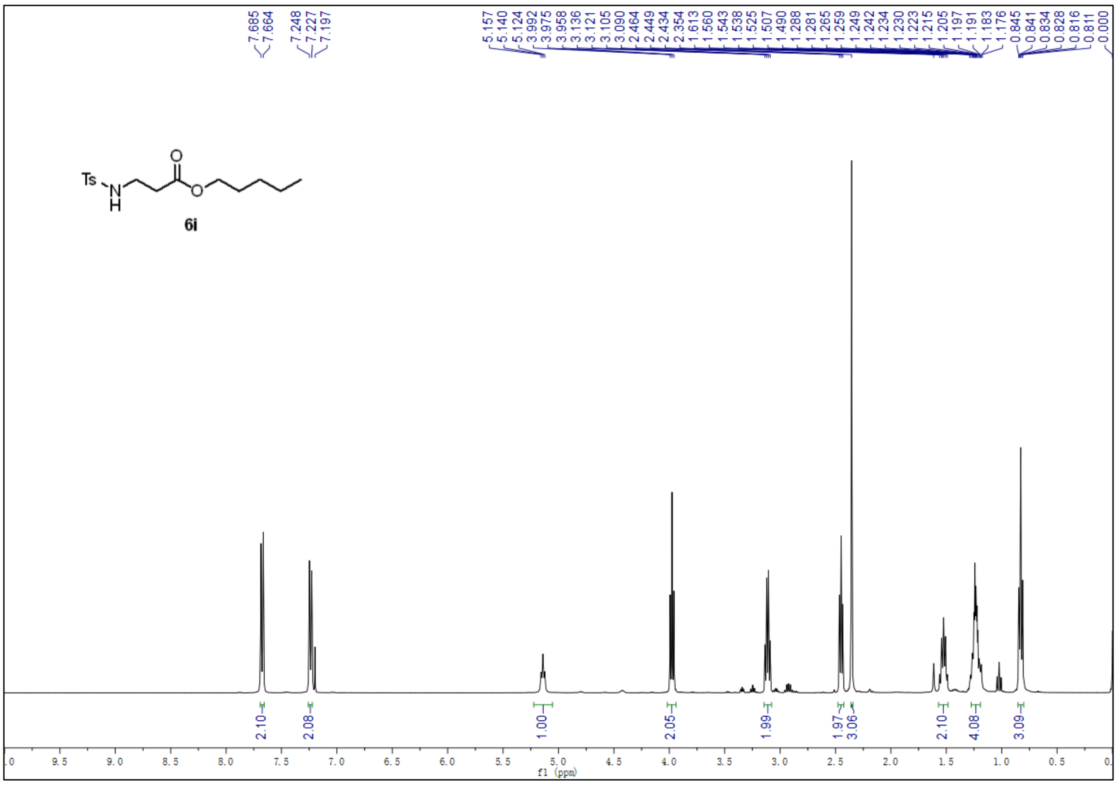

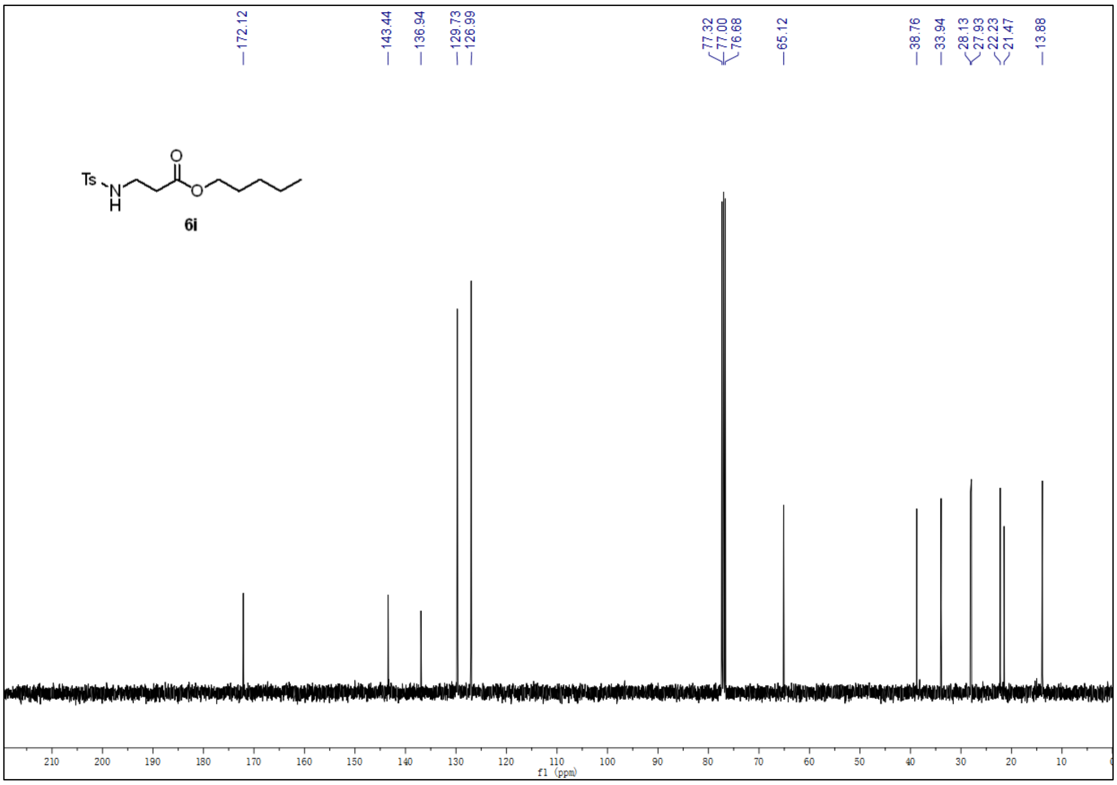

^{1}H NMR (400 MHz, $CDCl_3$) and ^{13}C NMR (101 MHz, $CDCl_3$) spectrum of 6j

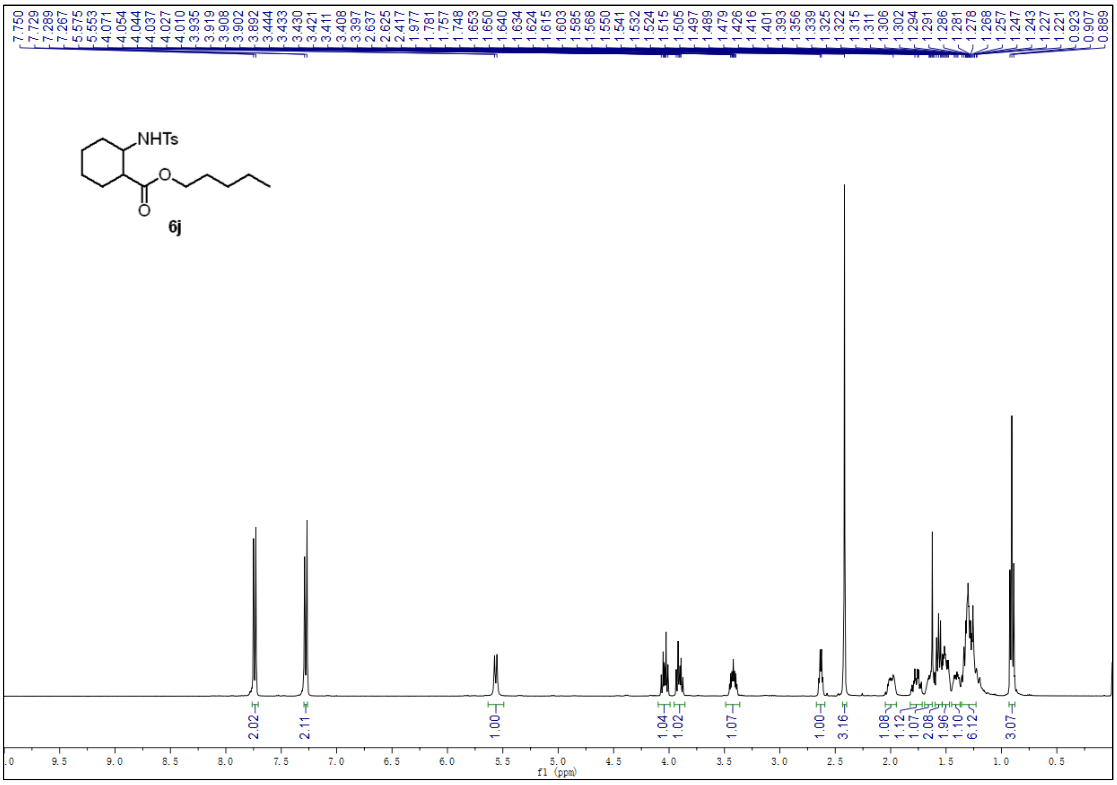

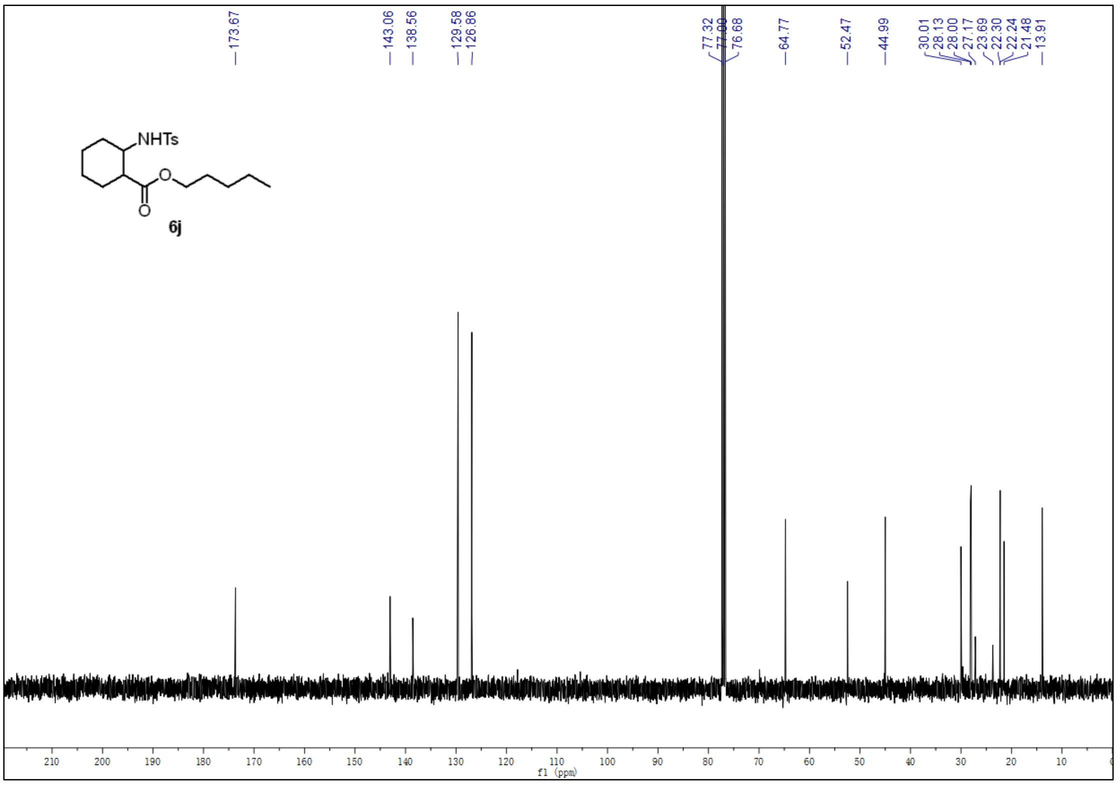

^{1}H NMR (400 MHz, $CDCl_3$) and ^{13}C NMR (101 MHz, $CDCl_3$) spectrum of 6k

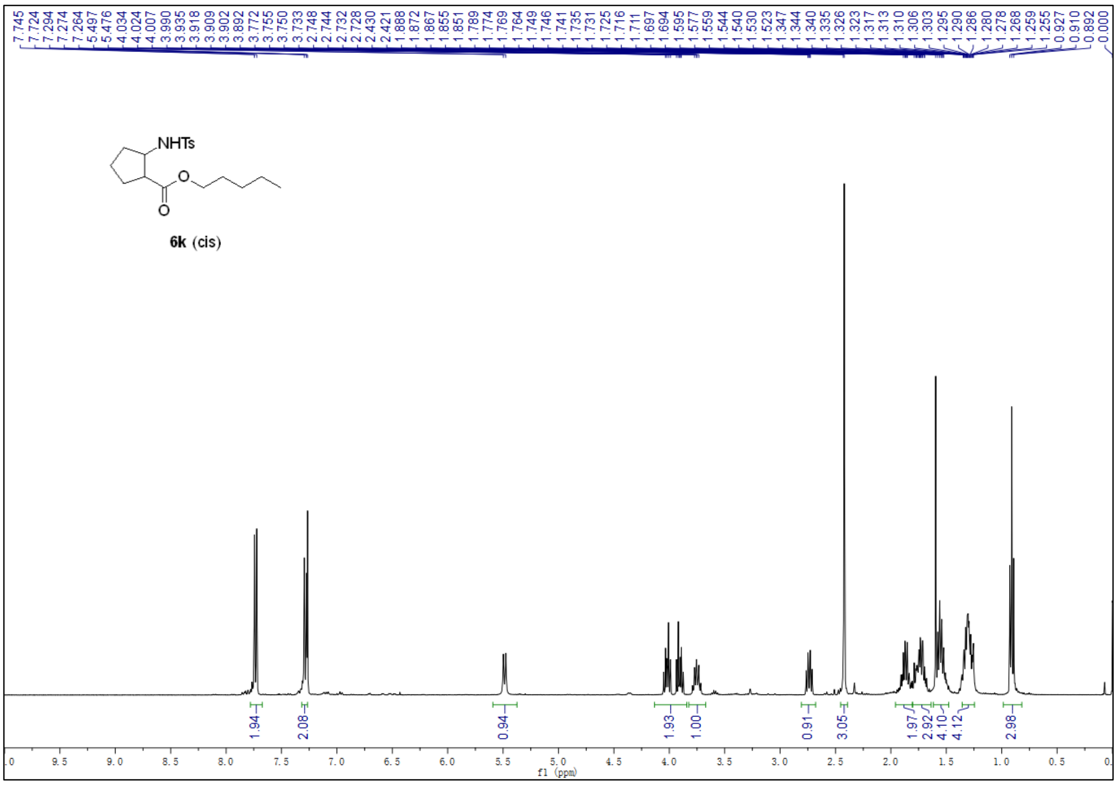

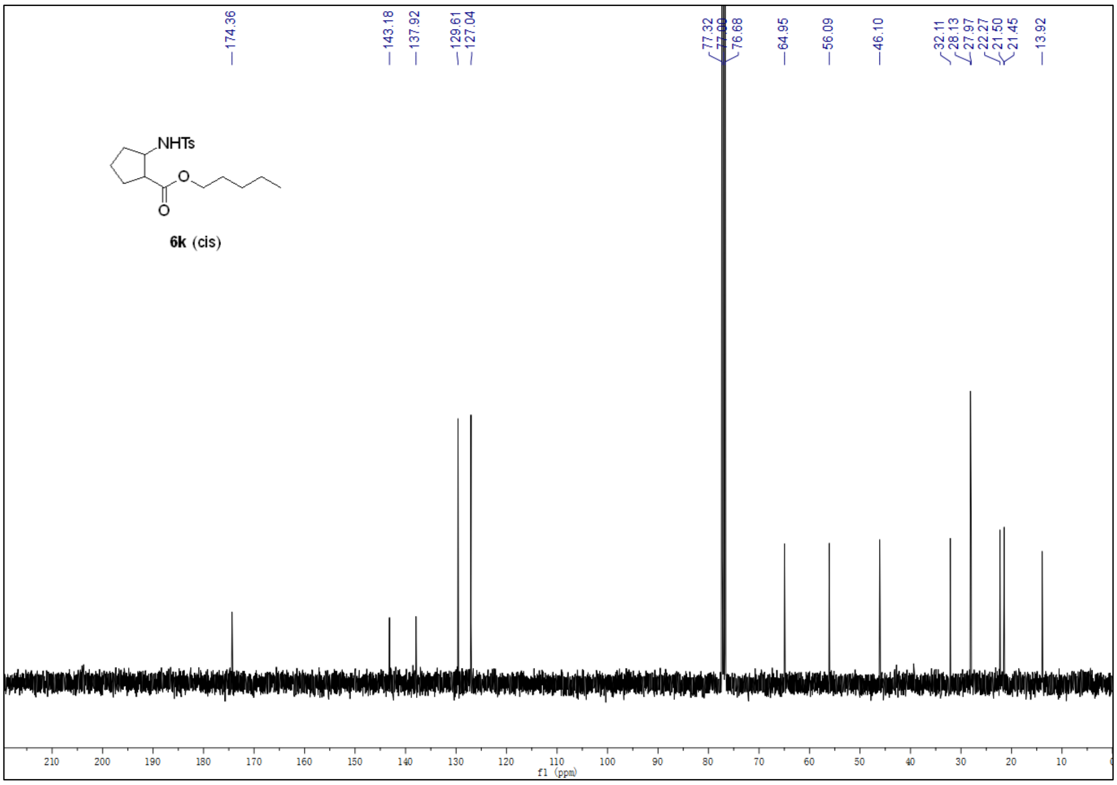

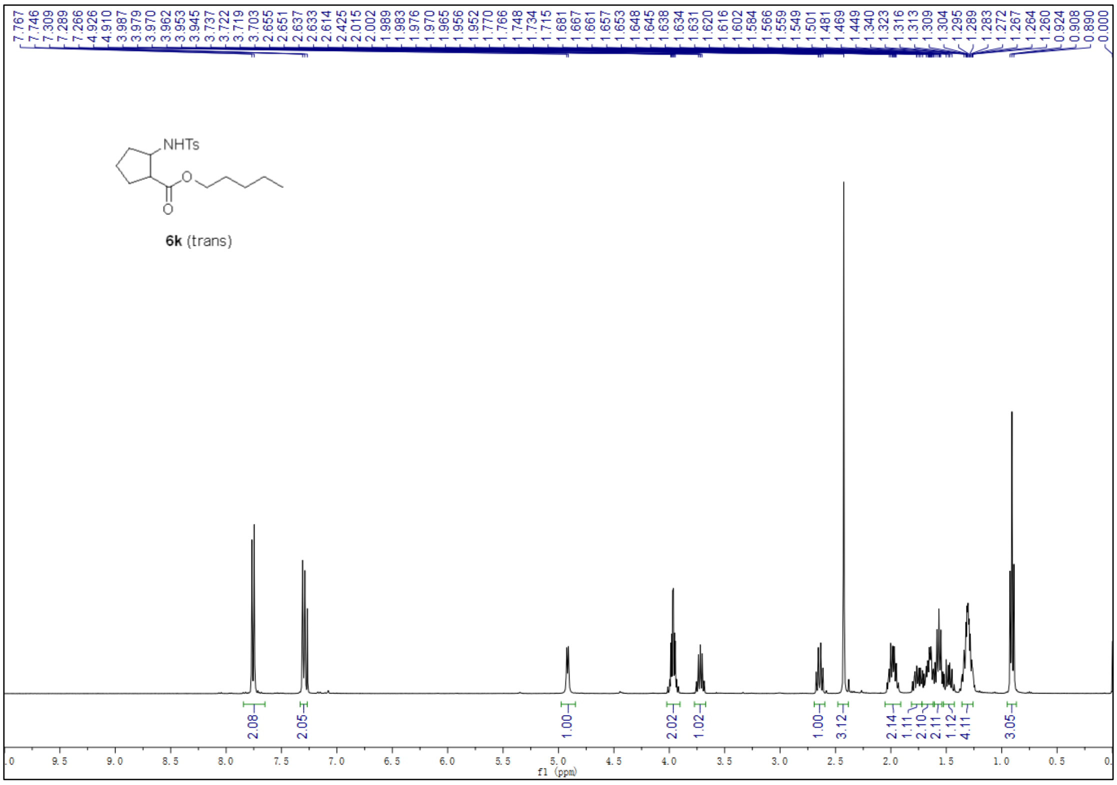

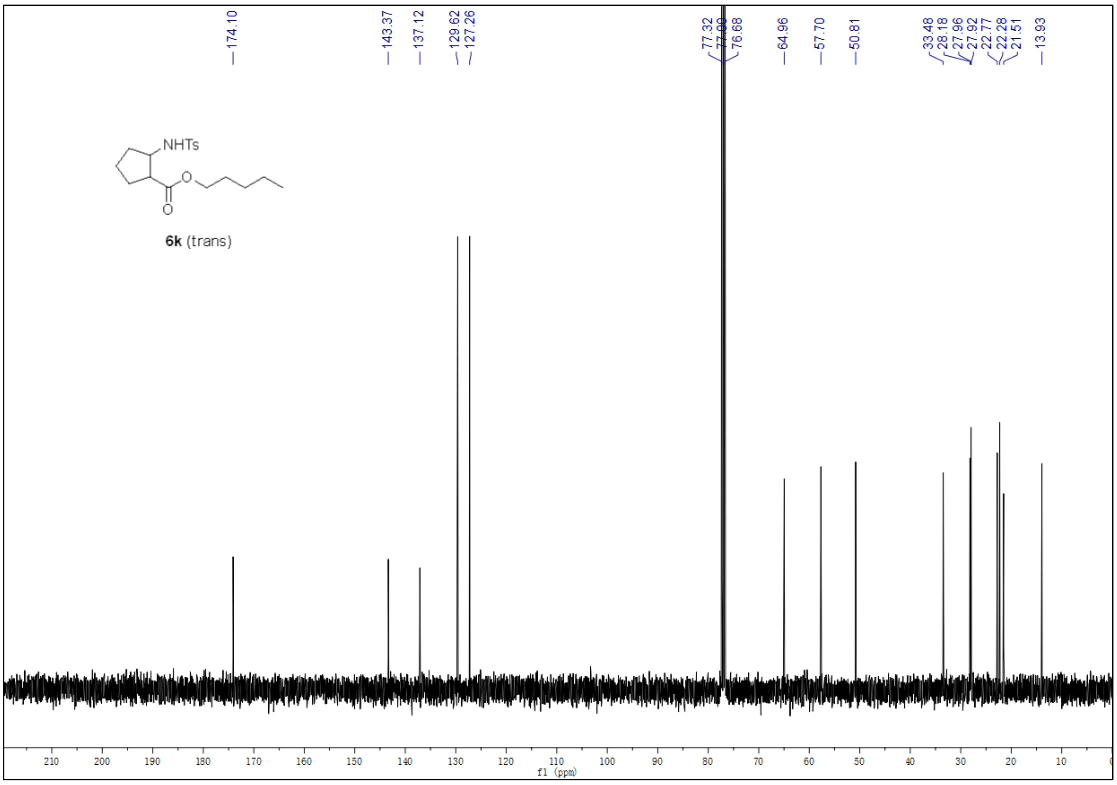

^{1}H NMR (700 MHz, CDCl3) and ^{13}C NMR (175 MHz, CDCl3) spectrum of 8

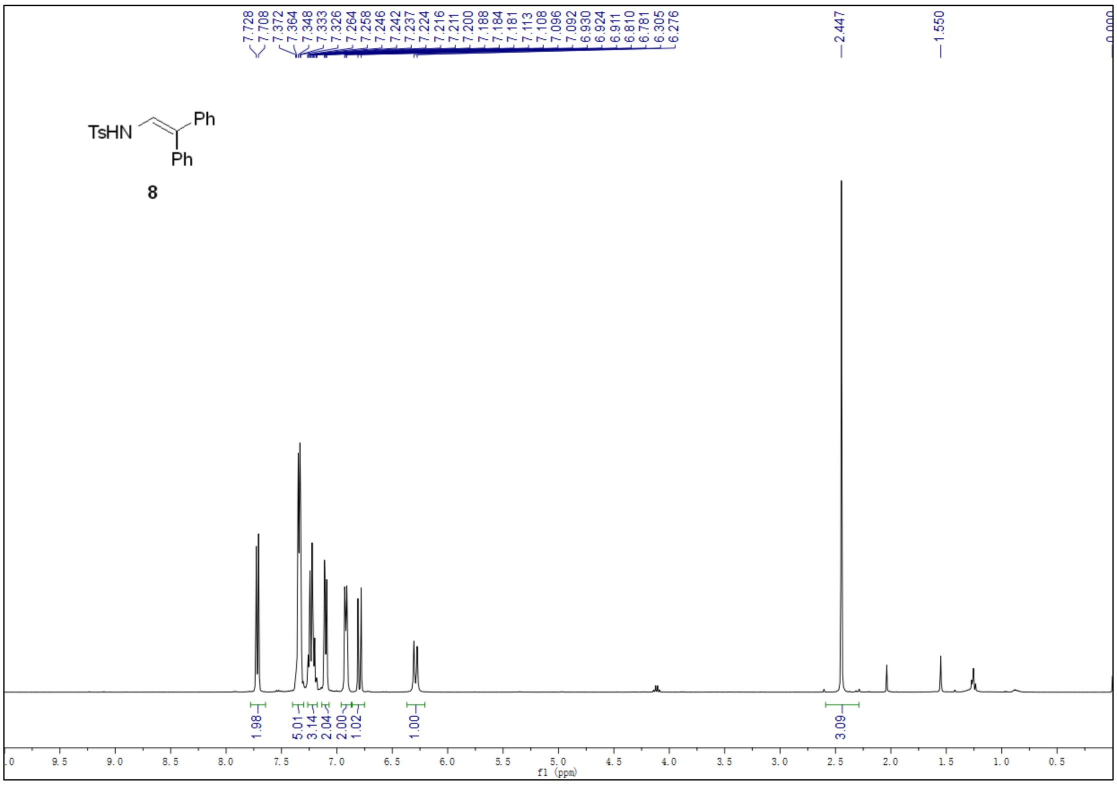

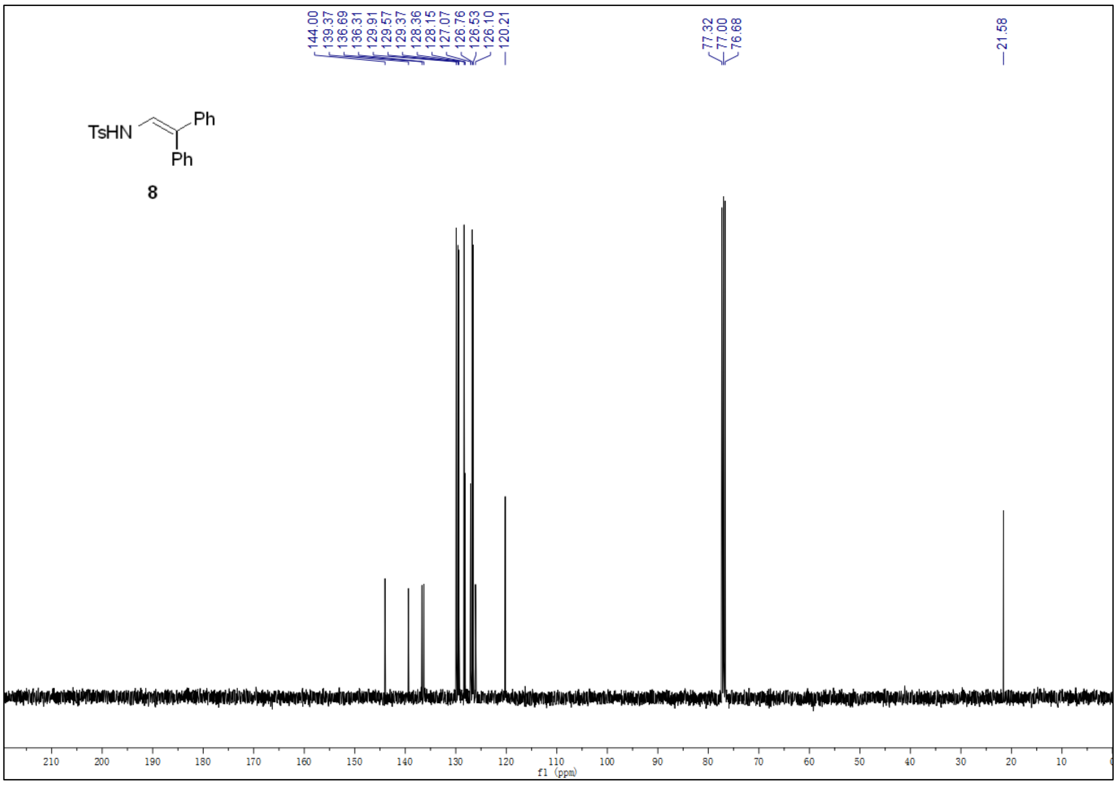

Supplement: Supplementary file 1 [file ol6c00774_si_001.pdf]
